# Genetic diversity and molecular characterization of HRSV-A on the coast of Peru, 2009–2020

**DOI:** 10.3389/fmicb.2026.1704867

**Published:** 2026-06-10

**Authors:** Wilmer Silva-Caso, Miguel Angel Aguilar-Luis, Gustavo Evaristo Ballmann, Alessia Seminario Vittoria, Yordi Tarazona-Castro, Tomás Pumarola Suñe, Angela Cornejo-Tapia, Johanna Martins-Luna, Andrés Antón, Juana del Valle-Mendoza

**Affiliations:** 1 Laboratorios de Biomedicina, Centro de Investigación de la Facultad de Ciencias de la Salud, Universidad Peruana de Ciencias Aplicadas, Lima, Peru; 2 Department of Microbiology, Vall d’Hebron Institut de Recerca, Vall d’Hebron Hospital Universitari, Vall d’Hebron Barcelona Hospital Campus, Universitat Autònoma de Barcelona, Barcelona, Spain; 3 Centro de Investigación Biomédica en Red de Enfermedades Infecciosas (CIBERINFEC), Madrid, Spain

**Keywords:** HRSV, HRSV-A lineages, human respiratory syncytial virus, Peru, phylogenetic, pneumonia, respiratory disease

## Abstract

**Background:**

The objective of this study was to molecularly characterize human respiratory syncytial virus A (HRSV-A) genotypes that circulated on the coast of Peru between 2009 and 2020.

**Methods:**

In total, 1,694 samples were collected from pediatric patients who were hospitalized with acute respiratory infection during the study period. Samples were analyzed by real-time RT-PCR. The second hypervariable region of the G gene was amplified from HRSV-A-positive samples with high viral load and subsequently sequenced for genotyping and molecular characterization. Phylogenetic reconstruction was performed using the maximum likelihood method, and temporal analysis was conducted by Bayesian inference using the Markov chain-based Monte Carlo (MCMC) algorithm.

**Results:**

Of the total samples processed, 341 (20.13%) were positive for HRSV, of which 211 (61.88%) cases were positive for HRSV-A. A total of 53 nucleotide sequences were obtained from the partial G gene, corresponding to the second hypervariable region. Phylogenetic inference together with representative HRSV-A sequences showed the presence of clades assigned to the NA1 (A.3.1 and A.3.1.1) and ON1 (A. D, A. D.1, A. D.1.1, A. D.2.2, and A. D.5) genotypes, with evidence of co-circulation of at least two clades during the same year. A distinct monophyletic subclade within the ON1 clade A. D.1 was also identified in our population and was characterized by three exclusive point mutations (S250F, S267L, and T293I) in the analyzed region of the G protein. In addition, temporal analysis of ON1 sequences estimated an evolutionary rate of approximately 4.59 × 10^−3^ substitutions per site per year in the C-terminal third of the G protein and placed the most recent common ancestor of the Peruvian sequences around 2008.39 [95% HPD: 2006.38, 2009.67], approximately 1 year before the earliest ON1-positive samples from our study population.

**Conclusion:**

HRSV-A showed genetic diversity on the coast of Peru, with co-circulation of NA1- and ON1-related clades during the study period. Partial G gene analysis also identified a putative autochthonous variant within ON1 clade A. D.1, characterized by distinctive changes in the amino acid profile, and provided temporal evidence consistent with ON1 diversification. These findings should be interpreted in light of the partial nature of the sequenced region; however, they provide a foundation for molecular surveillance and future genomic studies of RSV in Peru.

## 1 Introduction

The human respiratory syncytial virus (HRSV) belongs to the family Pneumoviridae and is one of the main causes of bronchiolitis and severe respiratory disease in infants and young children. It is also an important cause of respiratory infections in immunocompromised patients and the elderly (Buchan et al., 2023; Falsey et al., 2005; Jain et al., 2023). This highly contagious virus is responsible for severe episodes of bronchiolitis and pneumonia, conditions that may endanger the lives of the most vulnerable groups (Kaler et al., 2023). At a global level, HRSV is associated with a significant disease burden, including hospitalization and mortality, which highlights the need for continuous surveillance and molecular characterization of the virus to better understand its epidemiological behavior and evolution (Kaler et al., 2023; Rios Guzman and Hultquist, 2022).

In Peru, HRSV is an important public health concern, as it may be responsible for between 16 and 86% of severe acute respiratory infection cases requiring hospitalization during certain times of the year (Ramírez-Soto et al., 2022; Del Valle Mendoza et al., 2015). HRSV infections in Latin American countries have shown a marked seasonality. In the Southern Hemisphere, incidence peaks occur during the coldest months, from June to July, and show late peaks until the first week of August. HRSV activity declines with decreasing temperature, and the epidemic season ends around the end of September or the beginning of October (Obando-Pacheco et al., 2018). This epidemic pattern emphasizes the need for studies to assess the circulation of different HRSV genotypes and correlate them with epidemiological data, especially in specific regions where climate and demographic conditions may influence the viral dynamics (Vandini et al., 2017).

There are two main HRSV groups, A and B, which may both coexist during the epidemic season. Recent studies have shown that both HRSV A and B exhibit a general chronological evolutionary pattern. Furthermore, it has been estimated that the evolutionary rate of HRSV B is higher than that of HRSV A (Yun et al., 2020; Guo et al., 2023; Piñana et al., 2024). It has been reported that the infection caused by this group may result in more severe clinical disease, with a larger percentage of patients requiring mechanical ventilation and intensive care (Laham et al., 2017; Vila et al., 2023; Jafri et al., 2013; Bender et al., 2024). Therefore, the molecular characterization of circulating HRSV strains is essential for understanding their genetic diversity, evolutionary dynamics, and potential implications for the development of effective vaccines and therapies (Kim et al., 2023). Within this context, the identification of emerging HRSV-A genetic groups, particularly those associated with rapidly diversifying lineages, is relevant for molecular surveillance and public health (Vandini et al., 2017).

At the genomic level, HRSV is a negative-sense single-stranded RNA virus with a genome of approximately 15.2 kb that encodes 11 viral proteins (F, G, SH, M2-1, M2-2, N, L, P, M, NS1, and NS2) (Anderson et al., 2024). Among its surface glycoproteins, the attachment glycoprotein G is one of the most variable and has therefore been widely used in molecular epidemiology studies (McLellan et al., 2013). The ectodomain of this protein contains two mucin-like hypervariable regions separated by a central conserved domain, and much of HRSV diversification has been associated with the accumulation of nucleotide substitutions, amino acid changes, variations in glycosylation patterns, and duplication events in this protein (Eshaghi et al., 2012), particularly within the C-terminal region. Taken together, these features make the G gene a particularly informative target for tracking viral genetic diversity and the spread of emerging variants over time (Duvvuri et al., 2015).

Accordingly, variability within the C-terminal portion of the G gene, which includes the second hypervariable region targeted in this study, has historically been used to distinguish genetic groups, describe the circulation of known genotypes, and identify emerging genotypic variants. This approach has been particularly useful for identifying major evolutionary events, such as the 60-nucleotide duplication in RSV-B that gave rise to the BA genotype, as well as the 72-nucleotide duplication in RSV-A that gave rise to the ON1 genotype (Eshaghi et al., 2012; Duvvuri et al., 2015; Peret et al., 2000; Zlateva et al., 2004), currently encompassed within the A. D. lineage framework. However, more recent classification frameworks recommend the use of broader genomic regions, particularly the complete G ectodomain or whole genomes, to improve phylogenetic resolution and lineage assignment (Muñoz-Escalante et al., 2019; Goya et al., 2020; Goya et al., 2024). In this context, partial G gene sequences remain useful for phylogenetic contextualization and comparison with annotated reference strains; however, interpretations based solely on the second hypervariable region should be made with caution because of its lower phylogenetic signal, and the definition of new lineages or sublineages should be reserved for analyses based on whole genomes.

Within the Peruvian context, previous studies have reported that HRSV-A is one of the most frequent viral etiologies in pediatric populations, particularly in Lima (del Valle Mendoza et al., 2015), and that it has also been the most frequently detected RSV subtype among hospitalized children and severe cases (Becerra et al., 2019). Similarly, although RSV has been studied in the country mainly from a clinical and epidemiological perspective, studies aimed at its genotyping and molecular characterization remain limited. In this context, the present study focused on HRSV-A to characterize its genetic diversity.

The objective of this study was to conduct the molecular characterization of representative samples of HRSV-A strains that circulated on the Peruvian coast between 2009 and 2020 through partial sequencing of the glycoprotein (G) gene, phylogenetic analysis, and amino acid profile analysis. These analyses not only allowed the identification of viral clades circulating during the study period but also provided valuable information on possible local variants within clusters related to lineage A. D, which could have significant implications for the epidemiology of HRSV and disease control and prevention strategies.

## 2 Materials and methods

### 2.1 Design and sample

A retrospective cross-sectional study was conducted in which 1,694 samples from pediatric patients aged 0–17 years who were hospitalized with acute respiratory infection between 2009 and 2020 in the coastal Peruvian regions of Lima, Piura, and Arequipa were analyzed. Samples were initially analyzed using real-time RT-PCR for HRSV detection, and downstream molecular analyses were restricted to HRSV-A-positive samples to characterize the genetic diversity of this subgroup. Molecular characterization was performed by partial sequencing of the glycoprotein (G) gene, targeting the second hypervariable region.

The Research Ethics Committee of the Universidad Peruana de Ciencias Aplicadas (EC No. PI182-21) evaluated and approved this study, which was conducted according to the 2013 revision of the 1975 Declaration of Helsinki. All safety measures were implemented to guarantee the confidentiality of patient identification data. Informed consent was not required, as this study was a retrospective analysis using samples and a database without personal identification information.

### 2.2 Detection of HRSV and HRSV-A by real-time RT-PCR

Nasopharyngeal swab samples were stored in universal transport medium (UTM; Copan Diagnostics) at −80 °C in the Biobank of the Laboratory of Biomedicine, Universidad Peruana de Ciencias Aplicadas, until viral genetic material extraction. Viral RNA was purified from 200 μL of each nasopharyngeal swab sample using the High Pure Viral RNA Isolation kit (Roche Applied Science, Mannheim, Germany) according to the manufacturer’s instructions. The RNA eluate was recovered in 80 μL of RNase-free elution buffer (10 mM Tris–HCl, pH 8.5) and stored at −80 °C until use.

Detection of HRSV and HRSV-A subtype was performed by real-time RT-PCR using the primers and probes designed by Reiche and Schweiger (2009) and Liu et al. (2016), respectively (Supplementary Table S1; Reiche and Schweiger, 2009; Liu et al., 2016). The master mix for each RT-PCR reaction was prepared according to the instructions of the LightCycler Multiplex RNA Virus Master kit (Roche Diagnostics, Deutschland-Mannheim, Germany), using 10 μM of primers and probes, and 5 μL of RNA for a final reaction volume of 20 μL. In both cases, reverse transcription was carried out at 50 °C for 10 min; predenaturation was performed at 95 °C for 30 s; and amplification cycling consisted of 50 cycles of denaturation at 95 °C for 5 s, annealing at 56 °C for 30 s, and 15 s of extension at 72 °C. A positive sample and nuclease-free water were used as amplification and contamination controls, respectively. Samples with a clear sigmoidal amplification curve and Ct ≤ 38 were considered positive for HRSV or HRSV-A; those with no amplification or Ct > 38 were considered negative, according to the interpretation criteria established by previous studies and the WHO (World Health Organization, 2019) and PAHO (Pan American Health Organization, 2021) diagnostic guidelines for real-time RT-PCR assays of respiratory viruses.

### 2.3 cDNA synthesis, PCR, and sequencing of the partial G gene

cDNA synthesis was performed in all HRSV-A positive cases that presented a high relative viral load (CT ≤ 25). The synthesis was carried out according to the instructions of the Transcriptor First Strand cDNA Synthesis kit (Roche Diagnostics, Mannheim, Germany), using 5 μL of RNA for a final mixing volume of 20 μL.

Amplification of the partial gene corresponding to the second hypervariable region of HRSV-A was performed by a nested PCR reaction, using the primers designed by Reiche and Schweiger (2009; Supplementary Table S1), and the LightCycler FastStart DNA Master HybProbe kit master mix (Roche Diagnostics, Deutschland-Mannheim, Germany), in a final reaction volume of 25 μL. For the first-round PCR, 4 μL of cDNA was used as a template, and the amplification conditions for outer PCR were set with predenaturation at 95 °C for 10 min, followed by 50 cycles of amplification at 95 °C for 30 s, annealing at 58 °C for 30 s, and extension at 72 °C for 1 min. Finally, a final extension was performed at 72 °C for 10 min. The nested PCR was performed using 2 μL of the first-round PCR product as template and was carried out under the same amplification conditions as the outer PCR, except for the annealing temperature, which was set to 53 °C. Both were run in an Applied Biosystems Veriti thermal cycler.

The amplification products of approximately 460 bp and 390 bp were visualized on a 2% agarose gel, using the fluorescent dye Nancy-520 (Sigma-Aldrich) and a UV transilluminator. Only the PCR products that presented high intensity bands were purified following the protocol of the SprinPrepTM Gel DNA kit (Novagen, United States). The concentration of the purified PCR products was measured using a NanoDrop™ One spectrophotometer (Thermo Scientific™), and only those samples with a concentration ≥ 30 ng/μL were sequenced in both directions, using the sequencing primers described in Supplementary Table S1, by the Sanger method on an ABI 3730xl automated capillary electrophoresis sequencer by Macrogen (Macrogen, Inc., South Korea).

### 2.4 Analysis of sequences and datasets

View of electropherograms and sequence curation were performed with the CodonCode Aligner version 10.0.2 software (CodonCode Corporation, Dedham, MA, United States). The consensus nucleotide sequences comprised the G protein’s C-terminal region and part of the F protein’s N-terminal region. For this study, analyses were restricted to the second hypervariable region of the G gene, located between codons 210 and 297 with respect to the oldest prototype strains of HRSV-A (GenBank accession numbers: M11486, AAB59857, NC_001803, NP_044595). This fragment was used for phylogenetic contextualization of the sequences obtained in this study within annotated HRSV-A genetic groups, as well as for a temporal analysis limited to the approximate estimation of the tMRCA of the earliest ON1 genotype sequences identified in this study. Given the lower phylogenetic resolution of this region compared with the complete G ectodomain or whole genomes, the resulting inferences were interpreted cautiously and were not used for formal lineage designation, phylogeographic reconstruction, inference of dispersal patterns, or broader phylodynamic interpretation.

Two datasets were prepared from publicly available HRSV-A sequences. In both cases, the homologous fragment of the second hypervariable region of the G gene was extracted from complete G gene sequences or whole genomes. The first dataset was intended for maximum-likelihood phylogenetic reconstruction. Initially, 3,389 sequences reported through December 2024 were retrieved from GenBank (NCBI), corresponding to complete G-gene sequences or whole genomes representative of current HRSV-A phylogenetic diversity annotated in Pathoplexus.^1^ After extraction of the homologous HVR2 fragment, identical sequences were removed using SeqKit v2.12.0 (Shen et al., 2024). Redundancy was then reduced from a preliminary phylogenetic tree using Treemmer v0.3 (Menardo et al., 2018) with a relative tree length (RTL) threshold of 0.95, while preserving a broad representation of the phylogenetic diversity of the initial dataset. The final reference dataset comprised 657 sequences (Supplementary Table S2).

For the second dataset, intended for temporal analysis, an initial representative subsampling of 3,275 sequences of the HRSV-A ON1 genotype with known sampling date and location was carried out from records available up to December 2024 in GenBank and GISAID, corresponding to complete G gene sequences or whole genomes representative of the phylogenetic diversity currently annotated for ON1 in Pathoplexus and GISAID-EpiRSV.^2^ In this selection, the earliest available sequences from each country were prioritized to preserve temporal and geographic representativeness. Subsequently, the homologous fragment corresponding to the second hypervariable region of the G gene was extracted, and sequences with gaps or ambiguities were excluded. In addition, identical sequences from the same origin and year were removed using SeqKit v2.12.0, and sequences behaving as temporal outliers during temporal signal assessment were further discarded. The final dataset comprised 771 sequences (Supplementary Table S3).

### 2.5 Phylogenetic analysis

Multiple sequence alignment of the nucleotide sequences obtained in this study, together with the reference sequences included in each dataset, was performed using the MUSCLE algorithm implemented in MEGA v11.0 software (Tamura et al., 2021). The alignments had a length of 333 bp, corresponding to the second hypervariable region of the G gene. The possibility of recombination events in the alignments was assessed using RDP4 (Martin et al., 2015), applying the RDP, GENECONV, BootScan, MaxChi, Chimaera, SiScan, and 3Seq methods with default settings, a *p*-value threshold of 0.05, and Bonferroni correction. A recombination event was considered supported when it was detected by at least three of the methods used.

The best-fitting nucleotide substitution model for the analyzed sequence datasets was determined using jModelTest2 v2.1.10 (Darriba et al., 2012) under the Bayesian Information Criterion (BIC), which selected the GTR + G model. Phylogenetic reconstruction of HRSV-A by the maximum-likelihood (ML) method was performed using IQ-TREE v2.4.0 (Minh et al., 2020). Branch support was assessed with 1,000 ultrafast bootstrap replicates and SH-aLRT. Clusters with bootstrap values ≥ 80 were considered well supported, in accordance with the threshold proposed by Goya et al. (2020). The resulting consensus trees were visualized in FigTree v1.4.2 (Rambaut, 2018).

For phylogenetic contextualization of the sequences obtained in this study, the classification framework proposed by the HRSV Genotyping Consensus Consortium (RGCC) was adopted as the current reference for HRSV-A classification within the subgroup. Under this standardized nomenclature, ancestral lineages are designated as A (A.1-A.3), whereas strains carrying the 72-nt duplication, historically referred to as the ON1 genotype, are classified within lineage A. D. (A. D.1-A. D.5). Nested lineages or sublineages are represented by increasing ordinal numbers separated by periods within each major lineage, according to Goya et al. (2024).

### 2.6 Estimation of genetic distances

Genetic divergence among the HRSV-A groups evaluated in this study was assessed using the p-distance method, which estimates the average genetic distance between nucleotide pairs (number of nucleotide differences/total sequence length). The average genetic distance matrix was calculated using the Distance tool in MEGA v11.0 under a 4-category gamma distribution with invariant sites (G4 + I), excluding sites with alignment gaps and missing data. Variance was estimated with 1,000 bootstrap replicates.

Because our analyses were restricted to the second hypervariable region of the G gene, pairwise genetic distances were used as a comparative descriptive measure to assess the divergence of the study cluster relative to the annotated lineages of the ON1 genotype, rather than as a criterion for formal lineage or sublineage designation.

### 2.7 Deducted amino acid analysis and glycosylation site prediction

The 333-bp nucleotide sequences corresponding to the second hypervariable region of the HRSV-A G gene were translated into amino acid sequences in MEGA v11.0. To evaluate the nonsynonymous changes present in the sequences obtained in this study, previously annotated reference sequences were used, whose amino acid patterns within the second hypervariable region allowed comparison of the study sequences with annotated HRSV-A lineages according to their prior phylogenetic placement and the reference framework proposed by Goya et al. (2024). Changes observed within lineage A. D. (ON1) were analyzed with respect to the prototype strain ON67-1210 (GenBank accession number: JN257693), whereas changes within lineage A.3.1/A.3.1.1, which historically included genotype NA1, were analyzed with respect to the prototype strain NG-016-04 (GenBank accession number: AB470478). Potential N-glycosylation and O-glycosylation sites were estimated through the NetNGlyc 1.0 server (Gupta and Brunak, 2002) under the supposition that the G protein’s C-terminal third sequence was Asn-Xaa-Ser/Thr (except for Asn-Pro-Ser/Thr) and NetOGlyc 4.0 (Steentoft et al., 2013), where serine and threonine residues are predicted to be O-linked oligosaccharide binding sites (Julenius et al., 2005). Predicted glycosylation sites were those with a score greater than or equal to the predefined threshold of 0.5 in both analyses.

### 2.8 About the data used in this study

Sequences of the G protein’s partial gene that were obtained in this study were stored in the GenBank database with accessions [PQ468040-PQ468092]. In the analyses, all sequences isolated in this study were tagged with the prefix “PER,” while public sequences downloaded from the GenBank database and used as reference sequences were tagged with their respective accession code.

### 2.9 Estimation of the tMRCA of ON1 sequences analyzed in this study

To obtain an approximate estimate of the time to the most recent common ancestor (tMRCA) of the earliest ON1 genotype-related sequences identified in this study, based on the fragment corresponding to the second hypervariable region of the G gene, the temporal dataset of 771 sequences with known collection dates from different countries was used (Supplementary Table S3).

Temporal signal was first explored by root-to-tip regression against sampling time using TempEst v1.5.3 (Rambaut et al., 2016). This analysis was used to assess the temporal structure of the dataset, identify potential temporal outliers, and obtain an exploratory approximation of the tMRCA from the regression intercept.

Subsequently, formal temporal inference was performed in BEAST v1.10.4 (Suchard et al., 2018) using a GTR + G nucleotide substitution model, an uncorrelated relaxed molecular clock, and an exponential growth demographic model. The MCMC chain was run for 200 million generations, with a 10% burn-in. Convergence and posterior stability were assessed in Tracer v1.7.2, and effective sample sizes (ESS) > 200 were considered indicative of satisfactory convergence. The maximum clade credibility (MCC) tree was generated with TreeAnnotator v1.10.4 and visualized in FigTree v1.4.4.

## 3 Results

### 3.1 Detection of HRSV and HRSV-A

During the years in which the study was conducted, a total of 1,694 nasopharyngeal swab samples were analyzed by real-time RT-PCR. Individualized year-by-year analysis indicated that in 2009 and 2010, HRSV-positive samples were 14.23 and 17.67%, respectively, in relation to the total for each year. In both years, the frequency of the HRSV-A subtype was 100 and 95.5%. Since 2018, an increase in the annual prevalence of the HRSV virus has been observed, with a decrease in the HRSV-A subtype. Between 2018 and 2020, the overall positivity for HRSV reached 35.97% in 2018, while in 2019, the RSV-A subtype reached 51.06%. By 2020, the number of HRSV-A cases had decreased, reaching 31.81% of the total HRSV cases, as detailed in Table 1.

**Table 1:** Frequency of HRSV-A cases in the study period.

Year	Region	Sampling period	Samples collected by region	Total collected samples (n)	HRSV cases *n* (%)*	HRSV-A cases *n* (%)**
2009	Lima	January–December	239	239	34 (14.23)	34 (100.00)
2010	Lima	January–December	498	498	88 (17.67)	84 (95.45)
2011	Lima	January–December	130	130	3 (2.31)	3 (100.00)
2012	Lima	January–December	103	103	0 (0.00)	0 (0.00)
2018	Lima Piura	January–December	176 102	278	100 (35.97)	35 (35.00)
2019	Lima Piura Arequipa	January–December	237 108 28	373	94 (25.20)	48 (51.06)
2020	Lima	January–June	73	73	22 (30.14)	7 (31.81)
			Total	1,694	341 (20.13)	211 (61.88)

* (HRSV cases/Total collected samples) x 100. ** (HRSV-A cases / HRSV cases) x 100.

### 3.2 Alignment of sequences and phylogenetic analysis

A total of 53 partial sequences of the second hypervariable region of the HRSV-A G gene were obtained (Table 2) between May 2009 and April 2020. Multiple alignment of these sequences with the first reference dataset indicated that the best-fitting nucleotide substitution model was GTR + G, with four gamma rate categories. Furthermore, no recombination events were detected in the alignments of our datasets (Supplementary Table S4).

**Table 2:** HRSV-A G gene sequences obtained in this study.

Isolates	GenBank accession number	Detection place	Detection date	Genotype	Clade
PER5.03_2010	PQ468043	Lima	11/03/2010	NA1	A.3.1.1
PER9.05_2010	PQ468047	Lima	8/05/2010	NA1	A.3.1
PER10.05_2010	PQ468048	Lima	18/05/2010	NA1	A.3.1.1
PER11.05_2010	PQ468049	Lima	17/05/2010	NA1	A.3.1
PER39.07_2019	PQ468078	Lima	25/07/2019	NA1	A.3.1
PER40.08_2019	PQ468079	Arequipa	14/08/2019	NA1	A.3.1
PER41.08_2019	PQ468080	Arequipa	14/08/2019	NA1	A.3.1
PER1.05_2009	PQ468040	Lima	5/05/2009	ON1	A. D
PER3.05_2009	PQ468041	Lima	20/05/2009	ON1	A. D
PER4.11_2009	PQ468042	Lima	23/11/2009	ON1	A. D
PER6.03_2010	PQ468044	Lima	23/03/2010	ON1	A. D
PER7.04_2010	PQ468045	Lima	19/04/2010	ON1	A. D.1.1
PER8.05_2010	PQ468046	Lima	7/05/2010	ON1	A. D.1.1
PER12.05_2010	PQ468050	Lima	16/05/2010	ON1	A. D.1.1
PER13.06_2010	PQ468051	Lima	11/06/2010	ON1	A. D.1.1
PER14.06_2018	PQ468052	Lima	15/06/2018	ON1	A. D.1
PER15.06_2018	PQ468053	Lima	15/06/2018	ON1	A. D.1
PER16.06_2018	PQ468054	Lima	15/06/2018	ON1	A. D.1
PER17.06_2018	PQ468055	Piura	15/06/2018	ON1	A. D.1
PER18.06_2018	PQ468056	Piura	15/06/2018	ON1	A. D.2.2
PER19.06_2018	PQ468057	Piura	15/06/2018	ON1	A. D.1
PER20.06_2018	PQ468058	Piura	15/06/2018	ON1	A. D.1
PER21.06_2018	PQ468059	Lima	15/06/2018	ON1	A. D.2.2
PER2.06_2018	PQ468060	Lima	5/06/2018	ON1	A. D.2.2
PER22.06_2018	PQ468061	Lima	15/06/2018	ON1	A. D.2.2
PER23.07_2018	PQ468062	Lima	4/07/2018	ON1	A. D.2.2
PER24.07_2018	PQ468063	Lima	4/07/2018	ON1	A. D.2.2
PER25.07_2018	PQ468064	Lima	26/07/2018	ON1	A. D.1.1
PER26.08_2018	PQ468065	Lima	22/08/2018	ON1	A. D.1
PER27.10_2018	PQ468066	Piura	12/10/2018	ON1	A. D.1
PER28.04_2019	PQ468067	Lima	24/04/2019	ON1	A. D.1
PER29.04_2019	PQ468068	Lima	24/04/2019	ON1	A. D.1
PER30.04_2019	PQ468069	Piura	24/04/2019	ON1	A. D.1
PER31.04_2019	PQ468070	Piura	24/04/2019	ON1	A. D.1
PER32.07_2019	PQ468071	Lima	2/07/2019	ON1	A. D.1
PER33.07_2019	PQ468072	Lima	2/07/2019	ON1	A. D.1
PER34.07_2019	PQ468073	Lima	11/07/2019	ON1	A. D.1
PER35.07_2019	PQ468074	Lima	11/07/2019	ON1	A. D.1
PER36.07_2019	PQ468075	Lima	11/07/2019	ON1	A. D.1
PER37.07_2019	PQ468076	Lima	25/07/2019	ON1	A. D.1
PER38.07_2019	PQ468077	Lima	25/07/2019	ON1	A. D.1
PER42.09_2019	PQ468081	Piura	10/09/2019	ON1	A. D.1
PER43.09_2019	PQ468082	Piura	10/09/2019	ON1	A. D.1
PER44.09_2019	PQ468083	Piura	10/09/2019	ON1	A. D.1
PER45.09_2019	PQ468084	Piura	10/09/2019	ON1	A. D.1
PER46.09_2019	PQ468085	Piura	10/09/2019	ON1	A. D.1
PER47.09_2019	PQ468086	Piura	10/09/2019	ON1	A. D.1
PER48.09_2019	PQ468087	Piura	10/09/2019	ON1	A. D.1
PER49.09_2019	PQ468088	Piura	10/09/2019	ON1	A. D.1
PER50.09_2019	PQ468089	Piura	10/09/2019	ON1	A. D.1
PER51.03_2020	PQ468090	Piura	06/03/2020	ON1	A. D.1
PER52.10_2019	PQ468091	Arequipa	10/10/2019	ON1	A. D.5
PER53.06_2020	PQ468092	Lima	18/06/2020	ON1	A. D.1

**Figure 1:**
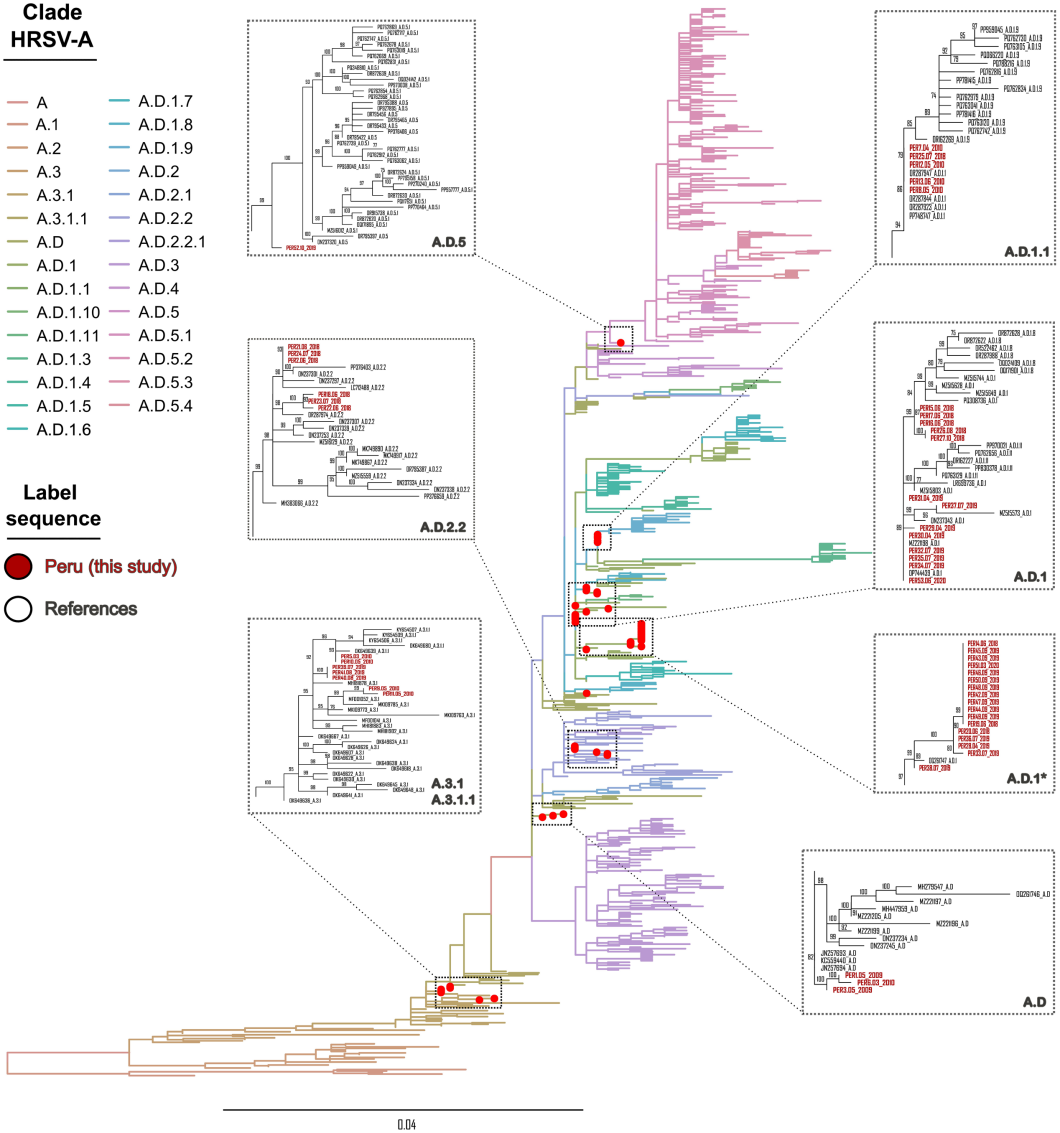
Maximum-likelihood phylogenetic tree of HRSV-A reconstructed with IQ-TREE v2.4 from the second hypervariable region of the G gene under the GTR + G model and 1,000 bootstrap replicates. Bootstrap support values are shown at the nodes. Branches are colored according to the assigned lineages or clades, as indicated in the legend. Sequences generated in this study are labeled in red, whereas reference sequences are shown in black and identified as accession number_annotated lineage. Dashed boxes indicate magnified views of the clades containing the sequences obtained in this study. The scale bar represents the number of substitutions per site per branch.

Maximum-likelihood phylogenetic reconstruction showed that a subset of sequences obtained in this study in 2010 and 2019 clustered within well-supported clades with annotated strains belonging to lineages A.3.1 (*n* = 5) and A.3.1.1 (*n* = 2; Figure 1), which correspond to the historically designated NA1 genotype. Lineage A.3.1 was detected in Lima in 2010 and in Arequipa in 2019, whereas A.3.1.1 was detected in Lima in 2010.

The remaining 46 sequences, obtained during 2009, 2010, 2018, 2019, and 2020, clustered in well-defined clades with annotated strains of lineages A. D. (*n* = 4), A. D.1 (*n* = 30), A. D.1.1 (*n* = 5), A. D.2.2 (*n* = 6), and A. D.5 (*n* = 1), which correspond to the historically designated ON1 genotype, characterized by the 72-nt duplication.

In particular, within lineage A. D.1, a subgroup of 16 sequences detected in Lima and Piura between 2018 and 2020 formed a distinct small monophyletic clade with 99% bootstrap support, which could represent a putative divergent cluster nested within A. D.1, here referred to as A. D.1*. Similarly, lineage A. D.2.2 was detected in Piura in 2018, and lineage A. D.5 in Piura in 2019.

### 3.3 Genetic distance estimate

The sequences corresponding to lineages A. D. (ON1) obtained in this study showed ≥ 96% nucleotide identity and ≥ 90% amino acid identity with respect to the prototype strain ON67-1210A. Among them, the temporally earliest sequence, detected in Peru in May 2009, showed 99% nucleotide and amino acid identity relative to the Ontario isolate from December 2010. Similarly, the sequences assigned to lineages A.3.1/A.3.1.1 (NA1) showed ≥ 96% nucleotide identity and ≥ 98% amino acid identity with respect to the prototype strain NG-016-04.

Pairwise genetic distance analysis showed that the divergent cluster identified in this study (A. D.1*) had its lowest distance to lineage A. D.1, with a *p*-distance value of 0.0179. In comparison, higher levels of divergence were observed relative to the other annotated ON1 lineages, including A. D. (0.0325), A. D.2 (0.0361), A. D.3 (0.0420), A. D.4 (0.0334), and A. D.5 (0.0320) (Table 3).

**Table 3:** Pair distance (*p*-distance) of HRSV-A’s ON1 genotype.

p-distance	[A. D]	[A. D.1]	[A. D.1*]	[A. D.2]	[A. D.3]	[A. D.4]	[A. D.5]
[A. D]		*0.0067*	*0.0093*	*0.0040*	*0.0061*	*0.0075*	*0.0080*
[A. D.1]	0.0198		*0.0066*	*0.0072*	*0.0081*	*0.0063*	*0.0060*
[A. D.1*]	0.0325	0.0179		*0.0096*	*0.0098*	*0.0089*	*0.0087*
[A. D.2]	0.0156	0.0234	0.0361		*0.0068*	*0.0080*	*0.0084*
[A. D.3]	0.0195	0.0304	0.0420	0.0259		*0.0091*	*0.0094*
[A. D.4]	0.0260	0.0212	0.0334	0.0296	0.0387		*0.0056*
[A. D.5]	0.0267	0.0196	0.0320	0.0312	0.0397	0.0192	

Genetic divergence of partial gene G was estimated using the p-distance method (number of nucleotide differences/total sequence length), with a 4-parameter gamma distribution with invariant sites (G4 + I) and 1,000 bootstrap replicates to estimate the standard deviation in MEGA. Values under the diagonal line indicate the number of base differences per site. Values above the diagonal line indicate the estimated standard error.

Among the annotated lineages, pairwise genetic distances ranged from 0.0156 to 0.0397. In this context, the lower distance observed between A. D.1* and A. D.1 supports the close relationship of this cluster with that lineage, whereas its greater divergence relative to the other annotated ON1 lineages is consistent with a divergent cluster nested within A. D.1.

### 3.4 Deduced amino acid sequence analysis

Amino acid sequences of 87 and 111 aa corresponding to the second hypervariable region of the HRSV-A G protein were obtained and were associated with lineages A.3.1/A.3.1.1 and A. D-related lineages, historically included within the NA1 and ON1 genotypes, respectively. Amino acid changes within the lineage A. D. were analyzed relative to the Canadian prototype strain ON67-1210A (GenBank accession number: JN257693), and the sequences detected in this study showed amino acid profiles consistent with the phylogeny obtained.

**Figure 2:**
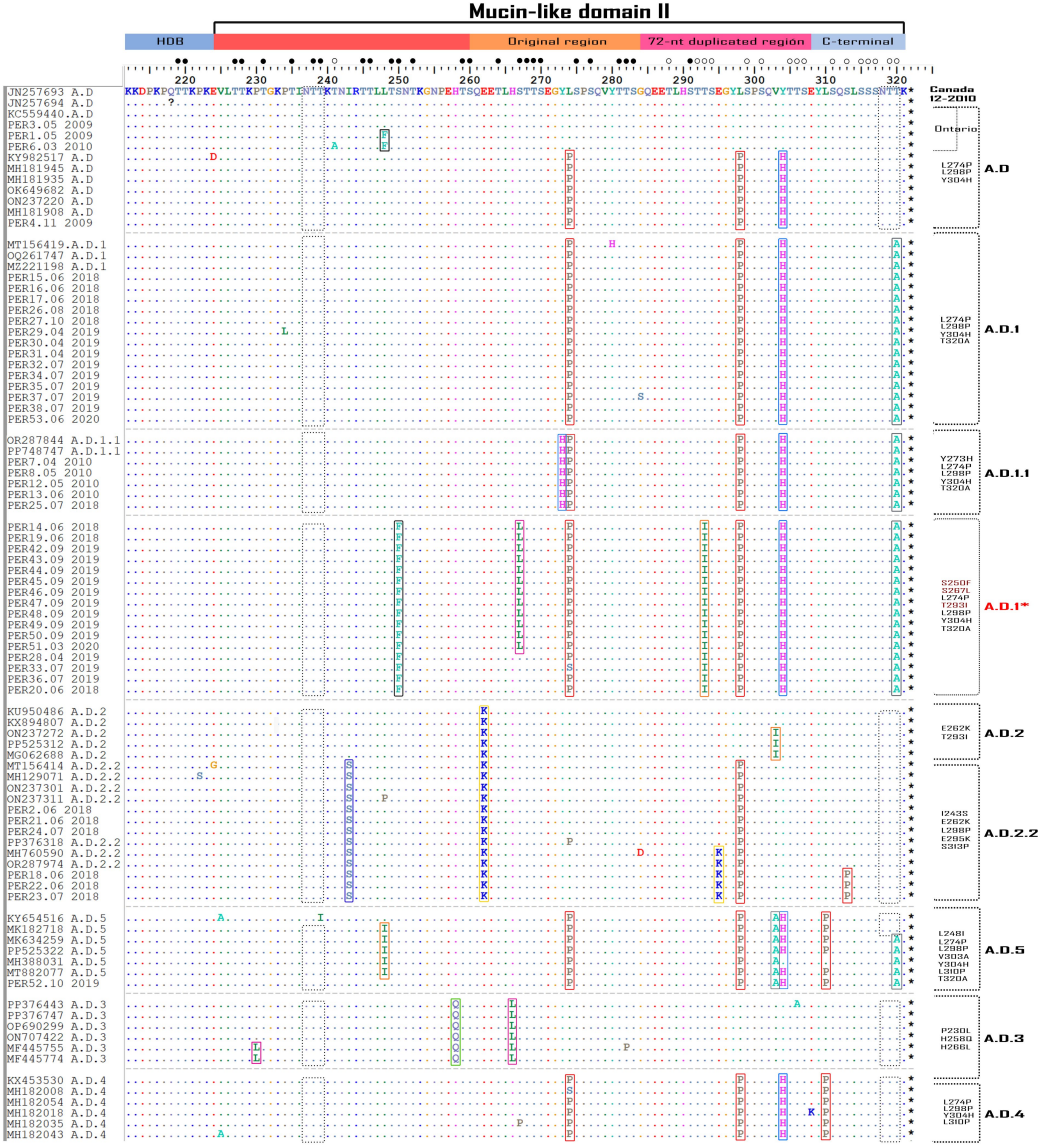
Amino acid profile of positions 212–321 of the second hypervariable region of the HRSV-A G protein, corresponding to the sequences grouped within the A. D. (ON1) lineages detected in this study. Compared with the prototype strain ON67-1210 (GenBank accession number: JN257693), all sequences included show the characteristic 24-aa duplication between positions 285 and 308. Asterisks at the end of each sequence indicate stop codons. Circles above the amino acid position ruler indicate potential O-glycosylation sites in the reference strain; bold circles correspond to positions with a glycosylation potential G ≥ 0.5. Potential N-glycosylation sites are indicated by black dashed rectangles. Colored boxes group sequences that share specific amino acid substitutions. Labels on the right indicate the characteristic mutations observed in each annotated lineage.

The group corresponding to lineage A. D. (*n* = 3) was identical to the prototype strain, except for the substitution L248F, which was observed in two isolates and had not been previously reported. The group corresponding to lineage A. D.1 showed four distinctive substitutions, L274P, L298P, Y304H, and T320A, which had previously been described as ON1:2 in Canada (GenBank accession number: KP321982), Germany (GenBank accession number: MZ397667), and Italy (GenBank accession number: MT156419) (Duvvuri et al., 2015; Tabatabai et al., 2022; Midulla et al., 2020). The group corresponding to lineage A. D.2.2 showed five characteristic substitutions, I243S, E262K, E295K, L298P, and S313P, previously described as ON1:3 in Spain (GenBank accession number: MH129071), Italy (GenBank accession number: MT156414), and Australia (GenBank accession number: MH760624) (Midulla et al., 2020; Gimferrer et al., 2019; Di Giallonardo et al., 2018), except for S313P, which had not been previously reported (Figure 2).

Lineage A. D.5 (*n* = 1) shared the substitutions L274P, L298P, and Y304H with other A. D-related sequences and additionally showed distinctive substitutions such as V303A and L310P; the latter had already been described as ON1:4 in Kenya (GenBank accession number: KX453530) and Germany (GenBank accession number: KJ710405) (Otieno et al., 2017; Tabatabai et al., 2014). No sequences corresponding to group A. D.3 (GenBank accession number: MF445755), previously described in China as ON1:5 (Zhao et al., 2022), or to lineage A. D.4 were detected among the isolates analyzed.

The divergent cluster designated here as A. D.1* showed seven nonsynonymous substitutions, four of which were shared with A. D.1, whereas S250F, S267L, and T293I represented distinctive changes not observed in the other groups analyzed. The substitution T293I was located within the duplicated 24-amino-acid region. In turn, lineage A. D.1.1 showed an amino acid profile similar to that of A. D.1, but with the additional substitution Y273H, which distinguished it (Figure 2).

**Figure 3:**
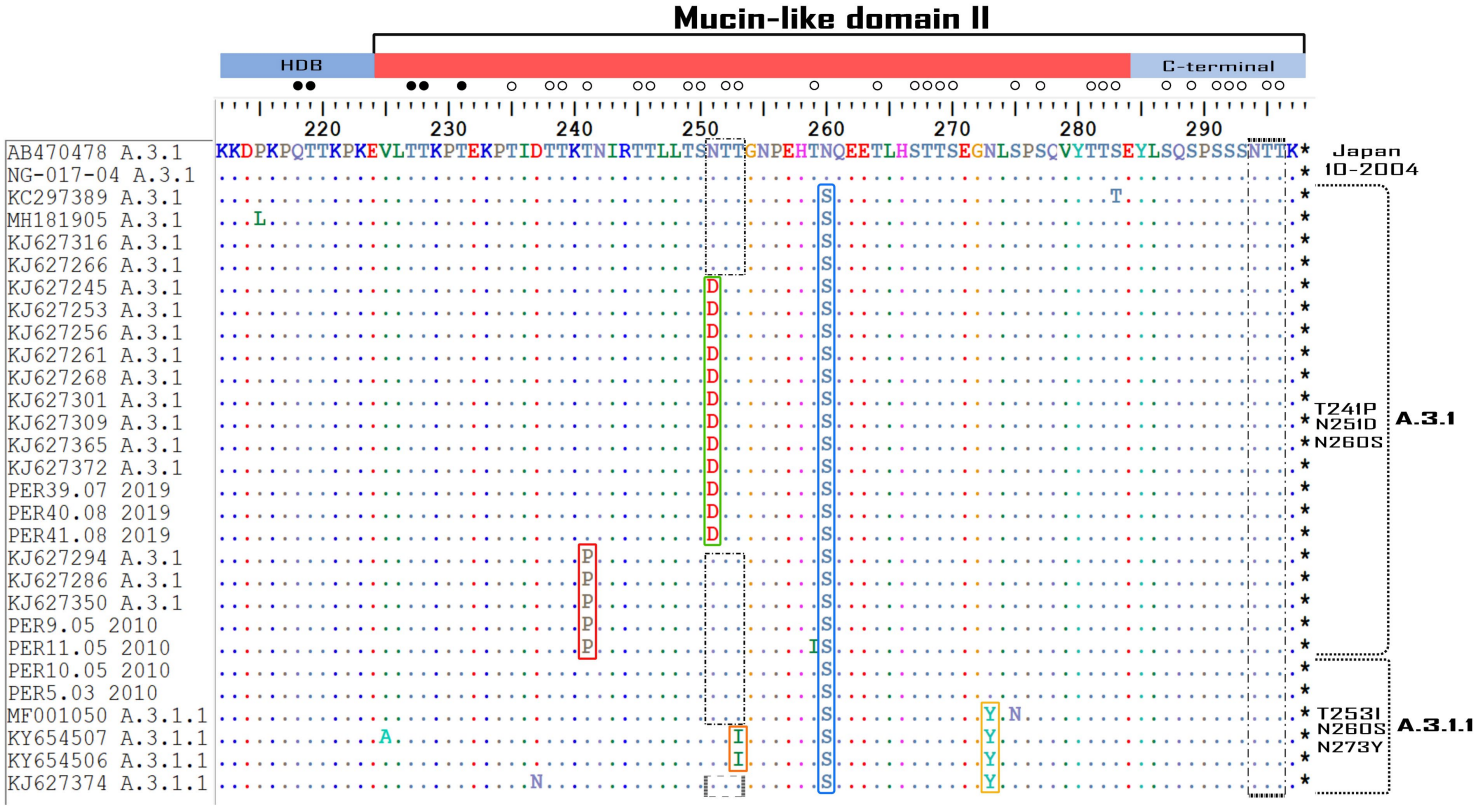
Amino acid profile of positions 212–297 of the second hypervariable region of the HRSV-A G protein, corresponding to the sequences grouped within lineage A.3.1/A.3.1.1 (NA1) detected in this study, in comparison with the prototype strain NG-016-04 (GenBank accession number: AB470478). Asterisks at the end of each sequence indicate stop codons. Circles above the amino acid position ruler indicate potential O-glycosylation sites in the reference strain; bold circles correspond to positions with a glycosylation potential G ≥ 0.5. Potential N-glycosylation sites are indicated by black dashed rectangles. Colored boxes group sequences that share specific amino acid substitutions. Labels on the right indicate the characteristic mutations observed in each annotated lineage.

Amino acid changes within lineages A.3.1/A.3.1.1 were analyzed relative to the Japan prototype strain NG-016-04 (GenBank accession number: AB470478) (Shobugawa et al., 2009), and the sequences detected in this study showed amino acid profiles consistent with the phylogeny obtained. The group assigned to lineage A.3.1 was characterized by the substitutions T241P, N251D, and N260S, whereas the group assigned to lineage A.3.1.1 showed the substitutions T253I, N260S, and N273Y. Notably, the A.3.1 sequences detected in 2009 carried the substitution T241P, whereas those detected in 2010 were characterized by the substitution N251D (Figure 3).

### 3.5 Identification of glycosylation sites

Along the 111 amino acids of the second mucin-like domain of the ON1 isolates, between 1 and 2 new potential N-glycosylation sites with G values ≥ 0.5 were identified (Figure 2). These sites were 237 N and 318 N in the A. D, A. D.2, A. D.2.2, A. D.3, and A. D.4 lineages and only 237 N in the A. D.1, A. D.1*, A. D.1.1, and A. D.5 lineages as a consequence of the T320A substitution. Moreover, the analysis of the probable O-glycosylation sites within the ON1 genotype revealed that they are very variable in number and position between lineages and among them. A. D showed 43 ± 1 probable sites, but only 26 to 28 had glycosylation potential. A. D.1 also had 43 ± 1 probable sites, but only 33 ± 1 could O-glycosylate. A. D.2.2, for instance, could have between 43 and 45 probable sites, but 36 to 40 sites had O-glycosylation potential; the same occurs for ON1 (Buchan et al., 2023; Falsey et al., 2005; Jain et al., 2023; Kaler et al., 2023), even though it has unique mutations. A. D.1* was observed to have 39 ± 1 probable sites, and 26 ± 1 sites had an O-glycosylation potential of G ≥ 0.5 and were the lineage with the fewest serines and threonines that were capable to O-glycosylate. As per the A. D.1.1 group, the Y273H mutation did not alter the number of N- and O-glycosylation sites that it shared with A. D.1 (Supplementary Table S5).

Two sites with N-glycosylation potential (G ≥ 0.5) (Figure 3), which preserve the N251 y N294 positions regarding the Japanese prototype NG-016-04, were identified in the NA1 polypeptides of the strains that were isolated in 2010; however, as per those that were isolated in 2019, only one N-glycosylated site N294, was identified, the N251 being lost as a consequence of the N251D mutation. The number of probable O-glycosylation sites varied from 32 to 34 in the A.3.1/A.3.1.1 isolates, but only the T219, T220, T227, T228, and T231 positions were found to be O-glycosylated, and each one had a score of G ≥ 0.5 (Supplementary Table S6).

### 3.6 tMRCA of ON1 sequences analyzed in this study

**Figure 4:**
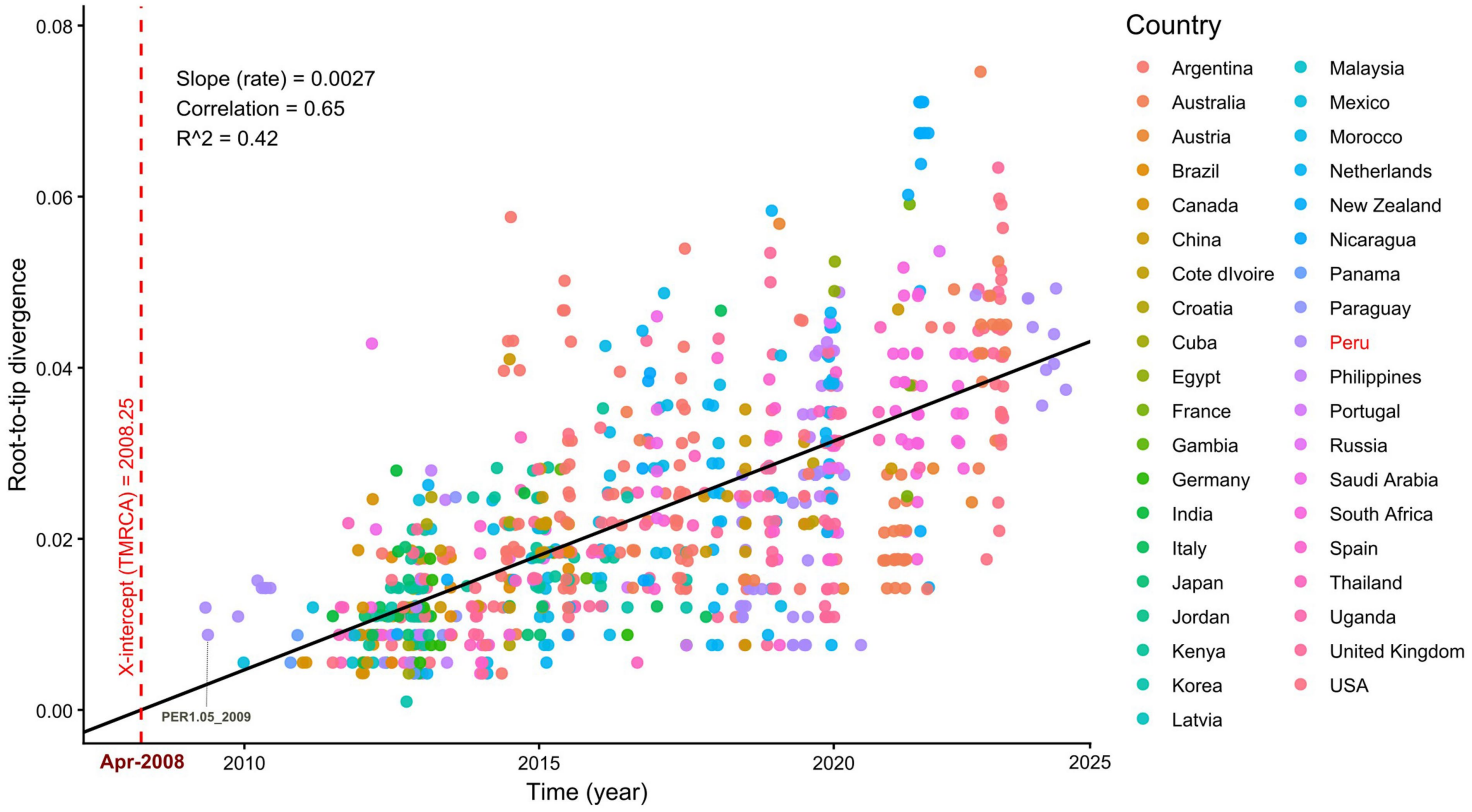
Root-to-tip regression of the HRSV-A A. D (ON1) sequences included in the temporal analysis. Each point represents an individual sequence and is colored according to its country of origin, as indicated in the legend. The black line indicates the linear regression between root-to-tip divergence and sampling year. The red dashed line marks the *x*-intercept, corresponding to an exploratory tMRCA estimate around April 2008. The regression slope was 0.0027 substitutions per site per year, with a correlation of 0.65 and a coefficient of determination *R*^2^ = 0.42. Sequence PER1.05_2009 is highlighted as one of the earliest sequences included in this study.

**Figure 5:**
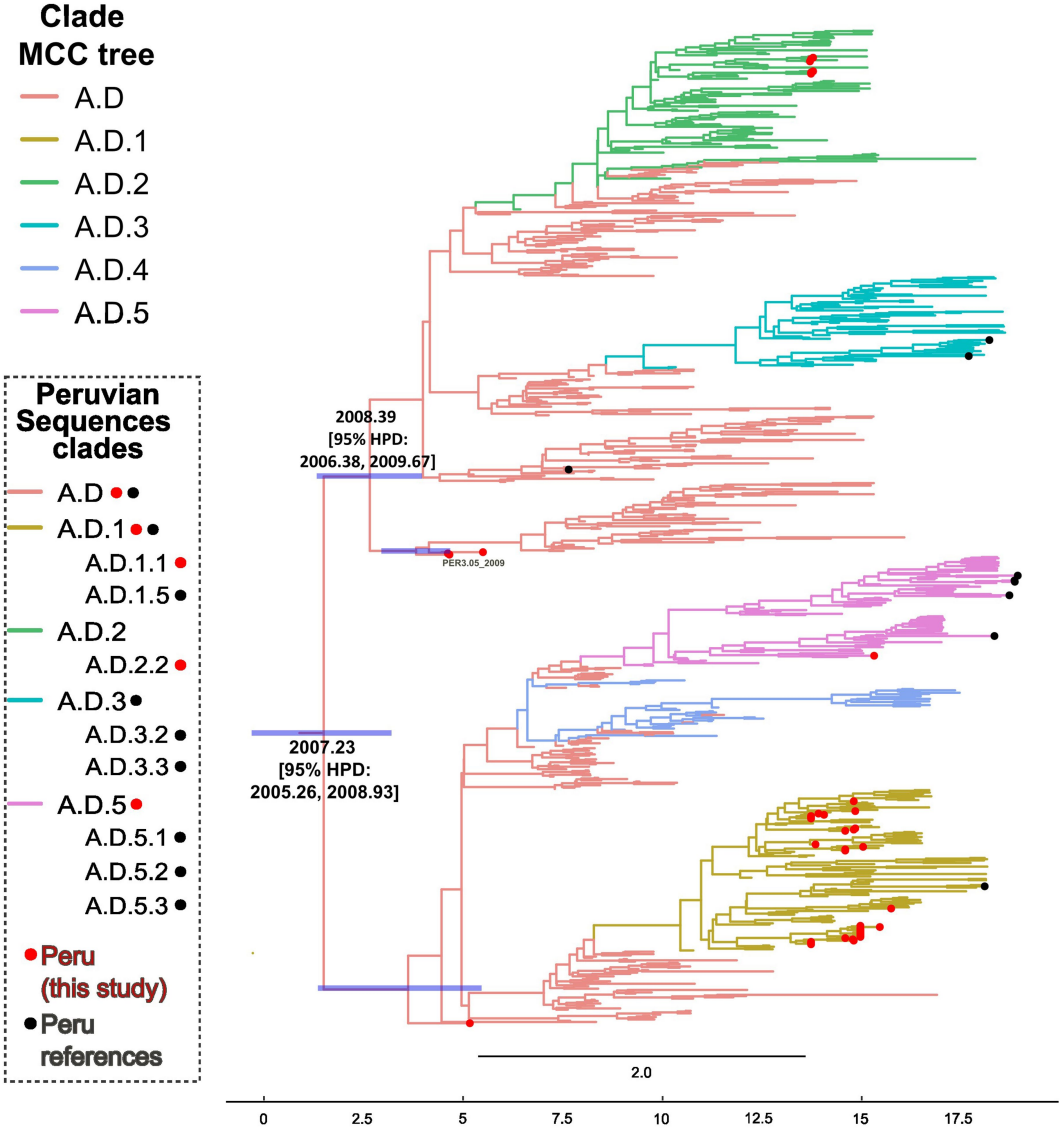
Maximum clade credibility (MCC) tree of the second hypervariable region of the G gene of the A. D (ON1) lineages, reconstructed using Bayesian Markov chain Monte Carlo (MCMC) inference and summarized using median heights. The tree shows the five major clades of the A. D lineages, colored according to the legend. Circles on the branches correspond to sequences obtained in Peru; red circles represent sequences generated in this study, whereas black circles represent annotated Peruvian sequences retrieved from public databases. The dashed box shows exclusively the lineages and nested sublineages reported in Peru. The root node indicates the estimated tMRCA for the entire A. D lineage, whereas the upper node shows the tMRCA of the oldest A. D sequences from Peru. Bars on the nodes represent the 95% highest posterior density (95% HPD) intervals, and the black bar below the tree indicates the temporal scale.

Root-to-tip regression analysis yielded an estimated mean evolutionary rate of 2.70 × 10^−3^ substitutions per site per year for the C-terminal third of the G gene and an exploratory tMRCA estimate around April 2008 (2008.25), with a positive temporal signal (*R*^2^ = 0.42; Figure 4). Similarly, Bayesian MCMC analysis with time scaling estimated a mean tMRCA for the ON1 genotype root at 2007.23 (95% HPD: 2005.26–2008.93) and a mean evolutionary rate of 4.59 × 10^−3^ substitutions/site/year (95% HPD: 4.04 × 10^−3^–5.22 × 10^−3^) for the analyzed fragment of the G gene. In particular, the earliest Peruvian ON1-related sequences showed an estimated mean tMRCA at 2008.39 [95% HPD: 2006.38, 2009.67] (Figure 5). Taken together, these results are consistent with a moderate temporal estimate placing the tMRCA of the ON1 genotype approximately 1 year before the oldest isolates included in this study, PER1.05_2009 and PER3.05_2009.

## 4 Discussion

In this series of hospitalized pediatric patients with acute respiratory infection from the Peruvian coast between 2009 and 2020, HRSV was detected in 20.13% of samples, and 61.88% of HRSV-positive cases corresponded to HRSV-A. These findings confirm that HRSV-A contributed substantially to the burden of severe respiratory infection in the study population, in agreement with the recognized clinical and epidemiological relevance of this virus at both the global and regional levels (Kaler et al., 2023; Rios Guzman and Hultquist, 2022; Ramírez-Soto et al., 2022; del Valle Mendoza et al., 2015; Hönemann et al., 2023; Sovero et al., 2011; Staadegaard et al., 2021). However, our results should be interpreted strictly within the context of a hospitalized pediatric cohort and do not allow associations between viral subtype and age, comorbidities, or clinical severity, as the study was not designed for such analyses. Similarly, the proportion of HRSV-A varied across years, with marked predominance in 2009–2011 and a lower relative frequency from 2018 onward, which is also consistent with reports showing temporal oscillations in subgroup dominance and alternation with HRSV-B according to epidemiological context and season (del Valle Mendoza et al., 2015; Becerra et al., 2019; Korsun et al., 2024).

The identification of clades assigned to A.3.1/A.3.1.1 and A. D./A. D.1/A. D.1.1/A. D.2.2/A. D.5 demonstrates that HRSV-A circulated on the Peruvian coast with genetic diversity and with the co-circulation of clades related to the historically designated NA1 and ON1 genotypes during part of the study period. This pattern is consistent with the global dynamics of the virus and with the progressive replacement of previous variants by ON1 after the emergence of the 72-nucleotide duplication (Duvvuri et al., 2015; Muñoz-Escalante et al., 2019; Goya et al., 2020; Goya et al., 2024; Duvvuri et al., 2015). In addition, our findings are consistent with studies describing circulation in Latin America. Sovero et al. documented the circulation of multiple HRSV-A genotypes in Central and South America, including NA1 and strains related to GA2/GA5, already reflecting substantial regional diversity (Sovero et al., 2011). Subsequently, Duvvuri et al. showed that ON1 spread rapidly worldwide through multiple introductions and sustained local transmission (Duvvuri et al., 2015; Duvvuri et al., 2015). In this context, the detection in Peru of A. D and A. D.1 clades related to ON1, together with A.3.1/A.3.1.1, reinforces the idea that the Peruvian coast was part of the same process of replacement and co-circulation observed in other countries across the region and globally (Midulla et al., 2020; Gimferrer et al., 2019; Di Giallonardo et al., 2018; Otieno et al., 2017; Tabatabai et al., 2014; Zhao et al., 2022; Shobugawa et al., 2009).

A particularly relevant finding was the identification of a monophyletic subclade within A. D.1, here operationally designated A. D.1*, comprising sequences detected in Lima and Piura between 2018 and 2020 and characterized by three exclusive substitutions in the analyzed region of the G protein (S250F, S267L, and T293I). Because this study was based solely on the second hypervariable region of the G gene, these results should be interpreted as evidence of a locally detected divergent cluster rather than as a sufficient basis to formally propose a new lineage or sublineage, in agreement with recent HRSV-A phylogenetic classification frameworks that prioritize the complete G ectodomain or whole genomes (Muñoz-Escalante et al., 2019; Goya et al., 2020; Goya et al., 2024). Nevertheless, the monophyletic clustering with high bootstrap support, its low genetic distance to A. D.1, and the presence of distinctive amino acid changes suggest local ON1 diversification on the Peruvian coast (Duvvuri et al., 2015; Pangesti et al., 2023; LaTourrette and Garcia-Ruiz, 2022; Muñoz-Escalante et al., 2019). Similar patterns have been described in other geographic settings, where ON1 has shown a rapid capacity for diversification and establishment of regional variants (Kim et al., 2023; Duvvuri et al., 2015).

The analysis of potential glycosylation sites provided an initial functional approximation to the differences observed among clades. In ON1-related sequences, the loss of the N318 site in A. D.1, A. D.1.1, A. D.1*, and A. D.5 as a consequence of the T320A substitution, together with the lower number of potentially O-glycosylated sites in A. D.1*, suggests changes that could modify antigenic exposure or the local structure of the G protein (Anderson et al., 2024; McLellan et al., 2013; Eshaghi et al., 2012; Duvvuri et al., 2015; Steentoft et al., 2013; Julenius et al., 2005). Previous studies have shown that the characteristic ON1 duplication and the accumulation of substitutions in the mucin-like region of the G gene are accompanied by variation in O- and N-glycosylation patterns, with possible implications for antigenicity, host interaction, and adaptive advantage (Duvvuri et al., 2015; Midulla et al., 2020; Gimferrer et al., 2019; Di Giallonardo et al., 2018; Otieno et al., 2017; Tabatabai et al., 2014; Zhao et al., 2022; Teirlinck et al., 2021; Feng et al., 2022; Kim et al., 2014). In particular, the global expansion of ON1 has been interpreted as being compatible with increased biological fitness and possible modulation of immune recognition (Eshaghi et al., 2012; Duvvuri et al., 2015; Fonseca et al., 2018). However, in our study, these implications remain inferential, since in silico glycosylation prediction cannot replace experimental validation; therefore, the observed changes should be considered biologically plausible hypotheses that require phenotypic confirmation.

Finally, temporal analysis indicated that the ON1 sequences included in this study are consistent with diversification before 2010 and with an evolutionary rate on the order of 10^−3^ substitutions per site per year in the analyzed region of the G gene, in agreement with previous estimates for HRSV-A and ON1 in particular (Piñana et al., 2024; Eshaghi et al., 2012; Duvvuri et al., 2015; Duvvuri et al., 2015; Teirlinck et al., 2021). The detection of Peruvian sequences from May 2009 related to ON1 is an epidemiologically important observation and extends the known chronology of circulation of this group in South America. However, because the analysis is restricted to a partial G gene fragment and to temporally and geographically unbalanced sampling, these data do not support strong inference regarding the geographic origin of the genotype or the reconstruction of continental dispersal routes (Muñoz-Escalante et al., 2019; Goya et al., 2020; Goya et al., 2024). Rather, our results support a scenario of early circulation and local diversification compatible with the global expansion of ON1 described in other continents (Duvvuri et al., 2015; Song et al., 2017). Taken together, these findings highlight the value of continuous molecular surveillance and the need for genome-based studies to define with greater resolution the evolutionary history of HRSV-A in Peru.

## 5 Conclusion

The objective of the study was achieved by molecularly characterizing representative HRSV-A strains circulating on the Peruvian coast between 2009 and 2020. Our findings show that HRSV-A displayed sustained genetic diversity, with co-circulation of clades related to NA1 and ON1 and subsequent predominance of ON1-related clades. In addition, the detection of a divergent cluster within A. D.1, carrying exclusive substitutions and differences in potential glycosylation sites, suggests local diversification within this viral group. Although the analysis was restricted to the second hypervariable region of the G gene and therefore does not support formal designation of novel lineages or inference of the geographic origin of the genotype, it does provide relevant evidence of early circulation and genetic variability of HRSV-A in Peru. Taken together, these findings expand current understanding of RSV molecular epidemiology in South America and support the need to strengthen genomic surveillance using broader genomic regions or whole-genome sequencing.

## 6 Limitations

This study has limitations. The analyses were based on partial sequencing of the second hypervariable region of the HRSV-A G gene, rather than the complete G ectodomain or whole genomes, which limits phylogenetic resolution and precludes the formal designation of possible new lineages or sublineages. In addition, the inclusion of samples with higher relative viral load, the heterogeneous sampling across years and regions, and the limited availability of HRSV-A sequences from Peru may have introduced sampling bias and underrepresentation of part of the circulating diversity. Similarly, the partial fragment analyzed restricted the temporal analysis to an approximate estimate of the tMRCA of the earliest ON1-related sequences identified in this study and, therefore, should not be interpreted as evidence of phylogeographic spread, dispersal routes, or changes in viral population dynamics. Finally, predictions of glycosylation and potential antigenic effects are in silico estimates and require experimental confirmation.

## Data Availability

The datasets presented in this study can be found in online repositories. The names of the repository/repositories and accession number(s) can be found in the article/Supplementary material.

## Footnotes

1
https://pathoplexus.org/

2
https://gisaid.org

## References

[ref1] Anderson J. Do L. A. H. Van Kasteren P. B. Licciardi P. V. (2024). The role of respiratory syncytial virus G protein in immune cell infection and pathogenesis. EBioMedicine 107:105318. doi: 10.1016/j.ebiom.2024.105318, 39217853 PMC11402919

[ref2] Becerra M. Fiestas V. Tantaleán J. Mallma G. Alvarado M. Gutiérrez V. . (2019). Etiología viral de las infecciones respiratorias agudas graves en una unidad de cuidados intensivos pediátricos [Viral etiology of severe acute respiratory infections in a pediatric intensive care unit]. Rev. Peru Med. Exp. Salud Publica 36, 231–238 Spanish. doi: 10.17843/rpmesp.2019.362.4081 31460634

[ref3] Bender W. Zhang Y. Corbett A. Chu C. Grier A. Wang L. . (2024). Association of disease severity and genetic variation during primary respiratory syncytial virus infections. BMC Med. Genet. 17:165. doi: 10.1186/s12920-024-01930-7, 38898440 PMC11188216

[ref4] Buchan S. A. Chung H. To T. Daneman N. Guttmann A. Kwong J. C. . (2023). Estimating the incidence of first RSV hospitalization in children born in Ontario, Canada. J. Pediatr. Infect. Dis. Soc. 12, 421–430. doi: 10.1093/jpids/piad045, 37335754 PMC10389057

[ref5] Darriba D. Taboada G. Doallo R. Taboada G. L. Posada D. (2012). jModelTest 2: more models, new heuristics and parallel computing. Nat. Methods 9:772. doi: 10.1038/nmeth.2109, 22847109 PMC4594756

[ref6] Del Valle Mendoza J. Cornejo-Tapia A. Weilg P. Verne E. Nazario-Fuertes R. Ugarte C. . (2015). Incidence of respiratory viruses in Peruvian children with acute respiratory infections. J. Med. Virol. 87, 917–924. doi: 10.1002/jmv.24159, 25784285 PMC7167149

[ref7] Di Giallonardo F. Kok J. Fernandez M. Carter I. Geoghegan J. L. Dwyer D. E. . (2018). Evolution of human respiratory syncytial virus (RSV) over multiple seasons in New South Wales, Australia. Viruses 10:476. doi: 10.3390/v10090476, 30200580 PMC6164696

[ref8] Duvvuri V. R. Granados A. Rosenfeld P. Bahl J. Eshaghi A. Gubbay J. B. (2015). Genetic diversity and evolutionary insights of respiratory syncytial virus a ON1 genotype: global and local transmission dynamics. Sci. Rep. 5:14268. doi: 10.1038/srep14268, 26420660 PMC4588507

[ref9] Eshaghi A. Duvvuri V. R. Lai R. Nadarajah J. T. Li A. Patel S. N. . (2012). Genetic variability of human respiratory syncytial virus a strains circulating in Ontario: a novel genotype with a 72 nucleotide G gene duplication. PLoS One 7:e32807. doi: 10.1371/journal.pone.0032807, 22470426 PMC3314658

[ref10] Falsey A. R. Hennessey P. A. Formica M. A. Cox C. Walsh E. E. (2005). Respiratory syncytial virus infection in elderly and high-risk adults. N. Engl. J. Med. 352, 1749–1759. doi: 10.1056/nejmoa043951, 15858184

[ref11] Feng T. Zhang J. Chen Z. Pan W. Chen Z. Yan Y. . (2022). Glycosylation of viral proteins: implication in virus-host interaction and virulence. Virulence 13, 670–683. doi: 10.1080/21505594.2022.2060464, 35436420 PMC9037552

[ref12] Fonseca W. Lukacs N. W. Ptaschinski C. (2018). Factors affecting the immunity to respiratory syncytial virus: from epigenetics to microbiome. Front. Immunol. 9:226. doi: 10.3389/fimmu.2018.00226, 29515570 PMC5825926

[ref13] Gimferrer L. Vila J. Piñana M. Andrés C. Rodrigo-Pendás J. A. Peremiquel-Trillas P. . (2019). Virological surveillance of human respiratory syncytial virus a and B at a tertiary hospital in Catalonia (Spain) during five consecutive seasons (2013-2018). Future Microbiol. 14, 373–381. doi: 10.2217/fmb-2018-0261, 30860397

[ref14] Goya S. Galiano M. Nauwelaers I. Trento A. Openshaw P. J. Mistchenko A. S. . (2020). Toward unified molecular surveillance of RSV: a proposal for genotype definition. Influenza Other Respir. Viruses 14, 274–285. doi: 10.1111/irv.12715, 32022426 PMC7182609

[ref15] Goya S. Ruis C. Neher R. A. Meijer A. Aziz A. Hinrichs A. S. . (2024). Standardized phylogenetic classification of human respiratory syncytial virus below the subgroup level. Emerg. Infect. Dis. 30, 1631–1641. doi: 10.3201/eid3008.240209, 39043393 PMC11286072

[ref16] Guo C. Y. Zhang Y. Zhang Y. Y. Zhao W. Peng X. L. Zheng Y. P. . (2023). Comparative analysis of human respiratory syncytial virus evolutionary patterns during the COVID-19 pandemic and pre-pandemic periods. Front. Microbiol. 14:1298026. doi: 10.3389/fmicb.2023.1298026, 38111642 PMC10725919

[ref17] Gupta R. Brunak S. (2002). Prediction of glycosylation across the human proteome and the correlation to protein function. Pac. Symp. Biocomput. 1, 310–322, 11928486

[ref18] Hönemann M. Thiem S. Bergs S. Berthold T. Propach C. Siekmeyer M. . (2023). In-depth analysis of the re-emergence of respiratory syncytial virus at a tertiary Care Hospital in Germany in the summer of 2021 after the alleviation of non-pharmaceutical interventions due to the SARS-CoV-2 pandemic. Viruses 15:877. doi: 10.3390/v15040877, 37112857 PMC10144477

[ref19] Jafri H. S. Wu X. Makari D. Henrickson K. J. (2013). Distribution of respiratory syncytial virus subtypes a and B among infants presenting to the emergency department with lower respiratory tract infection or apnea. Pediatr. Infect. Dis. J. 32, 335–340. doi: 10.1097/inf.0b013e318282603a, 23337904

[ref20] Jain H. Schweitzer J. W. Justice N. A. (2023). Respiratory Syncytial Virus Infection. Treasure Island, FL: StatPearls Publishing.

[ref21] Julenius K. Mølgaard A. Gupta R. Brunak S. (2005). Prediction, conservation analysis, and structural characterization of mammalian mucin-type O-glycosylation sites. Glycobiology 15, 153–164. doi: 10.1093/glycob/cwh151, 15385431

[ref22] Kaler J. Hussain A. Patel K. Hernandez T. Ray S. (2023). Respiratory syncytial virus: a comprehensive review of transmission, pathophysiology, and manifestation. Cureus 15:e36342. doi: 10.7759/cureus.36342, 37082497 PMC10111061

[ref23] Kim H. N. Hwang J. Yoon S. Y. Lim C. S. Cho Y. Lee C. K. . (2023). Molecular characterization of human respiratory syncytial virus in Seoul, South Korea, during 10 consecutive years, 2010-2019. PLoS One 18:e0283873. doi: 10.1371/journal.pone.0283873, 37023101 PMC10079039

[ref24] Kim Y. J. Kim D. W. Lee W. J. Yun M. R. Lee H. Y. Lee H. S. . (2014). Rapid replacement of human respiratory syncytial virus a with the ON1 genotype having 72 nucleotide duplication in the G gene. Infect. Genet. Evol. 26, 103–112. doi: 10.1016/j.meegid.2014.05.007, 24820343 PMC7106136

[ref25] Korsun N. Trifonova I. Madzharova I. Alexiev I. Uzunova I. Ivanov I. . (2024). Resurgence of respiratory syncytial virus with dominance of RSV-B during the 2022-2023 season. Front. Microbiol. 15:1376389. doi: 10.3389/fmicb.2024.1376389, 38628867 PMC11019023

[ref26] Laham F. R. Mansbach J. M. Piedra P. A. Hasegawa K. Sullivan A. F. Espinola J. A. . (2017). Clinical profiles of respiratory syncytial virus subtypes a and B among children hospitalized with bronchiolitis. Pediatr. Infect. Dis. J. 36:1596. doi: 10.1097/inf.0000000000001596, 28383391 PMC5556381

[ref27] LaTourrette K. Garcia-Ruiz H. (2022). Determinants of virus variation, evolution, and host adaptation. Pathogens 11:1039. doi: 10.3390/pathogens11091039, 36145471 PMC9501407

[ref28] Liu W. Chen D. Tan W. Xu D. Qiu S. Zeng Z. . (2016). Epidemiology and clinical presentations of respiratory syncytial virus subgroups a and B detected with multiplex real-time PCR. PLoS One 11, 1–13. doi: 10.1371/journal.pone.0165108, 27764220 PMC5072546

[ref29] Martin D. P. Murrell B. Golden M. Khoosal A. Muhire B. (2015). RDP4: detection and analysis of recombination patterns in virus genomes. Virus Evol. 1:3. doi: 10.1093/ve/vev003, 27774277 PMC5014473

[ref30] McLellan J. S. Ray W. C. Peeples M. E. (2013). Structure and function of respiratory syncytial virus surface glycoproteins. Curr. Top. Microbiol. Immunol. 372, 83–104. doi: 10.1007/978-3-642-38919-1_4, 24362685 PMC4211642

[ref31] Menardo F. Loiseau C. Brites D. Coscolla M. Gygli S. M. Rutaihwa L. K. . (2018). Treemmer: a tool to reduce large phylogenetic datasets with minimal loss of diversity. BMC Bioinformatics 19:164. doi: 10.1186/s12859-018-2164-8, 29716518 PMC5930393

[ref32] Midulla F. Di Mattia G. Nenna R. Scagnolari C. Viscido A. Oliveto G. . (2020). Novel variants of respiratory syncytial virus a ON1 associated with increased clinical severity of bronchiolitis. J. Infect. Dis. 222, 102–110. doi: 10.1093/infdis/jiaa059, 32031626

[ref33] Minh B. Q. Schmidt H. A. Chernomor O. Schrempf D. Woodhams M. D. von Haeseler A. . (2020). IQ-TREE 2: new models and efficient methods for phylogenetic inference in the genomic era. Mol. Biol. Evol. 37, 1530–1534. doi: 10.1093/molbev/msaa015, 32011700 PMC7182206

[ref34] Muñoz-Escalante J. C. Comas-García A. Bernal-Silva S. Robles-Espinoza C. D. Gómez-Leal G. Noyola D. E. (2019). Respiratory syncytial virus a genotype classification based on systematic intergenotypic and intragenotypic sequence analysis. Sci. Rep. 9:20097. doi: 10.1038/s41598-019-56552-2, 31882808 PMC6934736

[ref35] Obando-Pacheco P. Justicia-Grande A. J. Rivero-Calle I. Rodríguez-Tenreiro C. Sly P. Ramilo O. . (2018). Respiratory syncytial virus seasonality: a global overview. J. Infect. Dis. 217, 1356–1364. doi: 10.1093/infdis/jiy056, 29390105

[ref36] Otieno J. R. Kamau E. M. Agoti C. N. Lewa C. Otieno G. Bett A. . (2017). Spread and evolution of respiratory syncytial virus a genotype ON1, coastal Kenya, 2010-2015. Emerg. Infect. Dis. 23, 264–271. doi: 10.3201/eid2302.161149, 28098528 PMC5324789

[ref37] Pan American Health Organization (2021). Interpretation of real-time RT-PCR Results. Washington, DC: PAHO.

[ref38] Pangesti K. N. A. Ansari H. R. Bayoumi A. Kesson A. M. Hill-Cawthorne G. A. Abd El Ghany M. (2023). Genomic characterization of respiratory syncytial virus genotypes circulating in the paediatric population of Sydney, NSW, Australia. Microb. Genom. 9:1095. doi: 10.1099/mgen.0.001095, 37656160 PMC10569731

[ref39] Peret T. C. Hall C. B. Hammond G. W. Piedra P. A. Storch G. A. Sullender W. M. . (2000). Circulation patterns of group a and B human respiratory syncytial virus genotypes in 5 communities in North America. J. Infect. Dis. 181, 1891–1896. doi: 10.1086/315508, 10837167

[ref40] Piñana M. González-Sánchez A. Andrés C. Vila J. Creus-Costa A. Prats-Méndez I. . (2024). Genomic evolution of human respiratory syncytial virus during a decade (2013-2023): bridging the path to monoclonal antibody surveillance. J. Infect. 88:106153. doi: 10.1016/j.jinf.2024.106153, 38588960

[ref41] Rambaut A. (2018). *Figtree v1.4.4*. Institute of Evolutionary Biology, University of Edinburgh. Available online at: https://tree.bio.ed.ac.uk/software/figtree/.

[ref42] Rambaut A. Lam T. T. Max Carvalho L. Pybus O. G. (2016). Exploring the temporal structure of heterochronous sequences using TempEst (formerly path-O-gen). Virus Evol. 2:7. doi: 10.1093/ve/vew007, 27774300 PMC4989882

[ref43] Ramírez-Soto M. C. Ortega-Cáceres G. Garay-Uribe J. (2022). Characteristics of respiratory syncytial virus versus influenza infection in hospitalized patients of Peru: a retrospective observational study. Trop. Med. Infect. Dis. 7:317. doi: 10.3390/tropicalmed7100317, 36288058 PMC9612014

[ref44] Reiche J. Schweiger B. (2009). Genetic variability of group a human respiratory syncytial virus strains circulating in Germany from 1998 to 2007. J. Clin. Microbiol. 47, 1800–1810. doi: 10.1128/JCM.02286-08, 19386848 PMC2691087

[ref45] Rios Guzman E. Hultquist J. F. (2022). Clinical and biological consequences of respiratory syncytial virus genetic diversity. Ther. Adv. Infect. Dis. 9:128091. doi: 10.1177/20499361221128091, 36225856 PMC9549189

[ref46] Shen W. Sipos B. Zhao L. (2024). SeqKit2: a Swiss army knife for sequence and alignment processing. iMeta 3:e191. doi: 10.1002/imt2.191, 38898985 PMC11183193

[ref47] Shobugawa Y. Saito R. Sano Y. Zaraket H. Suzuki Y. Kumaki A. . (2009). Emerging genotypes of human respiratory syncytial virus subgroup a among patients in Japan. J. Clin. Microbiol. 47, 2475–2482. doi: 10.1128/JCM.00115-09, 19553576 PMC2725673

[ref48] Song J. Zhang Y. Wang H. Shi J. Sun L. Zhang X. . (2017). Emergence of ON1 genotype of human respiratory syncytial virus subgroup a in China between 2011 and 2015. Sci. Rep. 7:5501. doi: 10.1038/s41598-017-04824-0, 28710393 PMC5511225

[ref49] Sovero M. Garcia J. Kochel T. Laguna-Torres V. A. Gomez J. Chicaiza W. . (2011). Circulating strains of human respiratory syncytial virus in central and South America. PLoS One 6:e22111. doi: 10.1371/journal.pone.0022111, 21829605 PMC3148217

[ref50] Staadegaard L. Caini S. Wangchuk S. Thapa B. de Almeida W. A. F. Carvalho F. C. . (2021). The global epidemiology of RSV in community and hospitalized care: findings from 15 countries. Open Forum Infect. Dis. 8:ofab159. doi: 10.1093/ofid/ofab159, 34337092 PMC8320297

[ref51] Steentoft C. Vakhrushev S. Y. Joshi H. J. Kong Y. Vester-Christensen M. B. Schjoldager K. T. . (2013). Precision mapping of the human O-GalNAc glycoproteome through SimpleCell technology. EMBO J. 32, 1478–1488. doi: 10.1038/emboj.2013.79, 23584533 PMC3655468

[ref52] Suchard M. A. Lemey P. Baele G. Ayres D. L. Drummond A. J. Rambaut A. (2018). Bayesian phylogenetic and phylodynamic data integration using BEAST 1.10. Virus Evol. 4:vey016. doi: 10.1093/ve/vey016, 29942656 PMC6007674

[ref53] Tabatabai J. Ihling C. M. Rehbein R. M. Schnee S. V. Hoos J. Pfeil J. . (2022). Molecular epidemiology of respiratory syncytial virus in hospitalised children in Heidelberg, southern Germany, 2014-2017. Infect. Genet. Evol. 98:105209. doi: 10.1016/j.meegid.2022.105209, 35032683

[ref54] Tabatabai J. Prifert C. Pfeil J. Grulich-Henn J. Schnitzler P. (2014). Novel respiratory syncytial virus (RSV) genotype ON1 predominates in Germany during winter season 2012-13. PLoS One 9:e109191. doi: 10.1371/journal.pone.0109191, 25290155 PMC4188618

[ref55] Tamura K. Stecher G. Kumar S. (2021). MEGA11: molecular evolutionary genetics analysis version 11. Mol. Biol. Evol. 38, 3022–3027. doi: 10.1093/molbev/msab120, 33892491 PMC8233496

[ref56] Teirlinck A. C. Broberg E. K. Stuwitz Berg A. Campbell H. Reeves R. M. Carnahan A. . (2021). Recommendations for respiratory syncytial virus surveillance at the national level. Eur. Respir. J. 58:2003766. doi: 10.1183/13993003.03766-2020, 33888523 PMC8485062

[ref57] Vandini S. Biagi C. Lanari M. (2017). Respiratory syncytial virus: the influence of serotype and genotype variability on clinical course of infection. Int. J. Mol. Sci. 18:1717. doi: 10.3390/ijms18081717, 28783078 PMC5578107

[ref58] Vila J. Lera E. Peremiquel-Trillas P. Andrés C. Martínez L. Barceló I. . (2023). Increased RSV-A bronchiolitis severity in RSV-infected children admitted to a reference Center in Catalonia (Spain) between 2014 and 2018. J. Pediatr. Infect. Dis. Soc. 12, 180–183. doi: 10.1093/jpids/piad009, 36744919

[ref59] World Health Organization (2019). WHO Strategy for Global Respiratory Syncytial Virus Surveillance Project Based on the Influenza Platform. Geneva: World Health Organization.

[ref60] Yun K. W. Choi E. H. Lee H. J. (2020). Molecular epidemiology of respiratory syncytial virus for 28 consecutive seasons (1990-2018) and genetic variability of the duplication region in the G gene of genotypes ON1 and BA in South Korea. Arch. Virol. 165, 1069–1077. doi: 10.1007/s00705-020-04580-z, 32144544

[ref61] Zhao X. Wang C. Jiang H. Zhang H. Fang F. Chen M. . (2022). Analysis of circulating respiratory syncytial virus a strains in Shanghai, China, identified a new and increasingly prevalent lineage within the dominant ON1 genotype. Front. Microbiol. 13:6235. doi: 10.3389/fmicb.2022.966235, 36033866 PMC9403419

[ref62] Zlateva K. T. Lemey P. Vandamme A. M. Van Ranst M. (2004). Molecular evolution and circulation patterns of human respiratory syncytial virus subgroup a: positively selected sites in the attachment g glycoprotein. J. Virol. 78, 4675–4683. doi: 10.1128/jvi.78.9.4675-4683.2004, 15078950 PMC387670

