# Supplementary material for: Genetic diversity and molecular characterization of HRSV-A on the coast of Peru, 2009–2020
Source: Front Microbiol. 2026 Jun 10;17:1704867. doi: 10.3389/fmicb.2026.1704867 (PMC13290854; doi:10.3389/fmicb.2026.1704867)
Supplement: Supplementary file 1 [file Data_Sheet_1.docx]

Supplementary Material

# Supplementary Figures and Tables

## Supplementary Tables

**Table S1. List of primers and probes used in this study**

| **Purpose** | **Primer and probes** | **Sequence (5’– 3’)** | **Gene (position)*** | **Fragment(bp)** | **Reference** |
| --- | --- | --- | --- | --- | --- |
| **Real-time RT-PCR primers for RSV detection** | RSV-N15-F | GATGGCTCTTAGCAAAGTCAAGTT | N (15-38) | 169 bp | (Reiche et al., 2009) [29] |
|  | RSV-184-R | CATCTTCWGTGATTAATARCATRCCACATA | N (155-184) |  |  |
|  | RSV-N106- probe | 5`FAM -CTGTCATCYAGCAAATACACYATYCAACGKAGYACAGGAG  -3`TAMRA | N (67-106) |  |  |
| **Real-time RT-PCR primers for RSV-A detection** | RSV-A-F | ACTGCAATCAYACAAGATGCAACRA | G (231-256) | 96 bp | (Liu et al., 2016) [30] |
|  | RSV-A-R | CAGATTGRAGAAGCTGATTCCA | G (306-327) |  |  |
|  | RSV-A-probe | 5`FAM-CCAGATCAAGAACACAACCCCARCATACCT-  3`TAMRA | G (258-287) |  |  |
| **PCR for amplification of the partial G gene of RSV-A** | **PCR** | |  |  | (Reiche et al., 2009) [29] |
|  | RSVA-G513-F | AGTGTTCAACTTTGTACCCTGC | G (513-534) | 583 bp |  |
|  | RSVA-F131-R | CTGCACTGCATGTTGATTGAT | F (111-131) |  |  |
|  | **Nested PCR** | |  |  |  |
|  | RSVA-G606-F | AACCACCACCAAGCCCACAA | G (606–625) | 392 bp |  |
|  | RSV-F22-R | CAACTCCATTGTTATTTGCC | F (3–22) |  |  |
| **Partial sequencing of the RSV-A G gene** | RSVA-G606-F | AACCACCACCAAGCCCACAA | G (606–625) | 392 bp | (Reiche et al., 2009) [29] |
|  | RSV-F22-R | CAACTCCATTGTTATTTGCC | F (3–22) |  |  |

*****Nucleotide positions were annotated according to the gene organization of the prototype HRSV-A genome (GenBank accession number: M74568)

**Table S2. List of HRSV-A G gene reference sequences for phylogenetic analysis**

| **GenBank accession number** | **Label** | **Clade** | **Country** | **Year** |
| --- | --- | --- | --- | --- |
| MG642038 | MG642038_A.1 | A | USA | 1985 |
| MG642060 | MG642060_A.1 | A | USA | 1980 |
| MG642067 | MG642067_A.1 | A | USA | 1987 |
| OK649589 | OK649589_A.1 | A | USA | 1994 |
| OK649668 | OK649668_A | A | USA | 1956 |
| KJ627716 | KJ627716_A.2.1.1 | A.2 | USA | 2003 |
| MG027862 | MG027862_A.2.1.1 | A.2 | USA | 2003 |
| OK649606 | OK649606_A.2.1.1 | A.2 | Brazil | 2008 |
| OK649617 | OK649617_A.2.1.1 | A.2 | Brazil | 2008 |
| OK649635 | OK649635_A.2.1.1 | A.2 | Brazil | 2009 |
| OK649649 | OK649649_A.2.1.1 | A.2 | Brazil | 2010 |
| KJ627652 | KJ627652_A.2.1 | A.2 | USA | 2002 |
| KJ627656 | KJ627656_A.2.1 | A.2 | USA | 2003 |
| KJ627672 | KJ627672_A.2.1 | A.2 | USA | 2001 |
| KJ627685 | KJ627685_A.2.1 | A.2 | USA | 2001 |
| KJ627690 | KJ627690_A.2.1 | A.2 | USA | 2001 |
| KJ627704 | KJ627704_A.2.1 | A.2 | USA | 2001 |
| KJ627695 | KJ627695_A.2 | A.2 | USA | 2001 |
| MG642024 | MG642024_A.2 | A.2 | USA | 1981 |
| MG642052 | MG642052_A.2 | A.2 | USA | 1994 |
| MG642079 | MG642079_A.2 | A.2 | USA | 1982 |
| MG642083 | MG642083_A.2 | A.2 | USA | 1987 |
| KJ627647 | KJ627647_A.3 | A.3 | USA | 2001 |
| MG642026 | MG642026_A.3 | A.3 | USA | 1982 |
| MG642030 | MG642030_A.3 | A.3 | USA | 1988 |
| MG642035 | MG642035_A.3 | A.3 | USA | 1984 |
| MG642063 | MG642063_A.3 | A.3 | USA | 1982 |
| MG642070 | MG642070_A.3 | A.3 | USA | 1986 |
| MG642077 | MG642077_A.3 | A.3 | USA | 1987 |
| OK649642 | OK649642_A.3 | A.3 | Brazil | 2010 |
| OK649678 | OK649678_A.3 | A.3 | USA | 2005 |
| OK649679 | OK649679_A.3 | A.3 | USA | 2005 |
| MF001041 | MF001041_A.3.1 | A.3.1 | USA | 2015 |
| MF001052 | MF001052_A.3.1 | A.3.1 | USA | 2015 |
| MH181878 | MH181878_A.3.1 | A.3.1 | Kenya | 2011 |
| MH181883 | MH181883_A.3.1 | A.3.1 | Kenya | 2012 |
| MH181902 | MH181902_A.3.1 | A.3.1 | Kenya | 2013 |
| MK109763 | MK109763_A.3.1 | A.3.1 | Jordan | 2011 |
| MK109773 | MK109773_A.3.1 | A.3.1 | Jordan | 2011 |
| MK109785 | MK109785_A.3.1 | A.3.1 | Jordan | 2010 |
| OK649607 | OK649607_A.3.1 | A.3.1 | Brazil | 2008 |
| OK649618 | OK649618_A.3.1 | A.3.1 | Brazil | 2008 |
| OK649622 | OK649622_A.3.1 | A.3.1 | Brazil | 2008 |
| OK649624 | OK649624_A.3.1 | A.3.1 | Brazil | 2008 |
| OK649626 | OK649626_A.3.1 | A.3.1 | Brazil | 2008 |
| OK649628 | OK649628_A.3.1 | A.3.1 | Brazil | 2008 |
| OK649630 | OK649630_A.3.1 | A.3.1 | Brazil | 2008 |
| OK649636 | OK649636_A.3.1 | A.3.1 | Brazil | 2009 |
| OK649638 | OK649638_A.3.1 | A.3.1 | Brazil | 2009 |
| OK649641 | OK649641_A.3.1 | A.3.1 | Brazil | 2009 |
| OK649645 | OK649645_A.3.1 | A.3.1 | Brazil | 2010 |
| OK649648 | OK649648_A.3.1 | A.3.1 | Brazil | 2010 |
| OK649667 | OK649667_A.3.1 | A.3.1 | Kenya | 2007 |
| KJ627738 | KJ627738_A.3.1.1 | A.3.1.1 | USA | 2013 |
| KY654506 | KY654506_A.3.1.1 | A.3.1.1 | Philippines | 2012 |
| KY654507 | KY654507_A.3.1.1 | A.3.1.1 | Philippines | 2012 |
| KY654509 | KY654509_A.3.1.1 | A.3.1.1 | Philippines | 2012 |
| MF001043 | MF001043_A.3.1.1 | A.3.1.1 | USA | 2015 |
| MH181899 | MH181899_A.3.1.1 | A.3.1.1 | Kenya | 2013 |
| MH181906 | MH181906_A.3.1.1 | A.3.1.1 | Kenya | 2015 |
| OK649639 | OK649639_A.3.1.1 | A.3.1.1 | Brazil | 2009 |
| OK649680 | OK649680_A.3.1.1 | A.3.1.1 | USA | 2012 |
| ON237257 | ON237257_A.3.1.1 | A.3.1.1 | Argentina | 2015 |
| MH181908 | MH181908_A.D | A.D | Kenya | 2012 |
| MH181932 | MH181932_A.D | A.D | Kenya | 2012 |
| MH181935 | MH181935_A.D | A.D | Kenya | 2012 |
| MH181940 | MH181940_A.D | A.D | Kenya | 2012 |
| MH181945 | MH181945_A.D | A.D | Kenya | 2012 |
| MH181948 | MH181948_A.D | A.D | Kenya | 2012 |
| MH181961 | MH181961_A.D | A.D | Kenya | 2012 |
| MH181969 | MH181969_A.D | A.D | Kenya | 2012 |
| MH181970 | MH181970_A.D | A.D | Kenya | 2012 |
| MH181976 | MH181976_A.D | A.D | Kenya | 2012 |
| MH181982 | MH181982_A.D | A.D | Kenya | 2013 |
| MH182025 | MH182025_A.D | A.D | Kenya | 2014 |
| MH279547 | MH279547_A.D | A.D | China | 2017 |
| MH447953 | MH447953_A.D | A.D | Thailand | 2013 |
| MH447959 | MH447959_A.D | A.D | Thailand | 2017 |
| MN630104 | MN630104_A.D | A.D | USA | 2016 |
| MN630106 | MN630106_A.D | A.D | USA | 2016 |
| MZ221196 | MZ221196_A.D | A.D | China | 2014 |
| MZ221197 | MZ221197_A.D | A.D | China | 2018 |
| MZ221199 | MZ221199_A.D | A.D | China | 2018 |
| MZ221205 | MZ221205_A.D | A.D | China | 2014 |
| MZ515641 | MZ515641_A.D | A.D | Netherlands | 2018 |
| ON237225 | ON237225_A.D | A.D | Argentina | 2014 |
| ON237230 | ON237230_A.D | A.D | Argentina | 2014 |
| ON237232 | ON237232_A.D | A.D | Argentina | 2014 |
| ON237234 | ON237234_A.D | A.D | Argentina | 2014 |
| ON237244 | ON237244_A.D | A.D | Argentina | 2014 |
| ON237245 | ON237245_A.D | A.D | Argentina | 2014 |
| ON237246 | ON237246_A.D | A.D | Argentina | 2015 |
| ON237252 | ON237252_A.D | A.D | Argentina | 2015 |
| ON237259 | ON237259_A.D | A.D | Argentina | 2015 |
| ON237261 | ON237261_A.D | A.D | Argentina | 2015 |
| ON237265 | ON237265_A.D | A.D | Argentina | 2015 |
| ON237269 | ON237269_A.D | A.D | Argentina | 2015 |
| ON237306 | ON237306_A.D | A.D | Argentina | 2017 |
| OQ261746 | OQ261746_A.D | A.D | Austria | 2019 |
| JN257693 | JN257693_A.D | A.D | Canada | 2010 |
| KC559440 | KC559440_A.D | A.D | China | 2012 |
| JN257694 | JN257694_A.D | A.D | Canada | 2011 |
| OP744439 | OP744439_A.D.1 | A.D.1 | Kenya | 2021 |
| OP927830 | OP927830_A.D.1 | A.D.1 | Germany | 2021 |
| OP927848 | OP927848_A.D.1 | A.D.1 | Germany | 2021 |
| OP927874 | OP927874_A.D.1 | A.D.1 | Germany | 2021 |
| OP927894 | OP927894_A.D.1 | A.D.1 | Germany | 2021 |
| OP927933 | OP927933_A.D.1 | A.D.1 | Germany | 2021 |
| OR795415 | OR795415_A.D.1 | A.D.1 | Germany | 2021 |
| OR795441 | OR795441_A.D.1 | A.D.1 | Germany | 2021 |
| OR795454 | OR795454_A.D.1 | A.D.1 | Germany | 2021 |
| OR795455 | OR795455_A.D.1 | A.D.1 | Germany | 2021 |
| OR795468 | OR795468_A.D.1 | A.D.1 | Germany | 2021 |
| PP530274 | PP530274_A.D.1 | A.D.1 | USA | 2023 |
| PQ308736 | PQ308736_A.D.1 | A.D.1 | USA | 2021 |
| PQ348801 | PQ348801_A.D.1 | A.D.1 | United Kingdom | 2021 |
| PQ348848 | PQ348848_A.D.1 | A.D.1 | United Kingdom | 2021 |
| PQ348849 | PQ348849_A.D.1 | A.D.1 | United Kingdom | 2021 |
| PQ348990 | PQ348990_A.D.1 | A.D.1 | United Kingdom | 2021 |
| PQ348996 | PQ348996_A.D.1 | A.D.1 | United Kingdom | 2021 |
| PQ762721 | PQ762721_A.D.1 | A.D.1 | France | 2023 |
| PQ762726 | PQ762726_A.D.1 | A.D.1 | France | 2023 |
| PQ762769 | PQ762769_A.D.1 | A.D.1 | France | 2023 |
| PQ762801 | PQ762801_A.D.1 | A.D.1 | France | 2023 |
| PQ763064 | PQ763064_A.D.1 | A.D.1 | France | 2023 |
| LR699736 | LR699736_A.D.1 | A.D.1 | United Kingdom | 2018 |
| MZ221198 | MZ221198_A.D.1 | A.D.1 | China | 2018 |
| MZ515571 | MZ515571_A.D.1 | A.D.1 | United Kingdom | 2020 |
| MZ515573 | MZ515573_A.D.1 | A.D.1 | United Kingdom | 2020 |
| MZ515583 | MZ515583_A.D.1 | A.D.1 | Spain | 2019 |
| MZ515628 | MZ515628_A.D.1 | A.D.1 | Netherlands | 2019 |
| MZ515645 | MZ515645_A.D.1 | A.D.1 | United Kingdom | 2019 |
| MZ515649 | MZ515649_A.D.1 | A.D.1 | Netherlands | 2019 |
| MZ515688 | MZ515688_A.D.1 | A.D.1 | Spain | 2019 |
| MZ515744 | MZ515744_A.D.1 | A.D.1 | Netherlands | 2019 |
| MZ515803 | MZ515803_A.D.1 | A.D.1 | Netherlands | 2019 |
| ON237314 | ON237314_A.D.1 | A.D.1 | Argentina | 2017 |
| ON237343 | ON237343_A.D.1 | A.D.1 | Argentina | 2017 |
| OQ171909 | OQ171909_A.D.1 | A.D.1 | USA | 2022 |
| OQ171919 | OQ171919_A.D.1 | A.D.1 | USA | 2022 |
| OQ261747 | OQ261747_A.D.1 | A.D.1 | Austria | 2020 |
| OR287947 | OR287947_A.D.1.1 | A.D.1.1 | USA | 2020 |
| PP748722 | PP748722_A.D.1.1 | A.D.1.1 | USA | 2020 |
| OR287844 | OR287844_A.D.1.1 | A.D.1.1 | USA | 2019 |
| OR287923 | OR287923_A.D.1.1 | A.D.1.1 | USA | 2020 |
| PP748747 | PP748747_A.D.1.1 | A.D.1.1 | USA | 2020 |
| OR795414 | OR795414_A.D.1.10 | A.D.1.10 | Germany | 2021 |
| PQ762693 | PQ762693_A.D.1.10 | A.D.1.10 | France | 2023 |
| PQ762701 | PQ762701_A.D.1.10 | A.D.1.10 | France | 2023 |
| PQ762731 | PQ762731_A.D.1.10 | A.D.1.10 | France | 2023 |
| PQ762771 | PQ762771_A.D.1.10 | A.D.1.10 | France | 2023 |
| PQ762947 | PQ762947_A.D.1.10 | A.D.1.10 | France | 2023 |
| PQ763049 | PQ763049_A.D.1.10 | A.D.1.10 | France | 2023 |
| PQ763058 | PQ763058_A.D.1.10 | A.D.1.10 | France | 2023 |
| PQ763110 | PQ763110_A.D.1.10 | A.D.1.10 | France | 2023 |
| OR162227 | OR162227_A.D.1.11 | A.D.1.11 | Kenya | 2022 |
| PP830378 | PP830378_A.D.1.11 | A.D.1.11 | USA | 2023 |
| PP970021 | PP970021_A.D.1.11 | A.D.1.11 | Ireland | 2023 |
| PQ762656 | PQ762656_A.D.1.11 | A.D.1.11 | France | 2023 |
| PQ763129 | PQ763129_A.D.1.11 | A.D.1.11 | France | 2023 |
| OM857213 | OM857213_A.D.1.3 | A.D.1.3 | Australia | 2020 |
| OM857227 | OM857227_A.D.1.3 | A.D.1.3 | Australia | 2020 |
| OM857230 | OM857230_A.D.1.3 | A.D.1.3 | Australia | 2020 |
| OM857236 | OM857236_A.D.1.3 | A.D.1.3 | Australia | 2020 |
| OM857248 | OM857248_A.D.1.3 | A.D.1.3 | Australia | 2020 |
| OM857296 | OM857296_A.D.1.3 | A.D.1.3 | Australia | 2020 |
| OM857323 | OM857323_A.D.1.3 | A.D.1.3 | Australia | 2020 |
| OM857330 | OM857330_A.D.1.3 | A.D.1.3 | Australia | 2020 |
| OM857344 | OM857344_A.D.1.3 | A.D.1.3 | Australia | 2020 |
| OM857346 | OM857346_A.D.1.3 | A.D.1.3 | Australia | 2021 |
| OM857352 | OM857352_A.D.1.3 | A.D.1.3 | Australia | 2021 |
| OM857359 | OM857359_A.D.1.3 | A.D.1.3 | Australia | 2020 |
| OM857362 | OM857362_A.D.1.3 | A.D.1.3 | Australia | 2020 |
| OQ024155 | OQ024155_A.D.1.4 | A.D.1.4 | USA | 2022 |
| OR143188 | OR143188_A.D.1.4 | A.D.1.4 | USA | 2022 |
| OR522496 | OR522496_A.D.1.4 | A.D.1.4 | USA | 2022 |
| OR915735 | OR915735_A.D.1.4 | A.D.1.4 | USA | 2023 |
| OY757595 | OY757595_A.D.1.4 | A.D.1.4 | Australia | 2022 |
| PP681242 | PP681242_A.D.1.4 | A.D.1.4 | USA | 2022 |
| PP681283 | PP681283_A.D.1.4 | A.D.1.4 | USA | 2022 |
| PP770472 | PP770472_A.D.1.4 | A.D.1.4 | USA | 2024 |
| PP970000 | PP970000_A.D.1.4 | A.D.1.4 | Ireland | 2023 |
| PP970032 | PP970032_A.D.1.4 | A.D.1.4 | Ireland | 2023 |
| OQ171927 | OQ171927_A.D.1.5 | A.D.1.5 | USA | 2022 |
| OR522529 | OR522529_A.D.1.5 | A.D.1.5 | USA | 2023 |
| OR872607 | OR872607_A.D.1.5 | A.D.1.5 | USA | 2022 |
| OR882986 | OR882986_A.D.1.5 | A.D.1.5 | USA | 2023 |
| OR882991 | OR882991_A.D.1.5 | A.D.1.5 | USA | 2022 |
| OR883018 | OR883018_A.D.1.5 | A.D.1.5 | USA | 2022 |
| PP342426 | PP342426_A.D.1.5 | A.D.1.5 | USA | 2023 |
| PP530267 | PP530267_A.D.1.5 | A.D.1.5 | USA | 2023 |
| PP668172 | PP668172_A.D.1.5 | A.D.1.5 | USA | 2023 |
| PP781385 | PP781385_A.D.1.5 | A.D.1.5 | USA | 2024 |
| PP781402 | PP781402_A.D.1.5 | A.D.1.5 | USA | 2023 |
| PP781426 | PP781426_A.D.1.5 | A.D.1.5 | USA | 2024 |
| PP790963 | PP790963_A.D.1.5 | A.D.1.5 | Mexico | 2023 |
| PP847391 | PP847391_A.D.1.5 | A.D.1.5 | USA | 2024 |
| PP978501 | PP978501_A.D.1.5 | A.D.1.5 | USA | 2024 |
| PQ390732 | PQ390732_A.D.1.5 | A.D.1.5 | USA | 2024 |
| PQ610205 | PQ610205_A.D.1.5 | A.D.1.5 | Argentina | 2023 |
| PQ610207 | PQ610207_A.D.1.5 | A.D.1.5 | Argentina | 2023 |
| PQ638670 | PQ638670_A.D.1.5 | A.D.1.5 | USA | 2022 |
| PQ762761 | PQ762761_A.D.1.5 | A.D.1.5 | France | 2023 |
| PQ762938 | PQ762938_A.D.1.5 | A.D.1.5 | France | 2023 |
| PQ763004 | PQ763004_A.D.1.5 | A.D.1.5 | France | 2023 |
| PP270250 | PP270250_A.D.1.6 | A.D.1.6 | USA | 2023 |
| PP270262 | PP270262_A.D.1.6 | A.D.1.6 | USA | 2023 |
| PP770476 | PP770476_A.D.1.6 | A.D.1.6 | USA | 2024 |
| PP781409 | PP781409_A.D.1.6 | A.D.1.6 | USA | 2024 |
| PP795160 | PP795160_A.D.1.6 | A.D.1.6 | USA | 2024 |
| PP847369 | PP847369_A.D.1.6 | A.D.1.6 | USA | 2024 |
| PP847386 | PP847386_A.D.1.6 | A.D.1.6 | USA | 2024 |
| PP970036 | PP970036_A.D.1.6 | A.D.1.6 | Ireland | 2023 |
| PP970035 | PP970035_A.D.1.6 | A.D.1.6 | Ireland | 2023 |
| PQ762843 | PQ762843_A.D.1.6 | A.D.1.6 | France | 2024 |
| PQ763086 | PQ763086_A.D.1.6 | A.D.1.6 | France | 2023 |
| PQ763126 | PQ763126_A.D.1.6 | A.D.1.6 | France | 2023 |
| PQ788206 | PQ788206_A.D.1.6 | A.D.1.6 | USA | 2024 |
| OQ024124 | OQ024124_A.D.1.7 | A.D.1.7 | USA | 2022 |
| OQ171904 | OQ171904_A.D.1.7 | A.D.1.7 | USA | 2022 |
| OQ171911 | OQ171911_A.D.1.7 | A.D.1.7 | USA | 2022 |
| OQ171931 | OQ171931_A.D.1.7 | A.D.1.7 | USA | 2022 |
| OQ933803 | OQ933803_A.D.1.7 | A.D.1.7 | China | 2021 |
| OQ933805 | OQ933805_A.D.1.7 | A.D.1.7 | China | 2021 |
| OR143183 | OR143183_A.D.1.7 | A.D.1.7 | USA | 2022 |
| OR493308 | OR493308_A.D.1.7 | A.D.1.7 | Spain | 2022 |
| OR522473 | OR522473_A.D.1.7 | A.D.1.7 | USA | 2022 |
| OR522527 | OR522527_A.D.1.7 | A.D.1.7 | USA | 2023 |
| OR601470 | OR601470_A.D.1.7 | A.D.1.7 | USA | 2022 |
| OR872619 | OR872619_A.D.1.7 | A.D.1.7 | USA | 2022 |
| PP681260 | PP681260_A.D.1.7 | A.D.1.7 | USA | 2022 |
| PP681280 | PP681280_A.D.1.7 | A.D.1.7 | USA | 2023 |
| PP910758 | PP910758_A.D.1.7 | A.D.1.7 | USA | 2024 |
| PP974152 | PP974152_A.D.1.7 | A.D.1.7 | China | 2021 |
| PQ618040 | PQ618040_A.D.1.7 | A.D.1.7 | Panama | 2022 |
| PQ618041 | PQ618041_A.D.1.7 | A.D.1.7 | Panama | 2022 |
| OQ024109 | OQ024109_A.D.1.8 | A.D.1.8 | USA | 2022 |
| OQ171901 | OQ171901_A.D.1.8 | A.D.1.8 | USA | 2022 |
| OR143170 | OR143170_A.D.1.8 | A.D.1.8 | USA | 2022 |
| OR143189 | OR143189_A.D.1.8 | A.D.1.8 | USA | 2023 |
| OR287988 | OR287988_A.D.1.8 | A.D.1.8 | USA | 2021 |
| OR522462 | OR522462_A.D.1.8 | A.D.1.8 | USA | 2022 |
| OR522497 | OR522497_A.D.1.8 | A.D.1.8 | USA | 2022 |
| OR872587 | OR872587_A.D.1.8 | A.D.1.8 | USA | 2022 |
| OR872622 | OR872622_A.D.1.8 | A.D.1.8 | USA | 2022 |
| OR872628 | OR872628_A.D.1.8 | A.D.1.8 | USA | 2022 |
| PP495851 | PP495851_A.D.1.8 | A.D.1.8 | USA | 2024 |
| PP781407 | PP781407_A.D.1.8 | A.D.1.8 | USA | 2024 |
| PQ788250 | PQ788250_A.D.1.8 | A.D.1.8 | USA | 2024 |
| OR162269 | OR162269_A.D.1.9 | A.D.1.9 | Kenya | 2022 |
| PP781415 | PP781415_A.D.1.9 | A.D.1.9 | USA | 2024 |
| PP781416 | PP781416_A.D.1.9 | A.D.1.9 | USA | 2023 |
| PP959045 | PP959045_A.D.1.9 | A.D.1.9 | USA | 2024 |
| PQ066220 | PQ066220_A.D.1.9 | A.D.1.9 | USA | 2023 |
| PQ762730 | PQ762730_A.D.1.9 | A.D.1.9 | France | 2023 |
| PQ762742 | PQ762742_A.D.1.9 | A.D.1.9 | France | 2023 |
| PQ762752 | PQ762752_A.D.1.9 | A.D.1.9 | France | 2023 |
| PQ762816 | PQ762816_A.D.1.9 | A.D.1.9 | France | 2023 |
| PQ762834 | PQ762834_A.D.1.9 | A.D.1.9 | France | 2023 |
| PQ762979 | PQ762979_A.D.1.9 | A.D.1.9 | France | 2023 |
| PQ763041 | PQ763041_A.D.1.9 | A.D.1.9 | France | 2023 |
| PQ763105 | PQ763105_A.D.1.9 | A.D.1.9 | France | 2023 |
| PQ763120 | PQ763120_A.D.1.9 | A.D.1.9 | France | 2024 |
| PQ788216 | PQ788216_A.D.1.9 | A.D.1.9 | USA | 2024 |
| OR466268 | OR466268_A.D.2 | A.D.2 | Zambia | 2022 |
| MK749913 | MK749913_A.D.2 | A.D.2 | Nicaragua | 2016 |
| ON237272 | ON237272_A.D.2 | A.D.2 | Argentina | 2015 |
| LC712535 | LC712535_A.D.2.1 | A.D.2.1 | Japan | 2020 |
| LC712538 | LC712538_A.D.2.1 | A.D.2.1 | Japan | 2020 |
| OR287922 | OR287922_A.D.2.1 | A.D.2.1 | USA | 2020 |
| PP376538 | PP376538_A.D.2.1 | A.D.2.1 | Netherlands | 2020 |
| PP969999 | PP969999_A.D.2.1 | A.D.2.1 | Ireland | 2023 |
| PQ066224 | PQ066224_A.D.2.1 | A.D.2.1 | USA | 2024 |
| PQ762763 | PQ762763_A.D.2.1 | A.D.2.1 | France | 2023 |
| PQ788245 | PQ788245_A.D.2.1 | A.D.2.1 | USA | 2024 |
| MZ515651 | MZ515651_A.D.2.1 | A.D.2.1 | Netherlands | 2019 |
| LC712488 | LC712488_A.D.2.2 | A.D.2.2 | Japan | 2020 |
| OR287962 | OR287962_A.D.2.2 | A.D.2.2 | USA | 2020 |
| OR287974 | OR287974_A.D.2.2 | A.D.2.2 | USA | 2020 |
| OR466295 | OR466295_A.D.2.2 | A.D.2.2 | Zambia | 2022 |
| OR466296 | OR466296_A.D.2.2 | A.D.2.2 | Zambia | 2023 |
| OR466311 | OR466311_A.D.2.2 | A.D.2.2 | Zambia | 2021 |
| OR466312 | OR466312_A.D.2.2 | A.D.2.2 | Zambia | 2022 |
| OR466323 | OR466323_A.D.2.2 | A.D.2.2 | Zambia | 2021 |
| OR795387 | OR795387_A.D.2.2 | A.D.2.2 | Germany | 2020 |
| PP376318 | PP376318_A.D.2.2 | A.D.2.2 | France | 2020 |
| PP376403 | PP376403_A.D.2.2 | A.D.2.2 | Germany | 2020 |
| PP376634 | PP376634_A.D.2.2 | A.D.2.2 | Canada | 2020 |
| PP376659 | PP376659_A.D.2.2 | A.D.2.2 | Finland | 2020 |
| MH383066 | MH383066_A.D.2.2 | A.D.2.2 | Lebanon | 2014 |
| MH447958 | MH447958_A.D.2.2 | A.D.2.2 | Thailand | 2016 |
| MK749867 | MK749867_A.D.2.2 | A.D.2.2 | Nicaragua | 2016 |
| MK749890 | MK749890_A.D.2.2 | A.D.2.2 | Nicaragua | 2016 |
| MK749917 | MK749917_A.D.2.2 | A.D.2.2 | Nicaragua | 2016 |
| MZ515559 | MZ515559_A.D.2.2 | A.D.2.2 | United Kingdom | 2018 |
| MZ515703 | MZ515703_A.D.2.2 | A.D.2.2 | Netherlands | 2018 |
| MZ515939 | MZ515939_A.D.2.2 | A.D.2.2 | Netherlands | 2017 |
| MZ516108 | MZ516108_A.D.2.2 | A.D.2.2 | Netherlands | 2017 |
| MZ516129 | MZ516129_A.D.2.2 | A.D.2.2 | United Kingdom | 2019 |
| ON237253 | ON237253_A.D.2.2 | A.D.2.2 | Argentina | 2015 |
| ON237297 | ON237297_A.D.2.2 | A.D.2.2 | Argentina | 2017 |
| ON237301 | ON237301_A.D.2.2 | A.D.2.2 | Argentina | 2017 |
| ON237307 | ON237307_A.D.2.2 | A.D.2.2 | Argentina | 2017 |
| ON237311 | ON237311_A.D.2.2 | A.D.2.2 | Argentina | 2017 |
| ON237321 | ON237321_A.D.2.2 | A.D.2.2 | Argentina | 2017 |
| ON237334 | ON237334_A.D.2.2 | A.D.2.2 | Argentina | 2017 |
| ON237338 | ON237338_A.D.2.2 | A.D.2.2 | Argentina | 2017 |
| ON237339 | ON237339_A.D.2.2 | A.D.2.2 | Argentina | 2017 |
| MZ515850 | MZ515850_A.D.2.2.1 | A.D.2.2.1 | United Kingdom | 2018 |
| MZ515887 | MZ515887_A.D.2.2.1 | A.D.2.2.1 | Spain | 2019 |
| MZ515921 | MZ515921_A.D.2.2.1 | A.D.2.2.1 | United Kingdom | 2018 |
| MZ516026 | MZ516026_A.D.2.2.1 | A.D.2.2.1 | Netherlands | 2019 |
| MZ516028 | MZ516028_A.D.2.2.1 | A.D.2.2.1 | United Kingdom | 2018 |
| MZ516052 | MZ516052_A.D.2.2.1 | A.D.2.2.1 | Spain | 2019 |
| ON237295 | ON237295_A.D.2.2.1 | A.D.2.2.1 | Argentina | 2017 |
| LC816567 | LC816567_A.D.3.7 | A.D.3 | Japan | 2022 |
| LC847013 | LC847013_A.D.3 | A.D.3 | Myanmar | 2021 |
| LC847032 | LC847032_A.D.3 | A.D.3 | Myanmar | 2021 |
| LC816568 | LC816568_A.D.3 | A.D.3 | Japan | 2023 |
| LC847039 | LC847039_A.D.3 | A.D.3 | Myanmar | 2021 |
| LC847042 | LC847042_A.D.3 | A.D.3 | Myanmar | 2021 |
| LC847045 | LC847045_A.D.3 | A.D.3 | Myanmar | 2022 |
| MW678420 | MW678420_A.D.3 | A.D.3 | Thailand | 2020 |
| MW678434 | MW678434_A.D.3 | A.D.3 | Thailand | 2020 |
| MW678439 | MW678439_A.D.3 | A.D.3 | Thailand | 2020 |
| MW678474 | MW678474_A.D.3 | A.D.3 | Thailand | 2020 |
| MW678507 | MW678507_A.D.3 | A.D.3 | Thailand | 2020 |
| MW678510 | MW678510_A.D.3 | A.D.3 | Thailand | 2020 |
| MW678558 | MW678558_A.D.3 | A.D.3 | Thailand | 2020 |
| ON707422 | ON707422_A.D.3 | A.D.3 | China | 2020 |
| OP554442 | OP554442_A.D.3 | A.D.3 | Saudi Arabia | 2022 |
| OP554446 | OP554446_A.D.3 | A.D.3 | Saudi Arabia | 2020 |
| OP927855 | OP927855_A.D.3.1 | A.D.3 | Germany | 2021 |
| OQ024122 | OQ024122_A.D.3 | A.D.3 | USA | 2022 |
| OR143140 | OR143140_A.D.3.2 | A.D.3 | USA | 2022 |
| OR143210 | OR143210_A.D.3 | A.D.3 | USA | 2023 |
| OR522481 | OR522481_A.D.3 | A.D.3 | USA | 2022 |
| OR522493 | OR522493_A.D.3.2 | A.D.3 | USA | 2022 |
| OR666558 | OR666558_A.D.3 | A.D.3 | China | 2020 |
| OR795390 | OR795390_A.D.3 | A.D.3 | Germany | 2020 |
| OR795420 | OR795420_A.D.3 | A.D.3 | Germany | 2021 |
| OR795432 | OR795432_A.D.3 | A.D.3 | Germany | 2021 |
| OR795434 | OR795434_A.D.3.1 | A.D.3 | Germany | 2021 |
| OR795466 | OR795466_A.D.3 | A.D.3 | Germany | 2021 |
| OR840704 | OR840704_A.D.3 | A.D.3 | China | 2023 |
| OR840706 | OR840706_A.D.3 | A.D.3 | China | 2023 |
| OR872621 | OR872621_A.D.3.2 | A.D.3 | USA | 2022 |
| OR872636 | OR872636_A.D.3 | A.D.3 | USA | 2022 |
| OR882972 | OR882972_A.D.3.3 | A.D.3 | USA | 2023 |
| OR882992 | OR882992_A.D.3 | A.D.3 | USA | 2023 |
| OR883017 | OR883017_A.D.3 | A.D.3 | USA | 2023 |
| OR915772 | OR915772_A.D.3.3 | A.D.3 | USA | 2023 |
| PP135009 | PP135009_A.D.3 | A.D.3 | USA | 2023 |
| PP270234 | PP270234_A.D.3 | A.D.3 | USA | 2023 |
| PP270237 | PP270237_A.D.3 | A.D.3 | USA | 2023 |
| PP270249 | PP270249_A.D.3 | A.D.3 | USA | 2023 |
| PP376348 | PP376348_A.D.3 | A.D.3 | Russia | 2020 |
| PP376432 | PP376432_A.D.3 | A.D.3 | Russia | 2020 |
| PP411979 | PP411979_A.D.3 | A.D.3 | Thailand | 2023 |
| PP495918 | PP495918_A.D.3 | A.D.3 | USA | 2020 |
| PP504654 | PP504654_A.D.3 | A.D.3 | USA | 2023 |
| PP508181 | PP508181_A.D.3.2 | A.D.3 | Peru | 2022 |
| PP530247 | PP530247_A.D.3.10 | A.D.3 | USA | 2023 |
| PP530264 | PP530264_A.D.3.3 | A.D.3 | USA | 2023 |
| PP530269 | PP530269_A.D.3.3 | A.D.3 | USA | 2024 |
| PP681250 | PP681250_A.D.3 | A.D.3 | Japan | 2022 |
| PP681251 | PP681251_A.D.3 | A.D.3 | USA | 2022 |
| PP681264 | PP681264_A.D.3.3 | A.D.3 | USA | 2022 |
| PP776593 | PP776593_A.D.3.3 | A.D.3 | USA | 2024 |
| PP781414 | PP781414_A.D.3.5 | A.D.3 | USA | 2024 |
| PP819400 | PP819400_A.D.3 | A.D.3 | China | 2024 |
| PP833560 | PP833560_A.D.3 | A.D.3 | China | 2024 |
| PP903815 | PP903815_A.D.3.3 | A.D.3 | USA | 2024 |
| PP934458 | PP934458_A.D.3.3 | A.D.3 | USA | 2024 |
| PP957764 | PP957764_A.D.3.12 | A.D.3 | USA | 2024 |
| PP970045 | PP970045_A.D.3.1 | A.D.3 | Ireland | 2023 |
| PP973976 | PP973976_A.D.3 | A.D.3 | China | 2024 |
| PP973981 | PP973981_A.D.3 | A.D.3 | China | 2024 |
| PP974135 | PP974135_A.D.3 | A.D.3 | China | 2020 |
| PP974148 | PP974148_A.D.3 | A.D.3 | China | 2020 |
| PP974154 | PP974154_A.D.3 | A.D.3 | China | 2022 |
| PP974155 | PP974155_A.D.3 | A.D.3 | China | 2023 |
| PP974160 | PP974160_A.D.3.7 | A.D.3 | China | 2024 |
| PP974162 | PP974162_A.D.3 | A.D.3 | China | 2023 |
| PP974163 | PP974163_A.D.3 | A.D.3 | China | 2023 |
| PP974167 | PP974167_A.D.3 | A.D.3 | China | 2022 |
| PP974169 | PP974169_A.D.3 | A.D.3 | China | 2024 |
| PQ066217 | PQ066217_A.D.3.5 | A.D.3 | USA | 2023 |
| PQ117650 | PQ117650_A.D.3.7 | A.D.3 | USA | 2024 |
| PQ192487 | PQ192487_A.D.3 | A.D.3 | Thailand | 2023 |
| PQ192488 | PQ192488_A.D.3.1 | A.D.3 | Thailand | 2023 |
| PQ117653 | PQ117653_A.D.3 | A.D.3 | USA | 2024 |
| PQ348814 | PQ348814_A.D.3.1 | A.D.3 | United Kingdom | 2021 |
| PQ348983 | PQ348983_A.D.3 | A.D.3 | United Kingdom | 2020 |
| PQ626971 | PQ626971_A.D.3 | A.D.3 | China | 2024 |
| PQ618043 | PQ618043_A.D.3 | A.D.3 | Panama | 2024 |
| PQ618048 | PQ618048_A.D.3.2 | A.D.3 | Panama | 2024 |
| PQ618055 | PQ618055_A.D.3.3 | A.D.3 | Panama | 2022 |
| PQ618058 | PQ618058_A.D.3 | A.D.3 | Panama | 2023 |
| PQ638678 | PQ638678_A.D.3 | A.D.3 | USA | 2022 |
| PQ638715 | PQ638715_A.D.3.3 | A.D.3 | USA | 2023 |
| PQ762495 | PQ762495_A.D.3.1 | A.D.3 | USA | 2024 |
| PQ762736 | PQ762736_A.D.3.1 | A.D.3 | France | 2023 |
| PQ762850 | PQ762850_A.D.3 | A.D.3 | France | 2023 |
| PQ762890 | PQ762890_A.D.3 | A.D.3 | France | 2023 |
| PQ762926 | PQ762926_A.D.3 | A.D.3 | France | 2023 |
| PQ762940 | PQ762940_A.D.3.3 | A.D.3 | France | 2023 |
| PQ762990 | PQ762990_A.D.3.2 | A.D.3 | France | 2023 |
| PQ762999 | PQ762999_A.D.3 | A.D.3 | France | 2023 |
| PQ763159 | PQ763159_A.D.3 | A.D.3 | France | 2023 |
| PQ788236 | PQ788236_A.D.3.1 | A.D.3 | USA | 2024 |
| PP270209 | PP270209_A.D.3 | A.D.3 | USA | 2023 |
| OZ278991 | OZ278991_A.D.3.8 | A.D.3 | Spain | 2025 |
| MN306017 | MN306017_A.D.3 | A.D.3 | USA | 2018 |
| MZ515619 | MZ515619_A.D.3 | A.D.3 | Spain | 2019 |
| MZ515752 | MZ515752_A.D.3 | A.D.3 | United Kingdom | 2018 |
| OP744432 | OP744432_A.D.4 | A.D.4 | Kenya | 2021 |
| OP744433 | OP744433_A.D.4 | A.D.4 | Kenya | 2021 |
| OP744429 | OP744429_A.D.4 | A.D.4 | Kenya | 2021 |
| OP744445 | OP744445_A.D.4 | A.D.4 | Kenya | 2022 |
| OR162239 | OR162239_A.D.4 | A.D.4 | Kenya | 2022 |
| PP377512 | PP377512_A.D.4.1 | A.D.4 | South Africa | 2020 |
| PP377526 | PP377526_A.D.4 | A.D.4 | Russia | 2020 |
| PQ638686 | PQ638686_A.D.4 | A.D.4 | USA | 2023 |
| PQ762662 | PQ762662_A.D.4 | A.D.4 | France | 2023 |
| MH181991 | MH181991_A.D.4 | A.D.4 | Kenya | 2013 |
| MH182008 | MH182008_A.D.4 | A.D.4 | Kenya | 2014 |
| MH182018 | MH182018_A.D.4 | A.D.4 | Kenya | 2014 |
| MH182021 | MH182021_A.D.4 | A.D.4 | Kenya | 2014 |
| MH182027 | MH182027_A.D.4 | A.D.4 | Kenya | 2014 |
| MH182035 | MH182035_A.D.4 | A.D.4 | Kenya | 2015 |
| MH182043 | MH182043_A.D.4 | A.D.4 | Kenya | 2015 |
| MH182054 | MH182054_A.D.4 | A.D.4 | Kenya | 2016 |
| MH182059 | MH182059_A.D.4 | A.D.4 | Kenya | 2016 |
| MH182061 | MH182061_A.D.4 | A.D.4 | Kenya | 2016 |
| MZ515682 | MZ515682_A.D.4.1 | A.D.4 | United Kingdom | 2019 |
| OP320397 | OP320397_A.D.5 | A.D.5 | Philippines | 2020 |
| OP744430 | OP744430_A.D.5 | A.D.5 | Kenya | 2021 |
| OP744453 | OP744453_A.D.5 | A.D.5 | Kenya | 2021 |
| OP744457 | OP744457_A.D.5 | A.D.5 | Kenya | 2022 |
| OP927895 | OP927895_A.D.5 | A.D.5 | Germany | 2021 |
| OR162273 | OR162273_A.D.5 | A.D.5 | Kenya | 2022 |
| OR795388 | OR795388_A.D.5 | A.D.5 | Germany | 2020 |
| OR795389 | OR795389_A.D.5 | A.D.5 | Germany | 2020 |
| OR795397 | OR795397_A.D.5 | A.D.5 | Germany | 2020 |
| OR795422 | OR795422_A.D.5 | A.D.5 | Germany | 2021 |
| OR795428 | OR795428_A.D.5 | A.D.5 | Germany | 2021 |
| OR795433 | OR795433_A.D.5 | A.D.5 | Germany | 2021 |
| OR795456 | OR795456_A.D.5 | A.D.5 | Germany | 2021 |
| OR795465 | OR795465_A.D.5 | A.D.5 | Germany | 2021 |
| PP376406 | PP376406_A.D.5 | A.D.5 | Canada | 2020 |
| PP973987 | PP973987_A.D.5 | A.D.5 | China | 2024 |
| MZ515592 | MZ515592_A.D.5 | A.D.5 | Netherlands | 2019 |
| MZ516134 | MZ516134_A.D.5 | A.D.5 | Netherlands | 2019 |
| ON237320 | ON237320_A.D.5 | A.D.5 | Argentina | 2017 |
| OP320394 | OP320394_A.D.5 | A.D.5 | Philippines | 2019 |
| OQ024142 | OQ024142_A.D.5.1 | A.D.5.1 | USA | 2022 |
| OQ171895 | OQ171895_A.D.5.1 | A.D.5.1 | USA | 2022 |
| OR872620 | OR872620_A.D.5.1 | A.D.5.1 | USA | 2022 |
| OR872624 | OR872624_A.D.5.1 | A.D.5.1 | USA | 2022 |
| OR872630 | OR872630_A.D.5.1 | A.D.5.1 | USA | 2022 |
| OR872639 | OR872639_A.D.5.1 | A.D.5.1 | USA | 2022 |
| OR915738 | OR915738_A.D.5.1 | A.D.5.1 | USA | 2022 |
| PP270240 | PP270240_A.D.5.1 | A.D.5.1 | USA | 2023 |
| PP770464 | PP770464_A.D.5.1 | A.D.5.1 | USA | 2024 |
| PP795158 | PP795158_A.D.5.1 | A.D.5.1 | USA | 2024 |
| PP957777 | PP957777_A.D.5.1 | A.D.5.1 | USA | 2024 |
| PP959049 | PP959049_A.D.5.1 | A.D.5.1 | USA | 2024 |
| PP970038 | PP970038_A.D.5.1 | A.D.5.1 | Ireland | 2023 |
| PQ117651 | PQ117651_A.D.5.1 | A.D.5.1 | USA | 2024 |
| PQ348810 | PQ348810_A.D.5.1 | A.D.5.1 | United Kingdom | 2021 |
| PQ762669 | PQ762669_A.D.5.1 | A.D.5.1 | France | 2023 |
| PQ762678 | PQ762678_A.D.5.1 | A.D.5.1 | France | 2023 |
| PQ762717 | PQ762717_A.D.5.1 | A.D.5.1 | France | 2024 |
| PQ762739 | PQ762739_A.D.5.1 | A.D.5.1 | France | 2023 |
| PQ762747 | PQ762747_A.D.5.1 | A.D.5.1 | France | 2023 |
| PQ762777 | PQ762777_A.D.5.1 | A.D.5.1 | France | 2024 |
| PQ762831 | PQ762831_A.D.5.1 | A.D.5.1 | France | 2023 |
| PQ762854 | PQ762854_A.D.5.1 | A.D.5.1 | France | 2023 |
| PQ762869 | PQ762869_A.D.5.1 | A.D.5.1 | France | 2023 |
| PQ762912 | PQ762912_A.D.5.1 | A.D.5.1 | France | 2023 |
| PQ762968 | PQ762968_A.D.5.1 | A.D.5.1 | France | 2023 |
| PQ763019 | PQ763019_A.D.5.1 | A.D.5.1 | France | 2023 |
| PQ763062 | PQ763062_A.D.5.1 | A.D.5.1 | France | 2023 |
| MZ516012 | MZ516012_A.D.5.1 | A.D.5.1 | United Kingdom | 2019 |
| LC816569 | LC816569_A.D.5.2 | A.D.5.2 | Japan | 2023 |
| OP927821 | OP927821_A.D.5.2 | A.D.5.2 | Germany | 2021 |
| OP927827 | OP927827_A.D.5.2 | A.D.5.2 | Germany | 2021 |
| OP927843 | OP927843_A.D.5.2 | A.D.5.2 | Germany | 2021 |
| OP927880 | OP927880_A.D.5.2 | A.D.5.2 | Germany | 2021 |
| OP927955 | OP927955_A.D.5.2 | A.D.5.2 | Germany | 2021 |
| OQ024118 | OQ024118_A.D.5.2 | A.D.5.2 | USA | 2022 |
| OQ024129 | OQ024129_A.D.5.2 | A.D.5.2 | USA | 2022 |
| OQ024131 | OQ024131_A.D.5.2 | A.D.5.2 | USA | 2022 |
| OQ024147 | OQ024147_A.D.5.2 | A.D.5.2 | USA | 2022 |
| OQ171902 | OQ171902_A.D.5.2 | A.D.5.2 | USA | 2022 |
| OQ261752 | OQ261752_A.D.5.2 | A.D.5.2 | Austria | 2022 |
| OR143136 | OR143136_A.D.5.2 | A.D.5.2 | USA | 2022 |
| OR143141 | OR143141_A.D.5.2 | A.D.5.2 | USA | 2022 |
| OR143158 | OR143158_A.D.5.2 | A.D.5.2 | USA | 2023 |
| OR143182 | OR143182_A.D.5.2 | A.D.5.2 | USA | 2023 |
| OR143185 | OR143185_A.D.5.2 | A.D.5.2 | USA | 2023 |
| OR143191 | OR143191_A.D.5.2 | A.D.5.2 | USA | 2022 |
| OR143211 | OR143211_A.D.5.2 | A.D.5.2 | USA | 2023 |
| OR143215 | OR143215_A.D.5.2 | A.D.5.2 | USA | 2022 |
| OR143219 | OR143219_A.D.5.2 | A.D.5.2 | USA | 2022 |
| OR143214 | OR143214_A.D.5.2 | A.D.5.2 | USA | 2023 |
| OR493271 | OR493271_A.D.5.2 | A.D.5.2 | Spain | 2022 |
| OR493274 | OR493274_A.D.5.2 | A.D.5.2 | Spain | 2022 |
| OR493276 | OR493276_A.D.5.2 | A.D.5.2 | Spain | 2022 |
| OR493277 | OR493277_A.D.5.2 | A.D.5.2 | Spain | 2022 |
| OR493278 | OR493278_A.D.5.2 | A.D.5.2 | Spain | 2022 |
| OR493279 | OR493279_A.D.5.2 | A.D.5.2 | Spain | 2022 |
| OR493280 | OR493280_A.D.5.2 | A.D.5.2 | Spain | 2022 |
| OR493281 | OR493281_A.D.5.2 | A.D.5.2 | Spain | 2022 |
| OR493282 | OR493282_A.D.5.2 | A.D.5.2 | Spain | 2022 |
| OR493283 | OR493283_A.D.5.2 | A.D.5.2 | Spain | 2022 |
| OR493284 | OR493284_A.D.5.2 | A.D.5.2 | Spain | 2022 |
| OR493288 | OR493288_A.D.5.2 | A.D.5.2 | Spain | 2022 |
| OR493291 | OR493291_A.D.5.2 | A.D.5.2 | Spain | 2022 |
| OR493292 | OR493292_A.D.5.2 | A.D.5.2 | Spain | 2022 |
| OR493295 | OR493295_A.D.5.2 | A.D.5.2 | Spain | 2022 |
| OR493299 | OR493299_A.D.5.2 | A.D.5.2 | Spain | 2022 |
| OR493300 | OR493300_A.D.5.2 | A.D.5.2 | Spain | 2022 |
| OR493302 | OR493302_A.D.5.2 | A.D.5.2 | Spain | 2022 |
| OR493304 | OR493304_A.D.5.2 | A.D.5.2 | Spain | 2022 |
| OR493315 | OR493315_A.D.5.2 | A.D.5.2 | Spain | 2021 |
| OR522463 | OR522463_A.D.5.2 | A.D.5.2 | USA | 2022 |
| OR522480 | OR522480_A.D.5.2 | A.D.5.2 | USA | 2022 |
| OR795427 | OR795427_A.D.5.2 | A.D.5.2 | Germany | 2021 |
| OR795436 | OR795436_A.D.5.2 | A.D.5.2 | Germany | 2021 |
| OR795457 | OR795457_A.D.5.2 | A.D.5.2 | Germany | 2021 |
| OR795461 | OR795461_A.D.5.2 | A.D.5.2 | Germany | 2021 |
| OR795470 | OR795470_A.D.5.2 | A.D.5.2 | Germany | 2021 |
| OR795471 | OR795471_A.D.5.2 | A.D.5.2 | Germany | 2022 |
| OR872591 | OR872591_A.D.5.2 | A.D.5.2 | USA | 2022 |
| OR872592 | OR872592_A.D.5.2 | A.D.5.2 | USA | 2022 |
| OR872598 | OR872598_A.D.5.2 | A.D.5.2 | USA | 2022 |
| OR872613 | OR872613_A.D.5.2 | A.D.5.2 | USA | 2022 |
| OR872617 | OR872617_A.D.5.2 | A.D.5.2 | USA | 2022 |
| OR872629 | OR872629_A.D.5.2 | A.D.5.2 | USA | 2022 |
| OR872634 | OR872634_A.D.5.2 | A.D.5.2 | USA | 2022 |
| OR882970 | OR882970_A.D.5.2 | A.D.5.2 | USA | 2022 |
| OR882973 | OR882973_A.D.5.2 | A.D.5.2 | USA | 2022 |
| OR882979 | OR882979_A.D.5.2 | A.D.5.2 | USA | 2022 |
| OR883004 | OR883004_A.D.5.2 | A.D.5.2 | USA | 2022 |
| OR883011 | OR883011_A.D.5.2 | A.D.5.2 | USA | 2022 |
| OR883015 | OR883015_A.D.5.2 | A.D.5.2 | USA | 2022 |
| OR883016 | OR883016_A.D.5.2 | A.D.5.2 | USA | 2022 |
| OR883022 | OR883022_A.D.5.2 | A.D.5.2 | USA | 2023 |
| OR915746 | OR915746_A.D.5.2 | A.D.5.2 | USA | 2022 |
| OR915762 | OR915762_A.D.5.2 | A.D.5.2 | USA | 2022 |
| OR915776 | OR915776_A.D.5.2 | A.D.5.2 | USA | 2023 |
| PP237793 | PP237793_A.D.5.2 | A.D.5.2 | USA | 2023 |
| PP270233 | PP270233_A.D.5.2 | A.D.5.2 | USA | 2023 |
| PP270236 | PP270236_A.D.5.2 | A.D.5.2 | USA | 2023 |
| PP270235 | PP270235_A.D.5.2 | A.D.5.2 | USA | 2023 |
| PP270244 | PP270244_A.D.5.2 | A.D.5.2 | USA | 2023 |
| PP270254 | PP270254_A.D.5.2 | A.D.5.2 | USA | 2023 |
| PP270258 | PP270258_A.D.5.2 | A.D.5.2 | USA | 2023 |
| PP270267 | PP270267_A.D.5.2 | A.D.5.2 | USA | 2023 |
| PP270269 | PP270269_A.D.5.2 | A.D.5.2 | USA | 2023 |
| PP342418 | PP342418_A.D.5.2 | A.D.5.2 | USA | 2023 |
| PP401814 | PP401814_A.D.5.2 | A.D.5.2 | USA | 2023 |
| PP504649 | PP504649_A.D.5.2 | A.D.5.2 | USA | 2023 |
| PP508183 | PP508183_A.D.5.2 | A.D.5.2 | Peru | 2023 |
| PP508188 | PP508188_A.D.5.2 | A.D.5.2 | Peru | 2023 |
| PP530278 | PP530278_A.D.5.2 | A.D.5.2 | USA | 2023 |
| PP681254 | PP681254_A.D.5.2 | A.D.5.2 | USA | 2022 |
| PP681259 | PP681259_A.D.5.2 | A.D.5.2 | USA | 2022 |
| PP681258 | PP681258_A.D.5.2 | A.D.5.2 | USA | 2022 |
| PP681265 | PP681265_A.D.5.2 | A.D.5.2 | USA | 2022 |
| PP709391 | PP709391_A.D.5.2 | A.D.5.2 | USA | 2024 |
| PP709456 | PP709456_A.D.5.2 | A.D.5.2 | USA | 2024 |
| PP781379 | PP781379_A.D.5.2 | A.D.5.2 | USA | 2023 |
| PP781393 | PP781393_A.D.5.2 | A.D.5.2 | USA | 2024 |
| PP781391 | PP781391_A.D.5.2 | A.D.5.2 | USA | 2024 |
| PP781396 | PP781396_A.D.5.2 | A.D.5.2 | USA | 2024 |
| PP781398 | PP781398_A.D.5.2 | A.D.5.2 | USA | 2023 |
| PP781404 | PP781404_A.D.5.2 | A.D.5.2 | USA | 2023 |
| PP781411 | PP781411_A.D.5.2 | A.D.5.2 | USA | 2024 |
| PP781422 | PP781422_A.D.5.2 | A.D.5.2 | USA | 2024 |
| PP795135 | PP795135_A.D.5.2 | A.D.5.2 | USA | 2023 |
| PP795152 | PP795152_A.D.5.2 | A.D.5.2 | USA | 2023 |
| PP795153 | PP795153_A.D.5.2 | A.D.5.2 | USA | 2023 |
| PP795163 | PP795163_A.D.5.2 | A.D.5.2 | USA | 2024 |
| PP830379 | PP830379_A.D.5.2 | A.D.5.2 | USA | 2023 |
| PP830384 | PP830384_A.D.5.2 | A.D.5.2 | USA | 2023 |
| PP882670 | PP882670_A.D.5.2 | A.D.5.2 | USA | 2023 |
| PP882672 | PP882672_A.D.5.2 | A.D.5.2 | USA | 2023 |
| PP882676 | PP882676_A.D.5.2 | A.D.5.2 | USA | 2023 |
| PP882678 | PP882678_A.D.5.2 | A.D.5.2 | USA | 2023 |
| PP903819 | PP903819_A.D.5.2 | A.D.5.2 | USA | 2024 |
| PP903822 | PP903822_A.D.5.2 | A.D.5.2 | USA | 2022 |
| PP903833 | PP903833_A.D.5.2 | A.D.5.2 | USA | 2024 |
| PP910773 | PP910773_A.D.5.2 | A.D.5.2 | USA | 2024 |
| PP910781 | PP910781_A.D.5.2 | A.D.5.2 | USA | 2024 |
| PP934450 | PP934450_A.D.5.2 | A.D.5.2 | USA | 2024 |
| PP934455 | PP934455_A.D.5.2 | A.D.5.2 | USA | 2024 |
| PP934464 | PP934464_A.D.5.2 | A.D.5.2 | USA | 2024 |
| PP934467 | PP934467_A.D.5.2 | A.D.5.2 | USA | 2024 |
| PP957779 | PP957779_A.D.5.2 | A.D.5.2 | USA | 2024 |
| PP969955 | PP969955_A.D.5.2 | A.D.5.2 | Ireland | 2023 |
| PP969990 | PP969990_A.D.5.2 | A.D.5.2 | Ireland | 2023 |
| PP973756 | PP973756_A.D.5.2 | A.D.5.2 | USA | 2024 |
| PP973776 | PP973776_A.D.5.2 | A.D.5.2 | USA | 2024 |
| PP973957 | PP973957_A.D.5.2 | A.D.5.2 | China | 2023 |
| PP973964 | PP973964_A.D.5.2 | A.D.5.2 | China | 2023 |
| PP973966 | PP973966_A.D.5.2 | A.D.5.2 | China | 2023 |
| PP973973 | PP973973_A.D.5.2 | A.D.5.2 | China | 2023 |
| PP973979 | PP973979_A.D.5.2 | A.D.5.2 | China | 2024 |
| PQ618078 | PQ618078_A.D.5.2 | A.D.5.2 | Panama | 2023 |
| PQ638663 | PQ638663_A.D.5.2 | A.D.5.2 | USA | 2022 |
| PQ638687 | PQ638687_A.D.5.2 | A.D.5.2 | USA | 2022 |
| PQ638690 | PQ638690_A.D.5.2 | A.D.5.2 | USA | 2022 |
| PQ638694 | PQ638694_A.D.5.2 | A.D.5.2 | USA | 2022 |
| PQ638702 | PQ638702_A.D.5.2 | A.D.5.2 | USA | 2023 |
| PQ737348 | PQ737348_A.D.5.2 | A.D.5.2 | France | 2023 |
| PQ762501 | PQ762501_A.D.5.2 | A.D.5.2 | USA | 2024 |
| PQ762723 | PQ762723_A.D.5.2 | A.D.5.2 | France | 2023 |
| PQ762743 | PQ762743_A.D.5.2 | A.D.5.2 | France | 2023 |
| PQ762806 | PQ762806_A.D.5.2 | A.D.5.2 | France | 2023 |
| PQ762863 | PQ762863_A.D.5.2 | A.D.5.2 | France | 2023 |
| PQ762895 | PQ762895_A.D.5.2 | A.D.5.2 | France | 2023 |
| PQ762949 | PQ762949_A.D.5.2 | A.D.5.2 | France | 2023 |
| PQ762984 | PQ762984_A.D.5.2 | A.D.5.2 | France | 2023 |
| PQ763040 | PQ763040_A.D.5.2 | A.D.5.2 | France | 2023 |
| PQ763103 | PQ763103_A.D.5.2 | A.D.5.2 | France | 2024 |
| PQ763121 | PQ763121_A.D.5.2 | A.D.5.2 | France | 2023 |
| PQ788230 | PQ788230_A.D.5.2 | A.D.5.2 | USA | 2024 |
| OK500258 | OK500258_A.D.5.2 | A.D.5.2 | France | 2021 |
| OP890339 | OP890339_A.D.5.2 | A.D.5.2 | USA | 2022 |
| OP320396 | OP320396_A.D.5.3 | A.D.5.3 | Philippines | 2020 |
| OP320398 | OP320398_A.D.5.3 | A.D.5.3 | Philippines | 2020 |
| OR601480 | OR601480_A.D.5.3 | A.D.5.3 | USA | 2023 |
| OR795477 | OR795477_A.D.5.3 | A.D.5.3 | Germany | 2022 |
| OR883003 | OR883003_A.D.5.3 | A.D.5.3 | USA | 2023 |
| OR915765 | OR915765_A.D.5.3 | A.D.5.3 | USA | 2021 |
| OR915769 | OR915769_A.D.5.3 | A.D.5.3 | USA | 2021 |
| PP270238 | PP270238_A.D.5.3 | A.D.5.3 | USA | 2023 |
| PP495913 | PP495913_A.D.5.3 | A.D.5.3 | USA | 2020 |
| PP594810 | PP594810_A.D.5.3 | A.D.5.3 | USA | 2023 |
| PP681245 | PP681245_A.D.5.3 | A.D.5.3 | USA | 2022 |
| PP681257 | PP681257_A.D.5.3 | A.D.5.3 | USA | 2022 |
| PQ008873 | PQ008873_A.D.5.3 | A.D.5.3 | USA | 2024 |
| PQ348863 | PQ348863_A.D.5.3 | A.D.5.3 | United Kingdom | 2022 |
| PQ348887 | PQ348887_A.D.5.3 | A.D.5.3 | United Kingdom | 2022 |
| PQ638789 | PQ638789_A.D.5.3 | A.D.5.3 | USA | 2023 |
| PQ737354 | PQ737354_A.D.5.3 | A.D.5.3 | France | 2023 |
| PQ762800 | PQ762800_A.D.5.3 | A.D.5.3 | France | 2023 |
| PQ762903 | PQ762903_A.D.5.3 | A.D.5.3 | France | 2024 |
| PP237788 | PP237788_A.D.5.4 | A.D.5.4 | USA | 2023 |
| PP668168 | PP668168_A.D.5.4 | A.D.5.4 | USA | 2023 |
| PP709457 | PP709457_A.D.5.4 | A.D.5.4 | USA | 2023 |
| PP830405 | PP830405_A.D.5.4 | A.D.5.4 | USA | 2023 |
| PP957747 | PP957747_A.D.5.4 | A.D.5.4 | USA | 2024 |
| PP270208 | PP270208_A.D.5.4 | A.D.5.4 | USA | 2023 |

**Table S3. List of HRSV-A ON1 genotype G gene reference sequences for tMRCA analysis**

| **Subgroup** | **NCBI/GISAID ID** | **Sample collection date** | **Sampling country** | **Clade** |
| --- | --- | --- | --- | --- |
| A | EPI_ISL_6494785 | 2014 | United Kingdom | A.D.2.2 |
| A | EPI_ISL_6494806 | 2015 | United Kingdom | A.D.2.2 |
| A | EPI_ISL_6494988 | 2013 | United Kingdom | A.D.2.2 |
| A | EPI_ISL_6494993 | 2013 | United Kingdom | A.D.2.2 |
| A | EPI_ISL_15120716 | 2016 | Morocco | A.D.2.2 |
| A | EPI_ISL_6494809 | 2015 | United Kingdom | A.D.2.2 |
| A | EPI_ISL_15120721 | 2016 | Morocco | A.D.2.2 |
| A | EPI_ISL_15120743 | 2017 | Morocco | A.D.2.2 |
| A | EPI_ISL_15120745 | 2017 | Morocco | A.D.2.2 |
| A | EPI_ISL_15120692 | 2016 | Morocco | A.D.2.2 |
| A | EPI_ISL_15120694 | 2016 | Morocco | A.D.2.2 |
| A | EPI_ISL_15120715 | 2016 | Morocco | A.D.2.2 |
| A | EPI_ISL_15120727 | 2016 | Morocco | A.D.2.2 |
| A | EPI_ISL_15120783 | 2018 | Morocco | A.D.2.2 |
| A | EPI_ISL_15120774 | 2017 | Morocco | A.D.2.2 |
| A | EPI_ISL_15120706 | 2016 | Morocco | A.D.2.2 |
| A | EPI_ISL_15120681 | 2015 | Morocco | A.D.2.2 |
| A | EPI_ISL_15120785 | 2019 | Morocco | A.D.2.2 |
| A | EPI_ISL_6494821 | 2016 | United Kingdom | A.D.2.2 |
| A | EPI_ISL_15120688 | 2015 | Morocco | A.D.2.2 |
| A | EPI_ISL_6494811 | 2016 | United Kingdom | A.D.2.2 |
| A | EPI_ISL_15120689 | 2015 | Morocco | A.D.2.2 |
| A | EPI_ISL_15120730 | 2016 | Morocco | A.D.2.2 |
| A | EPI_ISL_15120717 | 2016 | Morocco | A.D.2.2 |
| A | EPI_ISL_6494800 | 2015 | United Kingdom | A.D.2.2 |
| A | EPI_ISL_15120728 | 2016 | Morocco | A.D.2.2 |
| A | EPI_ISL_6494788 | 2014 | United Kingdom | A.D.2 |
| A | EPI_ISL_15120670 | 2014 | Morocco | A.D.5 |
| A | EPI_ISL_15120671 | 2014 | Morocco | A.D.5 |
| A | EPI_ISL_15120665 | 2014 | Morocco | A.D |
| A | EPI_ISL_6494976 | 2012 | United Kingdom | A.D |
| A | EPI_ISL_6494983 | 2013 | United Kingdom | A.D |
| A | EPI_ISL_6494984 | 2013 | United Kingdom | A.D |
| A | EPI_ISL_6495010 | 2014 | United Kingdom | A.D |
| A | EPI_ISL_6494955 | 2012 | United Kingdom | A.D |
| A | EPI_ISL_6494958 | 2012 | United Kingdom | A.D |
| A | EPI_ISL_6494970 | 2012 | United Kingdom | A.D |
| A | EPI_ISL_6494971 | 2012 | United Kingdom | A.D |
| A | EPI_ISL_6494997 | 2013 | United Kingdom | A.D |
| A | EPI_ISL_15120674 | 2014 | Morocco | A.D |
| A | EPI_ISL_15120680 | 2015 | Morocco | A.D |
| A | EPI_ISL_15120690 | 2015 | Morocco | A.D |
| A | EPI_ISL_15120677 | 2014 | Morocco | A.D |
| A | EPI_ISL_15120682 | 2015 | Morocco | A.D |
| A | EPI_ISL_15120678 | 2015 | Morocco | A.D |
| A | EPI_ISL_15120679 | 2015 | Morocco | A.D |
| A | EPI_ISL_15004433 | 2012 | Morocco | A.D |
| A | EPI_ISL_15004435 | 2012 | Morocco | A.D |
| A | EPI_ISL_6494953 | 2012 | United Kingdom | A.D |
| A | EPI_ISL_6494954 | 2012 | United Kingdom | A.D |
| A | EPI_ISL_6494980 | 2012 | United Kingdom | A.D |
| A | EPI_ISL_6495009 | 2014 | United Kingdom | A.D |
| A | EPI_ISL_6495004 | 2014 | United Kingdom | A.D |
| A | EPI_ISL_6495001 | 2013 | United Kingdom | A.D |
| A | EPI_ISL_6494992 | 2013 | United Kingdom | A.D |
| A | EPI_ISL_6494995 | 2013 | United Kingdom | A.D |
| A | EPI_ISL_6494792 | 2014 | United Kingdom | A.D |
| A | EPI_ISL_15120666 | 2014 | Morocco | A.D |
| A | EPI_ISL_10954007 | 2017 | United Kingdom | A.D.2.2.1 |
| A | EPI_ISL_15120729 | 2016 | Morocco | A.D |
| A | EPI_ISL_6494794 | 2014 | United Kingdom | A.D |
| A | EPI_ISL_15120675 | 2014 | Morocco | A.D |
| A | EPI_ISL_10914989 | 2016 | Australia | A.D |
| A | EPI_ISL_6494820 | 2016 | United Kingdom | A.D.5 |
| A | EPI_ISL_15120775 | 2017 | Morocco | A.D.5 |
| A | EPI_ISL_6494822 | 2016 | United Kingdom | A.D.5 |
| A | EPI_ISL_15120781 | 2018 | Morocco | A.D.5 |
| A | EPI_ISL_15120780 | 2018 | Morocco | A.D.5 |
| A | EPI_ISL_12970403 | 2019 | Philippines | A.D.5 |
| A | EPI_ISL_17417584 | 2020 | Philippines | A.D.5 |
| A | EPI_ISL_12970407 | 2019 | Philippines | A.D.5 |
| A | EPI_ISL_12970417 | 2019 | Philippines | A.D.5 |
| A | EPI_ISL_12970412 | 2019 | Philippines | A.D.5 |
| A | EPI_ISL_15055323 | 2021 | Argentina | A.D.5.2 |
| A | EPI_ISL_15055328 | 2021 | Argentina | A.D.5.2 |
| A | EPI_ISL_15750187 | 2021 | France | A.D.5.2 |
| A | EPI_ISL_15750188 | 2021 | France | A.D.5.2 |
| A | EPI_ISL_16132377 | 2022 | USA | A.D.5.2 |
| A | EPI_ISL_16132339 | 2022 | USA | A.D.5.2 |
| A | EPI_ISL_16132408 | 2022 | USA | A.D.5.2 |
| A | EPI_ISL_16132392 | 2022 | USA | A.D.5.2 |
| A | EPI_ISL_16132398 | 2022 | USA | A.D.5.2 |
| A | EPI_ISL_16132350 | 2022 | USA | A.D.5.2 |
| A | EPI_ISL_16132395 | 2022 | USA | A.D.5.2 |
| A | EPI_ISL_16132355 | 2022 | USA | A.D.5.2 |
| A | EPI_ISL_16132405 | 2022 | USA | A.D.5.2 |
| A | EPI_ISL_16132328 | 2021 | USA | A.D.5.2 |
| A | EPI_ISL_16132361 | 2022 | USA | A.D.5.2 |
| A | EPI_ISL_16132368 | 2022 | USA | A.D.5.2 |
| A | EPI_ISL_15120786 | 2019 | Morocco | A.D.5 |
| A | EPI_ISL_11055745 | 2021 | South Africa | A.D.5.1 |
| A | EPI_ISL_11055747 | 2021 | South Africa | A.D.5.1 |
| A | EPI_ISL_11055753 | 2021 | South Africa | A.D.5.1 |
| A | EPI_ISL_11055748 | 2021 | South Africa | A.D.5.1 |
| A | EPI_ISL_11055760 | 2021 | South Africa | A.D.5.1 |
| A | EPI_ISL_11055729 | 2022 | South Africa | A.D.5.1 |
| A | EPI_ISL_14769848 | 2022 | South Africa | A.D.5.1 |
| A | EPI_ISL_11055768 | 2022 | South Africa | A.D.5.1 |
| A | EPI_ISL_16132363 | 2022 | USA | A.D.5.1 |
| A | EPI_ISL_16839038 | 2022 | United Kingdom | A.D.5.1 |
| A | EPI_ISL_15055315 | 2019 | Argentina | A.D.5 |
| A | EPI_ISL_15055318 | 2019 | Argentina | A.D.5 |
| A | EPI_ISL_15120719 | 2016 | Morocco | A.D |
| A | EPI_ISL_15120726 | 2016 | Morocco | A.D |
| A | EPI_ISL_15120776 | 2017 | Morocco | A.D |
| A | EPI_ISL_12970415 | 2019 | Philippines | A.D |
| A | EPI_ISL_6494816 | 2016 | United Kingdom | A.D |
| A | EPI_ISL_6494815 | 2016 | United Kingdom | A.D |
| A | EPI_ISL_12970405 | 2019 | Philippines | A.D |
| A | EPI_ISL_15120778 | 2017 | Morocco | A.D |
| A | EPI_ISL_6494818 | 2016 | United Kingdom | A.D |
| A | EPI_ISL_12970420 | 2019 | Philippines | A.D.3 |
| A | EPI_ISL_6174135 | 2019 | Cote dIvoire | A.D.3 |
| A | EPI_ISL_11055736 | 2021 | South Africa | A.D.3 |
| A | EPI_ISL_11055719 | 2022 | South Africa | A.D.3 |
| A | EPI_ISL_11055727 | 2022 | South Africa | A.D.3 |
| A | EPI_ISL_12870553 | 2022 | South Africa | A.D.3 |
| A | EPI_ISL_12870547 | 2022 | South Africa | A.D.3 |
| A | EPI_ISL_15896178 | 2022 | Australia | A.D.3 |
| A | EPI_ISL_16714774 | 2022 | Australia | A.D.3 |
| A | EPI_ISL_16132394 | 2022 | USA | A.D.3 |
| A | EPI_ISL_16681183 | 2022 | USA | A.D.3 |
| A | EPI_ISL_6268624 | 2021 | China | A.D.3 |
| A | EPI_ISL_6268619 | 2020 | China | A.D.3 |
| A | EPI_ISL_16132340 | 2022 | USA | A.D.3 |
| A | EPI_ISL_16132349 | 2022 | USA | A.D.3 |
| A | EPI_ISL_17066831 | 2022 | Australia | A.D.3 |
| A | EPI_ISL_16132356 | 2022 | USA | A.D.3 |
| A | EPI_ISL_16714809 | 2022 | Australia | A.D.3 |
| A | EPI_ISL_16132332 | 2022 | USA | A.D.3 |
| A | EPI_ISL_16132391 | 2022 | USA | A.D.3 |
| A | EPI_ISL_14039046 | 2021 | Uganda | A.D.3 |
| A | EPI_ISL_15896143 | 2022 | Australia | A.D.3.1 |
| A | EPI_ISL_15896187 | 2022 | Australia | A.D.3.1 |
| A | EPI_ISL_15896149 | 2022 | Australia | A.D.3.1 |
| A | EPI_ISL_15896169 | 2022 | Australia | A.D.3.1 |
| A | EPI_ISL_17066824 | 2022 | Australia | A.D.3.1 |
| A | EPI_ISL_17066833 | 2022 | Australia | A.D.3.1 |
| A | EPI_ISL_15896151 | 2022 | Australia | A.D.3.1 |
| A | EPI_ISL_15896158 | 2022 | Australia | A.D.3.1 |
| A | EPI_ISL_15896159 | 2022 | Australia | A.D.3.1 |
| A | EPI_ISL_15896170 | 2022 | Australia | A.D.3.1 |
| A | EPI_ISL_16839035 | 2022 | United Kingdom | A.D.3.1 |
| A | EPI_ISL_12970414 | 2019 | Philippines | A.D.5 |
| A | EPI_ISL_17417583 | 2020 | Philippines | A.D.5 |
| A | EPI_ISL_17417592 | 2020 | Philippines | A.D.5 |
| A | EPI_ISL_16959500 | 2021 | New Zealand | A.D.5.3 |
| A | EPI_ISL_16959949 | 2021 | New Zealand | A.D.5.3 |
| A | EPI_ISL_16959957 | 2021 | New Zealand | A.D.5.3 |
| A | EPI_ISL_16959653 | 2021 | New Zealand | A.D.5.3 |
| A | EPI_ISL_16959727 | 2021 | New Zealand | A.D.5.3 |
| A | EPI_ISL_16959603 | 2021 | New Zealand | A.D.5.3 |
| A | EPI_ISL_16959686 | 2021 | New Zealand | A.D.5.3 |
| A | EPI_ISL_16959665 | 2021 | New Zealand | A.D.5.3 |
| A | EPI_ISL_16960062 | 2021 | New Zealand | A.D.5.3 |
| A | EPI_ISL_16960076 | 2021 | New Zealand | A.D.5.3 |
| A | EPI_ISL_16959666 | 2021 | New Zealand | A.D.5.3 |
| A | EPI_ISL_16959871 | 2021 | New Zealand | A.D.5.3 |
| A | EPI_ISL_16959863 | 2021 | New Zealand | A.D.5.3 |
| A | EPI_ISL_16960047 | 2021 | New Zealand | A.D.5.3 |
| A | EPI_ISL_16960114 | 2021 | New Zealand | A.D.5.3 |
| A | EPI_ISL_16839032 | 2022 | United Kingdom | A.D.5 |
| A | EPI_ISL_16959620 | 2021 | New Zealand | A.D.5 |
| A | EPI_ISL_6208724 | 2020 | Egypt | A.D.5 |
| A | EPI_ISL_6208725 | 2020 | Egypt | A.D.5 |
| A | EPI_ISL_16960103 | 2021 | New Zealand | A.D |
| A | EPI_ISL_5522630 | 2019 | Cote dIvoire | A.D.2.2 |
| A | EPI_ISL_6174136 | 2019 | Cote dIvoire | A.D.2.2 |
| A | EPI_ISL_15055310 | 2018 | Argentina | A.D.1 |
| A | EPI_ISL_15055314 | 2018 | Argentina | A.D.1 |
| A | EPI_ISL_15774056 | 2021 | Australia | A.D.1.2 |
| A | EPI_ISL_15774063 | 2021 | Australia | A.D.1.2 |
| A | EPI_ISL_11055746 | 2021 | South Africa | A.D.1 |
| A | EPI_ISL_17089183 | 2020 | USA | A.D.1 |
| A | EPI_ISL_15120782 | 2018 | Morocco | A.D.1 |
| A | EPI_ISL_11817019 | 2021 | Australia | A.D.1 |
| A | EPI_ISL_15774035 | 2021 | Australia | A.D.1 |
| A | EPI_ISL_15774012 | 2021 | Australia | A.D.1 |
| A | EPI_ISL_16959973 | 2021 | New Zealand | A.D.1 |
| A | EPI_ISL_11055740 | 2021 | South Africa | A.D.1 |
| A | EPI_ISL_11055741 | 2021 | South Africa | A.D.1 |
| A | EPI_ISL_11055744 | 2021 | South Africa | A.D.1 |
| A | EPI_ISL_11055756 | 2021 | South Africa | A.D.1 |
| A | EPI_ISL_12529648 | 2021 | South Africa | A.D.1 |
| A | EPI_ISL_15055312 | 2018 | Argentina | A.D.1 |
| A | EPI_ISL_15055316 | 2019 | Argentina | A.D.1 |
| A | EPI_ISL_16839034 | 2022 | United Kingdom | A.D.1 |
| A | EPI_ISL_15055322 | 2019 | Argentina | A.D.1 |
| A | EPI_ISL_15055326 | 2021 | Argentina | A.D.1 |
| A | EPI_ISL_16681271 | 2022 | USA | A.D.1 |
| A | EPI_ISL_16681309 | 2022 | USA | A.D.1 |
| A | EPI_ISL_16132341 | 2022 | USA | A.D.1 |
| A | EPI_ISL_16132360 | 2022 | USA | A.D.1 |
| A | EPI_ISL_11817021 | 2021 | Australia | A.D.1.1 |
| A | EPI_ISL_11817041 | 2021 | Australia | A.D.1.1 |
| A | EPI_ISL_11817023 | 2021 | Australia | A.D.1.1 |
| A | EPI_ISL_11817039 | 2021 | Australia | A.D.1.1 |
| A | EPI_ISL_11817053 | 2021 | Australia | A.D.1.1 |
| A | EPI_ISL_11817036 | 2021 | Australia | A.D.1.1 |
| A | EPI_ISL_15774067 | 2021 | Australia | A.D.1.1 |
| A | EPI_ISL_15774068 | 2021 | Australia | A.D.1.1 |
| A | EPI_ISL_11817069 | 2021 | Australia | A.D.1.1 |
| A | EPI_ISL_15774070 | 2021 | Australia | A.D.1.1 |
| A | EPI_ISL_15896155 | 2022 | Australia | A.D.1 |
| A | EPI_ISL_16839037 | 2022 | United Kingdom | A.D.1 |
| A | EPI_ISL_16839033 | 2022 | United Kingdom | A.D.1 |
| A | EPI_ISL_16132345 | 2022 | USA | A.D.1 |
| A | EPI_ISL_16681323 | 2022 | USA | A.D.1 |
| A | EPI_ISL_16132376 | 2022 | USA | A.D.1 |
| A | EPI_ISL_11055715 | 2021 | South Africa | A.D.4.1 |
| A | EPI_ISL_11055737 | 2021 | South Africa | A.D.4.1 |
| A | EPI_ISL_12870543 | 2022 | South Africa | A.D.4.1 |
| A | EPI_ISL_11055743 | 2021 | South Africa | A.D.4.1 |
| A | EPI_ISL_11055718 | 2022 | South Africa | A.D.4.1 |
| A | EPI_ISL_11055716 | 2021 | South Africa | A.D.4.1 |
| A | EPI_ISL_11055738 | 2021 | South Africa | A.D.4.1 |
| A | EPI_ISL_12529639 | 2021 | South Africa | A.D.4.1 |
| A | EPI_ISL_11055717 | 2021 | South Africa | A.D.4.1 |
| A | EPI_ISL_11055752 | 2021 | South Africa | A.D.4.1 |
| A | EPI_ISL_11055749 | 2021 | South Africa | A.D.4.1 |
| A | EPI_ISL_12529635 | 2021 | South Africa | A.D.4.1 |
| A | EPI_ISL_12529638 | 2021 | South Africa | A.D.4.1 |
| A | EPI_ISL_11055742 | 2021 | South Africa | A.D.4.1 |
| A | EPI_ISL_16839036 | 2022 | United Kingdom | A.D.2 |
| A | OM857150 | 2020 | Australia | A.D.1.2 |
| A | OM857155 | 2020 | Australia | A.D.1.2 |
| A | OM857158 | 2020 | Australia | A.D.1.2 |
| A | OM857160 | 2020 | Australia | A.D.1.2 |
| A | OM857164 | 2020 | Australia | A.D.1.2 |
| A | OM857170 | 2020 | Australia | A.D.1.2 |
| A | OM857195 | 2021 | Australia | A.D.1.2 |
| A | KY967363 | 2015 | USA | A.D.1 |
| A | LR699736 | 2018 | United Kingdom | A.D.1 |
| A | MZ151854 | 2020 | Russia | A.D.1 |
| A | MZ221198 | 2018 | China | A.D.1 |
| A | MZ515570 | 2019 | Spain | A.D.1 |
| A | MZ515571 | 2020 | United Kingdom | A.D.1 |
| A | MZ515573 | 2020 | United Kingdom | A.D.1 |
| A | MZ515583 | 2019 | Spain | A.D.1 |
| A | MZ515585 | 2018 | Spain | A.D.1 |
| A | MZ515597 | 2019 | United Kingdom | A.D.1 |
| A | MZ515628 | 2019 | Netherlands | A.D.1 |
| A | MZ515631 | 2019 | Netherlands | A.D.1 |
| A | MZ515645 | 2019 | United Kingdom | A.D.1 |
| A | MZ515649 | 2019 | Netherlands | A.D.1 |
| A | MZ515652 | 2018 | United Kingdom | A.D.1 |
| A | MZ515688 | 2019 | Spain | A.D.1 |
| A | MZ515689 | 2018 | United Kingdom | A.D.1 |
| A | MZ515706 | 2020 | Spain | A.D.1 |
| A | MZ515744 | 2019 | Netherlands | A.D.1 |
| A | MZ515773 | 2020 | Spain | A.D.1 |
| A | MZ515777 | 2019 | Spain | A.D.1 |
| A | MZ515784 | 2019 | United Kingdom | A.D.1 |
| A | MZ515789 | 2018 | Netherlands | A.D.1 |
| A | MZ515803 | 2019 | Netherlands | A.D.1 |
| A | MZ515811 | 2018 | United Kingdom | A.D.1 |
| A | MZ515834 | 2019 | United Kingdom | A.D.1 |
| A | MZ515847 | 2018 | United Kingdom | A.D.1 |
| A | MZ515911 | 2019 | United Kingdom | A.D.1 |
| A | MZ515929 | 2018 | United Kingdom | A.D.1 |
| A | MZ515941 | 2019 | Spain | A.D.1 |
| A | MZ515945 | 2018 | Netherlands | A.D.1 |
| A | MZ515969 | 2019 | United Kingdom | A.D.1 |
| A | MZ515984 | 2019 | Spain | A.D.1 |
| A | MZ516002 | 2019 | Spain | A.D.1 |
| A | MZ516008 | 2018 | United Kingdom | A.D.1 |
| A | MZ516011 | 2020 | Spain | A.D.1 |
| A | MZ516029 | 2019 | United Kingdom | A.D.1 |
| A | MZ516058 | 2017 | Netherlands | A.D.1 |
| A | MZ516090 | 2019 | Netherlands | A.D.1 |
| A | MZ516137 | 2019 | United Kingdom | A.D.1 |
| A | ON237313 | 2017 | Argentina | A.D.1 |
| A | ON237314 | 2017 | Argentina | A.D.1 |
| A | ON237316 | 2017 | Argentina | A.D.1 |
| A | ON237343 | 2017 | Argentina | A.D.1 |
| A | ON237344 | 2017 | Argentina | A.D.1 |
| A | ON237350 | 2017 | Argentina | A.D.1 |
| A | OQ171919 | 2022 | USA | A.D.1 |
| A | OQ261747 | 2020 | Austria | A.D.1 |
| A | OQ261751 | 2022 | Austria | A.D.1 |
| A | MZ515651 | 2019 | Netherlands | A.D.2.1 |
| A | MZ515701 | 2019 | Netherlands | A.D.2.1 |
| A | MZ515709 | 2020 | Netherlands | A.D.2.1 |
| A | MZ516132 | 2019 | Netherlands | A.D.2.1 |
| A | MZ515569 | 2018 | United Kingdom | A.D.2.2.1 |
| A | MZ515575 | 2018 | United Kingdom | A.D.2.2.1 |
| A | MZ515609 | 2019 | Spain | A.D.2.2.1 |
| A | MZ515804 | 2019 | Spain | A.D.2.2.1 |
| A | MZ515850 | 2018 | United Kingdom | A.D.2.2.1 |
| A | MZ515851 | 2018 | Spain | A.D.2.2.1 |
| A | MZ515887 | 2019 | Spain | A.D.2.2.1 |
| A | MZ515902 | 2018 | Spain | A.D.2.2.1 |
| A | MZ515921 | 2018 | United Kingdom | A.D.2.2.1 |
| A | MZ516026 | 2019 | Netherlands | A.D.2.2.1 |
| A | MZ516028 | 2018 | United Kingdom | A.D.2.2.1 |
| A | MZ516052 | 2019 | Spain | A.D.2.2.1 |
| A | ON237289 | 2016 | Argentina | A.D.2.2.1 |
| A | ON237295 | 2017 | Argentina | A.D.2.2.1 |
| A | MH447958 | 2016 | Thailand | A.D.2.2 |
| A | MK749867 | 2016 | Nicaragua | A.D.2.2 |
| A | MK749890 | 2016 | Nicaragua | A.D.2.2 |
| A | MK749917 | 2016 | Nicaragua | A.D.2.2 |
| A | MN630100 | 2016 | USA | A.D.2.2 |
| A | MZ515559 | 2018 | United Kingdom | A.D.2.2 |
| A | MZ515566 | 2019 | United Kingdom | A.D.2.2 |
| A | MZ515703 | 2018 | Netherlands | A.D.2.2 |
| A | MZ515740 | 2019 | Netherlands | A.D.2.2 |
| A | MZ515833 | 2018 | Netherlands | A.D.2.2 |
| A | MZ515835 | 2018 | Netherlands | A.D.2.2 |
| A | MZ515841 | 2019 | Netherlands | A.D.2.2 |
| A | MZ515852 | 2019 | Netherlands | A.D.2.2 |
| A | MZ515939 | 2017 | Netherlands | A.D.2.2 |
| A | MZ515985 | 2019 | Netherlands | A.D.2.2 |
| A | MZ516072 | 2019 | United Kingdom | A.D.2.2 |
| A | MZ516108 | 2017 | Netherlands | A.D.2.2 |
| A | MZ516129 | 2019 | United Kingdom | A.D.2.2 |
| A | ON237253 | 2015 | Argentina | A.D.2.2 |
| A | ON237273 | 2015 | Argentina | A.D.2.2 |
| A | ON237297 | 2017 | Argentina | A.D.2.2 |
| A | ON237299 | 2017 | Argentina | A.D.2.2 |
| A | ON237301 | 2017 | Argentina | A.D.2.2 |
| A | ON237305 | 2017 | Argentina | A.D.2.2 |
| A | ON237307 | 2017 | Argentina | A.D.2.2 |
| A | ON237310 | 2017 | Argentina | A.D.2.2 |
| A | ON237311 | 2017 | Argentina | A.D.2.2 |
| A | ON237321 | 2017 | Argentina | A.D.2.2 |
| A | ON237334 | 2017 | Argentina | A.D.2.2 |
| A | ON237336 | 2017 | Argentina | A.D.2.2 |
| A | ON237338 | 2017 | Argentina | A.D.2.2 |
| A | ON237339 | 2017 | Argentina | A.D.2.2 |
| A | ON237356 | 2017 | Argentina | A.D.2.2 |
| A | ON237357 | 2017 | Argentina | A.D.2.2 |
| A | MZ515901 | 2019 | Netherlands | A.D.2 |
| A | ON237272 | 2015 | Argentina | A.D.2 |
| A | MN306017 | 2018 | USA | A.D.3 |
| A | MN306029 | 2019 | USA | A.D.3 |
| A | MN306045 | 2019 | USA | A.D.3 |
| A | MN306048 | 2019 | USA | A.D.3 |
| A | MT422269 | 2019 | Russia | A.D.3 |
| A | MT422271 | 2019 | Russia | A.D.3 |
| A | MZ151852 | 2020 | Russia | A.D.3 |
| A | MZ515555 | 2019 | United Kingdom | A.D.3 |
| A | MZ515619 | 2019 | Spain | A.D.3 |
| A | MZ515632 | 2018 | Netherlands | A.D.3 |
| A | MZ515647 | 2018 | United Kingdom | A.D.3 |
| A | MZ515666 | 2020 | United Kingdom | A.D.3 |
| A | MZ515668 | 2019 | United Kingdom | A.D.3 |
| A | MZ515679 | 2020 | United Kingdom | A.D.3 |
| A | MZ515692 | 2018 | Spain | A.D.3 |
| A | MZ515693 | 2020 | Spain | A.D.3 |
| A | MZ515723 | 2019 | United Kingdom | A.D.3 |
| A | MZ515752 | 2018 | United Kingdom | A.D.3 |
| A | MZ515854 | 2019 | United Kingdom | A.D.3 |
| A | MZ516017 | 2019 | United Kingdom | A.D.3 |
| A | MZ516023 | 2020 | United Kingdom | A.D.3 |
| A | MZ516027 | 2019 | United Kingdom | A.D.3 |
| A | MZ516039 | 2019 | Netherlands | A.D.3 |
| A | MZ516112 | 2019 | United Kingdom | A.D.3 |
| A | OK500256 | 2021 | France | A.D.3 |
| A | ON152648 | 2021 | Russia | A.D.3 |
| A | OP890340 | 2022 | USA | A.D.3 |
| A | OQ261748 | 2021 | Austria | A.D.3 |
| A | MH181991 | 2013 | Kenya | A.D.4 |
| A | MH181997 | 2013 | Kenya | A.D.4 |
| A | MH181998 | 2014 | Kenya | A.D.4 |
| A | MH182008 | 2014 | Kenya | A.D.4 |
| A | MH182012 | 2014 | Kenya | A.D.4 |
| A | MH182018 | 2014 | Kenya | A.D.4 |
| A | MH182021 | 2014 | Kenya | A.D.4 |
| A | MH182027 | 2014 | Kenya | A.D.4 |
| A | MH182028 | 2015 | Kenya | A.D.4 |
| A | MH182029 | 2015 | Kenya | A.D.4 |
| A | MH182030 | 2015 | Kenya | A.D.4 |
| A | MH182031 | 2015 | Kenya | A.D.4 |
| A | MH182035 | 2015 | Kenya | A.D.4 |
| A | MH182037 | 2015 | Kenya | A.D.4 |
| A | MH182043 | 2015 | Kenya | A.D.4 |
| A | MH182054 | 2016 | Kenya | A.D.4 |
| A | MH182059 | 2016 | Kenya | A.D.4 |
| A | MH182060 | 2016 | Kenya | A.D.4 |
| A | MH182061 | 2016 | Kenya | A.D.4 |
| A | MZ515567 | 2019 | United Kingdom | A.D.5.1 |
| A | MZ515749 | 2019 | United Kingdom | A.D.5.1 |
| A | MZ516012 | 2019 | United Kingdom | A.D.5.1 |
| A | OP890318 | 2022 | USA | A.D.5.2 |
| A | OP890332 | 2022 | USA | A.D.5.2 |
| A | OP890339 | 2022 | USA | A.D.5.2 |
| A | MZ515592 | 2019 | Netherlands | A.D.5 |
| A | MZ515780 | 2019 | Netherlands | A.D.5 |
| A | MZ515782 | 2018 | United Kingdom | A.D.5 |
| A | MZ515825 | 2019 | Netherlands | A.D.5 |
| A | MZ516134 | 2019 | Netherlands | A.D.5 |
| A | ON237271 | 2015 | Argentina | A.D.5 |
| A | ON237320 | 2017 | Argentina | A.D.5 |
| A | OP320394 | 2019 | Philippines | A.D.5 |
| A | KY654512 | 2012 | Philippines | A.D |
| A | KY654513 | 2013 | Philippines | A.D |
| A | KY654514 | 2013 | Philippines | A.D |
| A | KY654515 | 2012 | Philippines | A.D |
| A | KY654516 | 2013 | Philippines | A.D |
| A | KY654517 | 2013 | Philippines | A.D |
| A | KY982516 | 2013 | USA | A.D |
| A | KY982517 | 2013 | USA | A.D |
| A | LR699734 | 2018 | United Kingdom | A.D |
| A | MF001057 | 2015 | USA | A.D |
| A | MF614946 | 2012 | China | A.D |
| A | MF614947 | 2013 | China | A.D |
| A | MH181908 | 2012 | Kenya | A.D |
| A | MH181915 | 2012 | Kenya | A.D |
| A | MH181919 | 2012 | Kenya | A.D |
| A | MH181922 | 2012 | Kenya | A.D |
| A | MH181926 | 2012 | Kenya | A.D |
| A | MH181929 | 2012 | Kenya | A.D |
| A | MH181932 | 2012 | Kenya | A.D |
| A | MH181934 | 2012 | Kenya | A.D |
| A | MH181935 | 2012 | Kenya | A.D |
| A | MH181936 | 2012 | Kenya | A.D |
| A | MH181940 | 2012 | Kenya | A.D |
| A | MH181943 | 2012 | Kenya | A.D |
| A | MH181945 | 2012 | Kenya | A.D |
| A | MH181948 | 2012 | Kenya | A.D |
| A | MH181961 | 2012 | Kenya | A.D |
| A | MH181969 | 2012 | Kenya | A.D |
| A | MH181970 | 2012 | Kenya | A.D |
| A | MH181972 | 2012 | Kenya | A.D |
| A | MH181976 | 2012 | Kenya | A.D |
| A | MH181978 | 2013 | Kenya | A.D |
| A | MH181981 | 2013 | Kenya | A.D |
| A | MH181982 | 2013 | Kenya | A.D |
| A | MH181987 | 2013 | Kenya | A.D |
| A | MH182025 | 2014 | Kenya | A.D |
| A | MH290724 | 2017 | China | A.D |
| A | MH447951 | 2011 | Thailand | A.D |
| A | MH447952 | 2012 | Thailand | A.D |
| A | MH447953 | 2013 | Thailand | A.D |
| A | MH447955 | 2014 | Thailand | A.D |
| A | MH447957 | 2016 | Thailand | A.D |
| A | MH447959 | 2017 | Thailand | A.D |
| A | MH447960 | 2017 | Thailand | A.D |
| A | MK167035 | 2011 | USA | A.D |
| A | MK749884 | 2016 | Nicaragua | A.D |
| A | MK749895 | 2016 | Nicaragua | A.D |
| A | MN630090 | 2016 | USA | A.D |
| A | MN630093 | 2016 | USA | A.D |
| A | MN630099 | 2016 | USA | A.D |
| A | MN630104 | 2016 | USA | A.D |
| A | MN630106 | 2016 | USA | A.D |
| A | MW020596 | 2018 | Australia | A.D |
| A | MW020597 | 2018 | Australia | A.D |
| A | MW020598 | 2017 | Australia | A.D |
| A | MW582528 | 2015 | Germany | A.D |
| A | MZ221194 | 2018 | China | A.D |
| A | MZ221195 | 2014 | China | A.D |
| A | MZ221196 | 2014 | China | A.D |
| A | MZ221197 | 2018 | China | A.D |
| A | MZ221199 | 2018 | China | A.D |
| A | MZ221205 | 2014 | China | A.D |
| A | MZ515600 | 2018 | United Kingdom | A.D |
| A | MZ515620 | 2018 | Netherlands | A.D |
| A | MZ515634 | 2019 | Netherlands | A.D |
| A | MZ515641 | 2018 | Netherlands | A.D |
| A | MZ515650 | 2019 | Netherlands | A.D |
| A | MZ515681 | 2020 | United Kingdom | A.D |
| A | MZ515718 | 2019 | Netherlands | A.D |
| A | MZ515800 | 2018 | United Kingdom | A.D |
| A | MZ515802 | 2019 | United Kingdom | A.D |
| A | MZ515840 | 2017 | Netherlands | A.D |
| A | MZ515842 | 2020 | Spain | A.D |
| A | MZ515866 | 2018 | Netherlands | A.D |
| A | MZ515967 | 2018 | Netherlands | A.D |
| A | MZ515973 | 2017 | Netherlands | A.D |
| A | MZ516043 | 2017 | Netherlands | A.D |
| A | MZ516057 | 2019 | Netherlands | A.D |
| A | MZ516076 | 2018 | United Kingdom | A.D |
| A | MZ516077 | 2017 | Netherlands | A.D |
| A | OK649681 | 2012 | USA | A.D |
| A | OK649682 | 2012 | USA | A.D |
| A | OK649683 | 2014 | USA | A.D |
| A | OK649684 | 2015 | USA | A.D |
| A | ON237219 | 2014 | Argentina | A.D |
| A | ON237220 | 2014 | Argentina | A.D |
| A | ON237222 | 2014 | Argentina | A.D |
| A | ON237225 | 2014 | Argentina | A.D |
| A | ON237228 | 2014 | Argentina | A.D |
| A | ON237230 | 2014 | Argentina | A.D |
| A | ON237231 | 2014 | Argentina | A.D |
| A | ON237232 | 2014 | Argentina | A.D |
| A | ON237234 | 2014 | Argentina | A.D |
| A | ON237235 | 2014 | Argentina | A.D |
| A | ON237242 | 2014 | Argentina | A.D |
| A | ON237243 | 2014 | Argentina | A.D |
| A | ON237244 | 2014 | Argentina | A.D |
| A | ON237245 | 2014 | Argentina | A.D |
| A | ON237246 | 2015 | Argentina | A.D |
| A | ON237247 | 2015 | Argentina | A.D |
| A | ON237252 | 2015 | Argentina | A.D |
| A | ON237254 | 2015 | Argentina | A.D |
| A | ON237256 | 2015 | Argentina | A.D |
| A | ON237258 | 2015 | Argentina | A.D |
| A | ON237259 | 2015 | Argentina | A.D |
| A | ON237260 | 2015 | Argentina | A.D |
| A | ON237261 | 2015 | Argentina | A.D |
| A | ON237264 | 2015 | Argentina | A.D |
| A | ON237265 | 2015 | Argentina | A.D |
| A | ON237266 | 2015 | Argentina | A.D |
| A | ON237268 | 2015 | Argentina | A.D |
| A | ON237269 | 2015 | Argentina | A.D |
| A | ON237270 | 2015 | Argentina | A.D |
| A | ON237274 | 2016 | Argentina | A.D |
| A | ON237276 | 2016 | Argentina | A.D |
| A | ON237277 | 2016 | Argentina | A.D |
| A | ON237279 | 2016 | Argentina | A.D |
| A | ON237285 | 2016 | Argentina | A.D |
| A | ON237298 | 2017 | Argentina | A.D |
| A | ON237306 | 2017 | Argentina | A.D |
| A | ON729319 | 2019 | USA | A.D |
| A | OQ261746 | 2019 | Austria | A.D |
| A | MZ515682 | 2019 | United Kingdom | A.D.4.1 |
| A | JN257693 | 2010 | Canada | A.D |
| A | JN257694 | 2011 | Canada | A.D |
| A | KP321974 | 2012 | Canada | A.D |
| A | KP321982 | 2011 | Canada | A.D |
| A | KP321989 | 2012 | Canada | A.D |
| A | KP321981 | 2011 | Canada | A.D |
| A | KP321985 | 2012 | Canada | A.D |
| A | KP321983 | 2012 | Canada | A.D |
| A | KP321977 | 2012 | Canada | A.D |
| A | KP321976 | 2011 | Canada | A.D |
| A | KP321978 | 2012 | Canada | A.D |
| A | KP321984 | 2012 | Canada | A.D |
| A | KF300975 | 2010 | Panama | A.D |
| A | KF301018 | 2012 | Panama | A.D |
| A | KF300973 | 2010 | Panama | A.D |
| A | MG062688 | 2011 | Mexico | A.D.2 |
| A | MG062683 | 2009 | Mexico | A.D |
| A | MG062685 | 2011 | Mexico | A.D |
| A | MG062686 | 2012 | Mexico | A.D |
| A | MG062687 | 2013 | Mexico | A.D |
| A | KP792375 | 2014 | Spain | A.D |
| A | KP792370 | 2012 | Spain | A.D |
| A | KF915242 | 2012 | Spain | A.D |
| A | KF915265 | 2013 | Spain | A.D |
| A | KM402618 | 2014 | Spain | A.D |
| A | KM402671 | 2014 | Spain | A.D |
| A | KM402621 | 2014 | Spain | A.D |
| A | KM402630 | 2014 | Spain | A.D |
| A | KM402633 | 2014 | Spain | A.D |
| A | MG793382 | 2015 | Spain | A.D.2.2 |
| A | MH129176 | 2016 | Spain | A.D.1 |
| A | MH129196 | 2017 | Spain | A.D.2.2 |
| A | MH129254 | 2018 | Spain | A.D.2.1 |
| A | MH129071 | 2014 | Spain | A.D.2.2 |
| A | KM508824 | 2013 | Paraguay | A.D |
| A | KM508823 | 2013 | Paraguay | A.D |
| A | KM508825 | 2013 | Paraguay | A.D |
| A | KJ627264 | 2012 | Peru | A.D |
| A | KU350812 | 2013 | Argentina | A.D |
| A | KU350778 | 2012 | Argentina | A.D |
| A | KU350801 | 2013 | Argentina | A.D |
| A | KU350830 | 2014 | Argentina | A.D |
| A | KU350795 | 2012 | Argentina | A.D |
| A | KU350833 | 2014 | Argentina | A.D |
| A | KU350805 | 2013 | Argentina | A.D |
| A | KU350786 | 2012 | Argentina | A.D |
| A | KY634256 | 2014 | Argentina | A.D |
| A | KY634263 | 2014 | Argentina | A.D |
| A | KY634260 | 2015 | Argentina | A.D |
| A | KY634254 | 2015 | Argentina | A.D.5 |
| A | KY634284 | 2015 | Argentina | A.D |
| A | KY634271 | 2015 | Argentina | A.D.2.2 |
| A | KY634276 | 2015 | Argentina | A.D |
| A | KY634302 | 2015 | Argentina | A.D |
| A | KY634251 | 2015 | Argentina | A.D |
| A | KT953244 | 2013 | Brazil | A.D |
| A | KT953283 | 2012 | Brazil | A.D |
| A | KT953280 | 2012 | Brazil | A.D |
| A | KY828390 | 2015 | Brazil | A.D |
| A | KY828423 | 2014 | Brazil | A.D |
| A | KY828389 | 2015 | Brazil | A.D |
| A | KY828412 | 2013 | Brazil | A.D |
| A | KY828405 | 2013 | Brazil | A.D |
| A | KY828402 | 2013 | Brazil | A.D |
| A | KY828427 | 2014 | Brazil | A.D |
| A | KX894803 | 2013 | USA | A.D |
| A | KM042392 | 2013 | USA | A.D |
| A | KU950677 | 2012 | USA | A.D |
| A | KU950592 | 2012 | USA | A.D |
| A | KJ890469 | 2012 | USA | A.D |
| A | KJ672480 | 2013 | USA | A.D |
| A | KJ672428 | 2013 | USA | A.D |
| A | KU950523 | 2014 | USA | A.D.2.2 |
| A | KU839637 | 2014 | USA | A.D |
| A | JX627336 | 2011 | Korea | A.D |
| A | AB860240 | 2012 | Korea | A.D |
| A | MK634184 | 2012 | Korea | A.D |
| A | MK634202 | 2014 | Korea | A.D |
| A | MK634282 | 2017 | Korea | A.D |
| A | MK634281 | 2017 | Korea | A.D |
| A | MK634267 | 2017 | Korea | A.D |
| A | MK634265 | 2016 | Korea | A.D |
| A | MK634256 | 2016 | Korea | A.D |
| A | MK634240 | 2015 | Korea | A.D |
| A | MK634227 | 2015 | Korea | A.D |
| A | KC754738 | 2012 | Cuba | A.D |
| A | KC677857 | 2012 | Cuba | A.D |
| A | KC677877 | 2012 | Cuba | A.D |
| A | KC677862 | 2012 | Cuba | A.D |
| A | KC677870 | 2012 | Cuba | A.D |
| A | KC754743 | 2013 | Cuba | A.D |
| A | KC283044 | 2012 | Cuba | A.D |
| A | MN122449 | 2012 | Portugal | A.D |
| A | MN122464 | 2016 | Portugal | A.D |
| A | MN122473 | 2017 | Portugal | A.D |
| A | MN122504 | 2015 | Portugal | A.D.2.2 |
| A | MN122555 | 2018 | Portugal | A.D.5 |
| A | KJ710405 | 2012 | Germany | A.D |
| A | KJ710364 | 2013 | Germany | A.D |
| A | KJ710400 | 2013 | Germany | A.D |
| A | KJ710392 | 2013 | Germany | A.D |
| A | KJ710377 | 2013 | Germany | A.D |
| A | KJ710387 | 2013 | Germany | A.D |
| A | KJ710389 | 2012 | Germany | A.D |
| A | KJ710375 | 2013 | Germany | A.D |
| A | JX912355 | 2012 | Germany | A.D |
| A | JX912364 | 2012 | Germany | A.D |
| A | MZ397605 | 2015 | Germany | A.D |
| A | MZ397665 | 2016 | Germany | A.D |
| A | MZ397667 | 2017 | Germany | A.D.1 |
| A | AB698559 | 2012 | Japan | A.D |
| A | AB761609 | 2012 | Japan | A.D |
| A | AB754590 | 2012 | Japan | A.D |
| A | AB808780 | 2012 | Japan | A.D |
| A | AB761611 | 2012 | Japan | A.D |
| A | AB808772 | 2012 | Japan | A.D |
| A | AB808778 | 2012 | Japan | A.D |
| A | LC037686 | 2015 | Japan | A.D |
| A | LC037881 | 2014 | Japan | A.D |
| A | LC037477 | 2015 | Japan | A.D |
| A | KF057865 | 2012 | Croatia | A.D |
| A | KF057866 | 2013 | Croatia | A.D |
| A | KF057867 | 2013 | Croatia | A.D |
| A | KT371691 | 2014 | Croatia | A.D |
| A | KT371699 | 2014 | Croatia | A.D |
| A | KC342406 | 2011 | Thailand | A.D |
| A | KC342413 | 2011 | Thailand | A.D |
| A | KC342419 | 2011 | Thailand | A.D |
| A | KC342434 | 2011 | Thailand | A.D |
| A | KC342441 | 2011 | Thailand | A.D |
| A | KY327994 | 2013 | Thailand | A.D |
| A | KY328031 | 2014 | Thailand | A.D |
| A | KY328024 | 2014 | Thailand | A.D |
| A | KY328045 | 2015 | Thailand | A.D |
| A | KY328016 | 2015 | Thailand | A.D |
| A | MW678417 | 2020 | Thailand | A.D |
| A | MW678437 | 2020 | Thailand | A.D.3 |
| A | MW678563 | 2020 | Thailand | A.D.3 |
| A | MW678377 | 2019 | Thailand | A.D.3 |
| A | MW678283 | 2018 | Thailand | A.D.3 |
| A | MW678313 | 2018 | Thailand | A.D.3 |
| A | KF587967 | 2012 | Kenya | A.D |
| A | KX453496 | 2015 | Kenya | A.D |
| A | KX453422 | 2014 | Kenya | A.D.4 |
| A | KX453391 | 2014 | Kenya | A.D.4 |
| A | KX453321 | 2013 | Kenya | A.D |
| A | KX453530 | 2015 | Kenya | A.D.4 |
| A | AB846656 | 2013 | Philippines | A.D |
| A | AB846657 | 2013 | Philippines | A.D |
| A | AB846655 | 2012 | Philippines | A.D |
| A | KC476744 | 2012 | South Africa | A.D |
| A | KC476745 | 2012 | South Africa | A.D |
| A | JX885730 | 2012 | South Africa | A.D |
| A | JX885737 | 2012 | South Africa | A.D |
| A | JX885732 | 2012 | South Africa | A.D |
| A | KF030154 | 2012 | Latvia | A.D |
| A | JX256871 | 2011 | Malaysia | A.D |
| A | JX988439 | 2012 | Italy | A.D |
| A | JX988452 | 2012 | Italy | A.D |
| A | JX988445 | 2012 | Italy | A.D |
| A | KC858199 | 2012 | Italy | A.D |
| A | KC858212 | 2012 | Italy | A.D |
| A | KC858233 | 2013 | Italy | A.D |
| A | KC858254 | 2012 | Italy | A.D |
| A | MT156419 | 2017 | Italy | A.D.1 |
| A | MT156414 | 2017 | Italy | A.D.2.2 |
| A | MT156432 | 2018 | Italy | A.D.2.2 |
| A | KC731482 | 2011 | India | A.D |
| A | KF246639 | 2012 | India | A.D |
| A | KF246640 | 2012 | India | A.D |
| A | KF246638 | 2012 | India | A.D |
| A | KY030830 | 2014 | India | A.D.2.2 |
| A | KY030826 | 2012 | India | A.D |
| A | KY078405 | 2014 | India | A.D.2.2 |
| A | MG971431 | 2015 | Netherlands | A.D |
| A | MG971433 | 2016 | Netherlands | A.D |
| A | MG971421 | 2012 | Netherlands | A.D |
| A | MG971429 | 2015 | Netherlands | A.D |
| A | MG971424 | 2013 | Netherlands | A.D |
| A | KX858754 | 2013 | Netherlands | A.D |
| A | MG971459 | 2015 | Gambia | A.D.2.2 |
| A | MG971449 | 2015 | Gambia | A.D |
| A | MH760648 | 2015 | Australia | A.D |
| A | MH760647 | 2015 | Australia | A.D |
| A | MH760640 | 2014 | Australia | A.D.2.2 |
| A | MH760629 | 2016 | Australia | A.D |
| A | MH760624 | 2016 | Australia | A.D.2.2 |
| A | MH760611 | 2016 | Australia | A.D.2.2 |
| A | MH760607 | 2013 | Australia | A.D |
| A | MH760602 | 2012 | Australia | A.D |
| A | MH760623 | 2016 | Australia | A.D |
| A | MH760650 | 2015 | Australia | A.D |
| A | MH760628 | 2016 | Australia | A.D.2.2 |
| A | MH760636 | 2016 | Australia | A.D |
| A | MH760642 | 2014 | Australia | A.D |
| A | MH760606 | 2013 | Australia | A.D |
| A | KX765902 | 2014 | New Zealand | A.D |
| A | KX765941 | 2015 | New Zealand | A.D |
| A | KX765970 | 2014 | New Zealand | A.D |
| A | KX765939 | 2013 | New Zealand | A.D |
| A | KX765932 | 2015 | New Zealand | A.D |
| A | KX765926 | 2012 | New Zealand | A.D |
| A | KX765925 | 2015 | New Zealand | A.D |
| A | KX655644 | 2012 | Jordan | A.D |
| A | KX655626 | 2013 | Jordan | A.D |
| A | KU726088 | 2014 | Saudi Arabia | A.D |
| A | KU726085 | 2014 | Saudi Arabia | A.D |
| A | MH388036 | 2015 | Saudi Arabia | A.D.5 |
| A | MH388041 | 2015 | Saudi Arabia | A.D.5 |
| A | MH388029 | 2014 | Saudi Arabia | A.D |
| A | MH388032 | 2016 | Saudi Arabia | A.D |
| A | MK182714 | 2012 | Saudi Arabia | A.D |
| A | MK182716 | 2012 | Saudi Arabia | A.D |
| A | MN434097 | 2017 | Saudi Arabia | A.D |
| A | MN434120 | 2017 | Saudi Arabia | A.D.5 |
| A | MN434108 | 2017 | Saudi Arabia | A.D.5 |
| A | MN434112 | 2017 | Saudi Arabia | A.D.5 |
| A | MN434106 | 2017 | Saudi Arabia | A.D.2.2 |
| A | MN434121 | 2017 | Saudi Arabia | A.D.2.2 |
| A | KC559440 | 2012 | China | A.D |
| A | KM586845 | 2013 | China | A.D |
| A | KJ130648 | 2012 | China | A.D |
| A | KJ130647 | 2013 | China | A.D |
| A | MF445755 | 2015 | China | A.D.3 |
| A | MF445774 | 2015 | China | A.D.3 |
| A | MW260584 | 2017 | China | A.D.3 |
| A | MW455131 | 2018 | China | A.D.3 |
| A | KU254615 | 2015 | China | A.D |
| A | KU254632 | 2015 | China | A.D |
| A | KX009656 | 2015 | China | A.D |
| A | KX009676 | 2015 | China | A.D |
| A | KX009686 | 2015 | China | A.D |
| A | KX533571 | 2015 | China | A.D |
| A | KX533580 | 2015 | China | A.D |
| A | KX533601 | 2015 | China | A.D |
| A | MF445749 | 2015 | China | A.D |
| A | MF445775 | 2015 | China | A.D |
| A | MN007037 | 2018 | China | A.D.3 |
| A | MN007046 | 2018 | China | A.D.3 |
| A | MN007067 | 2018 | China | A.D.3 |
| A | MN007072 | 2019 | China | A.D.5 |
| A | MN007076 | 2019 | China | A.D.3 |
| A | MN007081 | 2018 | China | A.D.3 |
| A | PP508183 | 2023 | Peru | A.D.5.2 |
| A | PP508181 | 2022 | Peru | A.D.3.2 |
| A | PP508185 | 2023 | Peru | A.D.5.2 |
| A | PP508188 | 2023 | Peru | A.D.5.2 |
| A | EPI_ISL_18262238 | 2023 | Peru | A.D.5.1 |
| A | EPI_ISL_19231446 | 2024 | Peru | A.D.3.3 |
| A | EPI_ISL_19427456 | 2024 | Peru | A.D.1 |
| A | EPI_ISL_19427461 | 2024 | Peru | A.D.5.3 |
| A | EPI_ISL_19462483 | 2024 | Peru | A.D.1.5 |
| A | EPI_ISL_19624048 | 2024 | Peru | A.D.3 |

**Table S4. Summary of recombination screening performed with RDP4**

| **Dataset** | **Sequences (n)** | **Length (bp)** | **RDP4 Methods** | **Multiple correction** | **P-value threshold** | **Recombination events** |
| --- | --- | --- | --- | --- | --- | --- |
| Dataset for ML phylogeny | 710 | 333 | RDP, GENECONV, BootScan, MaxChi, Chimaera, SiScan, 3Seq | Bonferroni correction | 0.05 | 0 |
| Dataset for tMRCA inference | 817 | 333 | RDP, GENECONV, BootScan, MaxChi, Chimaera, SiScan, 3Seq | Bonferroni correction | 0.05 | 0 |

The datasets comprising reference sequences and sequences generated in this study were screened for recombination in the 333-bp fragment of the second hypervariable region of the G gene using the RDP, GENECONV, BootScan, MaxChi, Chimaera, SiScan, and 3Seq methods implemented in RDP4. Statistical significance was assessed using a p-value threshold of 0.05, with Bonferroni correction and 1000 permutations. No statistically significant recombination events were detected in any of the datasets.

**Table S5. O-glycosylation sites of the A.D. lineages sequences of this study**

| **Seqname** | **Position** | **Score** | **Result** |
| --- | --- | --- | --- |
| PER51_03_2020 | T219 | 0.972 | POSITIVE |
| PER51_03_2020 | T220 | 0.940 | POSITIVE |
| PER51_03_2020 | T227 | 0.970 | POSITIVE |
| PER51_03_2020 | T228 | 0.948 | POSITIVE |
| PER51_03_2020 | T231 | 0.968 | POSITIVE |
| PER51_03_2020 | T235 | 0.838 | POSITIVE |
| PER51_03_2020 | T238 | 0.737 | POSITIVE |
| PER51_03_2020 | T239 | 0.589 | POSITIVE |
| PER51_03_2020 | T241 | 0.399 |  |
| PER51_03_2020 | T245 | 0.648 | POSITIVE |
| PER51_03_2020 | T246 | 0.473 |  |
| PER51_03_2020 | T249 | 0.547 | POSITIVE |
| PER51_03_2020 | T252 | 0.609 | POSITIVE |
| PER51_03_2020 | T259 | 0.545 | POSITIVE |
| PER51_03_2020 | S260 | 0.541 | POSITIVE |
| PER51_03_2020 | T264 | 0.635 | POSITIVE |
| PER51_03_2020 | T268 | 0.508 | POSITIVE |
| PER51_03_2020 | T269 | 0.739 | POSITIVE |
| PER51_03_2020 | S270 | 0.866 | POSITIVE |
| PER51_03_2020 | S275 | 0.856 | POSITIVE |
| PER51_03_2020 | S277 | 0.884 | POSITIVE |
| PER51_03_2020 | T281 | 0.807 | POSITIVE |
| PER51_03_2020 | T282 | 0.722 | POSITIVE |
| PER51_03_2020 | S283 | 0.834 | POSITIVE |
| PER51_03_2020 | T288 | 0.760 | POSITIVE |
| PER51_03_2020 | S291 | 0.910 | POSITIVE |
| PER51_03_2020 | T292 | 0.802 | POSITIVE |
| PER51_03_2020 | T293 | 0.766 | POSITIVE |
| PER51_03_2020 | S294 | 0.885 | POSITIVE |
| PER51_03_2020 | S299 | 0.903 | POSITIVE |
| PER51_03_2020 | S301 | 0.688 | POSITIVE |
| PER51_03_2020 | T305 | 0.540 | POSITIVE |
| PER51_03_2020 | T306 | 0.429 |  |
| PER51_03_2020 | S307 | 0.662 | POSITIVE |
| PER51_03_2020 | S311 | 0.538 | POSITIVE |
| PER51_03_2020 | S313 | 0.588 | POSITIVE |
| PER51_03_2020 | S315 | 0.447 |  |
| PER51_03_2020 | S316 | 0.353 |  |
| PER51_03_2020 | S317 | 0.367 |  |
| PER51_03_2020 | T319 | 0.089 |  |
| PER2_06_2018 | T219 | 0.973 | POSITIVE |
| PER2_06_2018 | T220 | 0.944 | POSITIVE |
| PER2_06_2018 | T227 | 0.970 | POSITIVE |
| PER2_06_2018 | T228 | 0.951 | POSITIVE |
| PER2_06_2018 | T231 | 0.973 | POSITIVE |
| PER2_06_2018 | T235 | 0.899 | POSITIVE |
| PER2_06_2018 | T238 | 0.803 | POSITIVE |
| PER2_06_2018 | T239 | 0.704 | POSITIVE |
| PER2_06_2018 | T241 | 0.638 | POSITIVE |
| PER2_06_2018 | S243 | 0.784 | POSITIVE |
| PER2_06_2018 | T245 | 0.780 | POSITIVE |
| PER2_06_2018 | T246 | 0.642 | POSITIVE |
| PER2_06_2018 | T249 | 0.831 | POSITIVE |
| PER2_06_2018 | S250 | 0.554 | POSITIVE |
| PER2_06_2018 | T252 | 0.688 | POSITIVE |
| PER2_06_2018 | T259 | 0.800 | POSITIVE |
| PER2_06_2018 | S260 | 0.753 | POSITIVE |
| PER2_06_2018 | T264 | 0.835 | POSITIVE |
| PER2_06_2018 | S267 | 0.782 | POSITIVE |
| PER2_06_2018 | T268 | 0.775 | POSITIVE |
| PER2_06_2018 | T269 | 0.765 | POSITIVE |
| PER2_06_2018 | S270 | 0.803 | POSITIVE |
| PER2_06_2018 | S275 | 0.671 | POSITIVE |
| PER2_06_2018 | S277 | 0.881 | POSITIVE |
| PER2_06_2018 | T281 | 0.812 | POSITIVE |
| PER2_06_2018 | T282 | 0.700 | POSITIVE |
| PER2_06_2018 | S283 | 0.807 | POSITIVE |
| PER2_06_2018 | T288 | 0.751 | POSITIVE |
| PER2_06_2018 | S291 | 0.905 | POSITIVE |
| PER2_06_2018 | T292 | 0.768 | POSITIVE |
| PER2_06_2018 | T293 | 0.722 | POSITIVE |
| PER2_06_2018 | S294 | 0.851 | POSITIVE |
| PER2_06_2018 | S299 | 0.877 | POSITIVE |
| PER2_06_2018 | S301 | 0.650 | POSITIVE |
| PER2_06_2018 | T305 | 0.436 |  |
| PER2_06_2018 | T306 | 0.354 |  |
| PER2_06_2018 | S307 | 0.438 |  |
| PER2_06_2018 | S311 | 0.583 | POSITIVE |
| PER2_06_2018 | S313 | 0.593 | POSITIVE |
| PER2_06_2018 | S315 | 0.480 |  |
| PER2_06_2018 | S316 | 0.441 |  |
| PER2_06_2018 | S317 | 0.392 |  |
| PER2_06_2018 | T319 | 0.132 |  |
| PER2_06_2018 | T320 | 0.069 |  |
| PER44_09_2019 | T219 | 0.968 | POSITIVE |
| PER44_09_2019 | T220 | 0.941 | POSITIVE |
| PER44_09_2019 | T227 | 0.969 | POSITIVE |
| PER44_09_2019 | T228 | 0.947 | POSITIVE |
| PER44_09_2019 | T231 | 0.967 | POSITIVE |
| PER44_09_2019 | T235 | 0.815 | POSITIVE |
| PER44_09_2019 | T238 | 0.710 | POSITIVE |
| PER44_09_2019 | T239 | 0.549 | POSITIVE |
| PER44_09_2019 | T241 | 0.363 |  |
| PER44_09_2019 | T245 | 0.603 | POSITIVE |
| PER44_09_2019 | T246 | 0.444 |  |
| PER44_09_2019 | T249 | 0.527 | POSITIVE |
| PER44_09_2019 | T252 | 0.598 | POSITIVE |
| PER44_09_2019 | T259 | 0.548 | POSITIVE |
| PER44_09_2019 | S260 | 0.550 | POSITIVE |
| PER44_09_2019 | T264 | 0.629 | POSITIVE |
| PER44_09_2019 | T268 | 0.494 |  |
| PER44_09_2019 | T269 | 0.755 | POSITIVE |
| PER44_09_2019 | S270 | 0.864 | POSITIVE |
| PER44_09_2019 | S275 | 0.805 | POSITIVE |
| PER44_09_2019 | S277 | 0.785 | POSITIVE |
| PER44_09_2019 | T281 | 0.637 | POSITIVE |
| PER44_09_2019 | T282 | 0.500 | POSITIVE |
| PER44_09_2019 | S283 | 0.553 | POSITIVE |
| PER44_09_2019 | T288 | 0.481 |  |
| PER44_09_2019 | S291 | 0.776 | POSITIVE |
| PER44_09_2019 | T292 | 0.407 |  |
| PER44_09_2019 | S294 | 0.628 | POSITIVE |
| PER44_09_2019 | S299 | 0.678 | POSITIVE |
| PER44_09_2019 | S301 | 0.448 |  |
| PER44_09_2019 | T305 | 0.401 |  |
| PER44_09_2019 | T306 | 0.290 |  |
| PER44_09_2019 | S307 | 0.545 | POSITIVE |
| PER44_09_2019 | S311 | 0.490 |  |
| PER44_09_2019 | S313 | 0.511 | POSITIVE |
| PER44_09_2019 | S315 | 0.369 |  |
| PER44_09_2019 | S316 | 0.263 |  |
| PER44_09_2019 | S317 | 0.286 |  |
| PER44_09_2019 | T319 | 0.069 |  |
| PER36_07_2019 | T219 | 0.966 | POSITIVE |
| PER36_07_2019 | T220 | 0.939 | POSITIVE |
| PER36_07_2019 | T227 | 0.969 | POSITIVE |
| PER36_07_2019 | T228 | 0.948 | POSITIVE |
| PER36_07_2019 | T231 | 0.967 | POSITIVE |
| PER36_07_2019 | T235 | 0.822 | POSITIVE |
| PER36_07_2019 | T238 | 0.722 | POSITIVE |
| PER36_07_2019 | T239 | 0.567 | POSITIVE |
| PER36_07_2019 | T241 | 0.368 |  |
| PER36_07_2019 | T245 | 0.612 | POSITIVE |
| PER36_07_2019 | T246 | 0.454 |  |
| PER36_07_2019 | T249 | 0.545 | POSITIVE |
| PER36_07_2019 | T252 | 0.620 | POSITIVE |
| PER36_07_2019 | T259 | 0.634 | POSITIVE |
| PER36_07_2019 | S260 | 0.607 | POSITIVE |
| PER36_07_2019 | T264 | 0.767 | POSITIVE |
| PER36_07_2019 | S267 | 0.771 | POSITIVE |
| PER36_07_2019 | T268 | 0.705 | POSITIVE |
| PER36_07_2019 | T269 | 0.797 | POSITIVE |
| PER36_07_2019 | S270 | 0.874 | POSITIVE |
| PER36_07_2019 | S275 | 0.806 | POSITIVE |
| PER36_07_2019 | S277 | 0.826 | POSITIVE |
| PER36_07_2019 | T281 | 0.653 | POSITIVE |
| PER36_07_2019 | T282 | 0.509 | POSITIVE |
| PER36_07_2019 | S283 | 0.554 | POSITIVE |
| PER36_07_2019 | T288 | 0.477 |  |
| PER36_07_2019 | S291 | 0.770 | POSITIVE |
| PER36_07_2019 | T292 | 0.398 |  |
| PER36_07_2019 | S294 | 0.605 | POSITIVE |
| PER36_07_2019 | S299 | 0.654 | POSITIVE |
| PER36_07_2019 | S301 | 0.416 |  |
| PER36_07_2019 | T305 | 0.363 |  |
| PER36_07_2019 | T306 | 0.277 |  |
| PER36_07_2019 | S307 | 0.511 | POSITIVE |
| PER36_07_2019 | S311 | 0.458 |  |
| PER36_07_2019 | S313 | 0.466 |  |
| PER36_07_2019 | S315 | 0.362 |  |
| PER36_07_2019 | S316 | 0.258 |  |
| PER36_07_2019 | S317 | 0.272 |  |
| PER36_07_2019 | T319 | 0.063 |  |
| PER21_06_2018 | T219 | 0.973 | POSITIVE |
| PER21_06_2018 | T220 | 0.944 | POSITIVE |
| PER21_06_2018 | T227 | 0.970 | POSITIVE |
| PER21_06_2018 | T228 | 0.951 | POSITIVE |
| PER21_06_2018 | T231 | 0.973 | POSITIVE |
| PER21_06_2018 | T235 | 0.899 | POSITIVE |
| PER21_06_2018 | T238 | 0.803 | POSITIVE |
| PER21_06_2018 | T239 | 0.704 | POSITIVE |
| PER21_06_2018 | T241 | 0.638 | POSITIVE |
| PER21_06_2018 | S243 | 0.784 | POSITIVE |
| PER21_06_2018 | T245 | 0.780 | POSITIVE |
| PER21_06_2018 | T246 | 0.642 | POSITIVE |
| PER21_06_2018 | T249 | 0.831 | POSITIVE |
| PER21_06_2018 | S250 | 0.554 | POSITIVE |
| PER21_06_2018 | T252 | 0.688 | POSITIVE |
| PER21_06_2018 | T259 | 0.800 | POSITIVE |
| PER21_06_2018 | S260 | 0.753 | POSITIVE |
| PER21_06_2018 | T264 | 0.835 | POSITIVE |
| PER21_06_2018 | S267 | 0.782 | POSITIVE |
| PER21_06_2018 | T268 | 0.775 | POSITIVE |
| PER21_06_2018 | T269 | 0.765 | POSITIVE |
| PER21_06_2018 | S270 | 0.803 | POSITIVE |
| PER21_06_2018 | S275 | 0.671 | POSITIVE |
| PER21_06_2018 | S277 | 0.881 | POSITIVE |
| PER21_06_2018 | T281 | 0.812 | POSITIVE |
| PER21_06_2018 | T282 | 0.700 | POSITIVE |
| PER21_06_2018 | S283 | 0.807 | POSITIVE |
| PER21_06_2018 | T288 | 0.751 | POSITIVE |
| PER21_06_2018 | S291 | 0.905 | POSITIVE |
| PER21_06_2018 | T292 | 0.768 | POSITIVE |
| PER21_06_2018 | T293 | 0.722 | POSITIVE |
| PER21_06_2018 | S294 | 0.851 | POSITIVE |
| PER21_06_2018 | S299 | 0.877 | POSITIVE |
| PER21_06_2018 | S301 | 0.650 | POSITIVE |
| PER21_06_2018 | T305 | 0.436 |  |
| PER21_06_2018 | T306 | 0.354 |  |
| PER21_06_2018 | S307 | 0.438 |  |
| PER21_06_2018 | S311 | 0.583 | POSITIVE |
| PER21_06_2018 | S313 | 0.593 | POSITIVE |
| PER21_06_2018 | S315 | 0.480 |  |
| PER21_06_2018 | S316 | 0.441 |  |
| PER21_06_2018 | S317 | 0.392 |  |
| PER21_06_2018 | T319 | 0.132 |  |
| PER21_06_2018 | T320 | 0.069 |  |
| PER52_10_2019 | T219 | 0.974 | POSITIVE |
| PER52_10_2019 | T220 | 0.946 | POSITIVE |
| PER52_10_2019 | T227 | 0.962 | POSITIVE |
| PER52_10_2019 | T228 | 0.938 | POSITIVE |
| PER52_10_2019 | T231 | 0.939 | POSITIVE |
| PER52_10_2019 | T235 | 0.688 | POSITIVE |
| PER52_10_2019 | T238 | 0.529 | POSITIVE |
| PER52_10_2019 | T241 | 0.230 |  |
| PER52_10_2019 | T245 | 0.567 | POSITIVE |
| PER52_10_2019 | T246 | 0.357 |  |
| PER52_10_2019 | T249 | 0.611 | POSITIVE |
| PER52_10_2019 | S250 | 0.500 | POSITIVE |
| PER52_10_2019 | T252 | 0.668 | POSITIVE |
| PER52_10_2019 | T259 | 0.746 | POSITIVE |
| PER52_10_2019 | S260 | 0.664 | POSITIVE |
| PER52_10_2019 | T264 | 0.797 | POSITIVE |
| PER52_10_2019 | S267 | 0.769 | POSITIVE |
| PER52_10_2019 | T268 | 0.717 | POSITIVE |
| PER52_10_2019 | T269 | 0.803 | POSITIVE |
| PER52_10_2019 | S270 | 0.879 | POSITIVE |
| PER52_10_2019 | S272 | 0.652 | POSITIVE |
| PER52_10_2019 | S275 | 0.783 | POSITIVE |
| PER52_10_2019 | S277 | 0.689 | POSITIVE |
| PER52_10_2019 | T281 | 0.681 | POSITIVE |
| PER52_10_2019 | T282 | 0.472 |  |
| PER52_10_2019 | S283 | 0.481 |  |
| PER52_10_2019 | T288 | 0.396 |  |
| PER52_10_2019 | S291 | 0.712 | POSITIVE |
| PER52_10_2019 | T292 | 0.535 | POSITIVE |
| PER52_10_2019 | T293 | 0.329 |  |
| PER52_10_2019 | S294 | 0.519 | POSITIVE |
| PER52_10_2019 | S299 | 0.835 | POSITIVE |
| PER52_10_2019 | S301 | 0.791 | POSITIVE |
| PER52_10_2019 | T305 | 0.744 | POSITIVE |
| PER52_10_2019 | T306 | 0.567 | POSITIVE |
| PER52_10_2019 | S307 | 0.872 | POSITIVE |
| PER52_10_2019 | S311 | 0.810 | POSITIVE |
| PER52_10_2019 | S313 | 0.789 | POSITIVE |
| PER52_10_2019 | S315 | 0.564 | POSITIVE |
| PER52_10_2019 | S316 | 0.469 |  |
| PER52_10_2019 | S317 | 0.444 |  |
| PER52_10_2019 | T319 | 0.136 |  |
| PER24_07_2018 | T219 | 0.973 | POSITIVE |
| PER24_07_2018 | T220 | 0.944 | POSITIVE |
| PER24_07_2018 | T227 | 0.970 | POSITIVE |
| PER24_07_2018 | T228 | 0.951 | POSITIVE |
| PER24_07_2018 | T231 | 0.973 | POSITIVE |
| PER24_07_2018 | T235 | 0.899 | POSITIVE |
| PER24_07_2018 | T238 | 0.803 | POSITIVE |
| PER24_07_2018 | T239 | 0.704 | POSITIVE |
| PER24_07_2018 | T241 | 0.638 | POSITIVE |
| PER24_07_2018 | S243 | 0.784 | POSITIVE |
| PER24_07_2018 | T245 | 0.780 | POSITIVE |
| PER24_07_2018 | T246 | 0.642 | POSITIVE |
| PER24_07_2018 | T249 | 0.831 | POSITIVE |
| PER24_07_2018 | S250 | 0.554 | POSITIVE |
| PER24_07_2018 | T252 | 0.688 | POSITIVE |
| PER24_07_2018 | T259 | 0.800 | POSITIVE |
| PER24_07_2018 | S260 | 0.753 | POSITIVE |
| PER24_07_2018 | T264 | 0.835 | POSITIVE |
| PER24_07_2018 | S267 | 0.782 | POSITIVE |
| PER24_07_2018 | T268 | 0.775 | POSITIVE |
| PER24_07_2018 | T269 | 0.765 | POSITIVE |
| PER24_07_2018 | S270 | 0.803 | POSITIVE |
| PER24_07_2018 | S275 | 0.671 | POSITIVE |
| PER24_07_2018 | S277 | 0.881 | POSITIVE |
| PER24_07_2018 | T281 | 0.812 | POSITIVE |
| PER24_07_2018 | T282 | 0.700 | POSITIVE |
| PER24_07_2018 | S283 | 0.807 | POSITIVE |
| PER24_07_2018 | T288 | 0.751 | POSITIVE |
| PER24_07_2018 | S291 | 0.905 | POSITIVE |
| PER24_07_2018 | T292 | 0.768 | POSITIVE |
| PER24_07_2018 | T293 | 0.722 | POSITIVE |
| PER24_07_2018 | S294 | 0.851 | POSITIVE |
| PER24_07_2018 | S299 | 0.877 | POSITIVE |
| PER24_07_2018 | S301 | 0.650 | POSITIVE |
| PER24_07_2018 | T305 | 0.436 |  |
| PER24_07_2018 | T306 | 0.354 |  |
| PER24_07_2018 | S307 | 0.438 |  |
| PER24_07_2018 | S311 | 0.583 | POSITIVE |
| PER24_07_2018 | S313 | 0.593 | POSITIVE |
| PER24_07_2018 | S315 | 0.480 |  |
| PER24_07_2018 | S316 | 0.441 |  |
| PER24_07_2018 | S317 | 0.392 |  |
| PER24_07_2018 | T319 | 0.132 |  |
| PER24_07_2018 | T320 | 0.069 |  |
| PER48_09_2019 | T219 | 0.968 | POSITIVE |
| PER48_09_2019 | T220 | 0.941 | POSITIVE |
| PER48_09_2019 | T227 | 0.969 | POSITIVE |
| PER48_09_2019 | T228 | 0.947 | POSITIVE |
| PER48_09_2019 | T231 | 0.967 | POSITIVE |
| PER48_09_2019 | T235 | 0.815 | POSITIVE |
| PER48_09_2019 | T238 | 0.710 | POSITIVE |
| PER48_09_2019 | T239 | 0.549 | POSITIVE |
| PER48_09_2019 | T241 | 0.363 |  |
| PER48_09_2019 | T245 | 0.603 | POSITIVE |
| PER48_09_2019 | T246 | 0.444 |  |
| PER48_09_2019 | T249 | 0.527 | POSITIVE |
| PER48_09_2019 | T252 | 0.598 | POSITIVE |
| PER48_09_2019 | T259 | 0.548 | POSITIVE |
| PER48_09_2019 | S260 | 0.550 | POSITIVE |
| PER48_09_2019 | T264 | 0.629 | POSITIVE |
| PER48_09_2019 | T268 | 0.494 |  |
| PER48_09_2019 | T269 | 0.755 | POSITIVE |
| PER48_09_2019 | S270 | 0.864 | POSITIVE |
| PER48_09_2019 | S275 | 0.805 | POSITIVE |
| PER48_09_2019 | S277 | 0.785 | POSITIVE |
| PER48_09_2019 | T281 | 0.637 | POSITIVE |
| PER48_09_2019 | T282 | 0.500 | POSITIVE |
| PER48_09_2019 | S283 | 0.553 | POSITIVE |
| PER48_09_2019 | T288 | 0.481 |  |
| PER48_09_2019 | S291 | 0.776 | POSITIVE |
| PER48_09_2019 | T292 | 0.407 |  |
| PER48_09_2019 | S294 | 0.628 | POSITIVE |
| PER48_09_2019 | S299 | 0.678 | POSITIVE |
| PER48_09_2019 | S301 | 0.448 |  |
| PER48_09_2019 | T305 | 0.401 |  |
| PER48_09_2019 | T306 | 0.290 |  |
| PER48_09_2019 | S307 | 0.545 | POSITIVE |
| PER48_09_2019 | S311 | 0.490 |  |
| PER48_09_2019 | S313 | 0.511 | POSITIVE |
| PER48_09_2019 | S315 | 0.369 |  |
| PER48_09_2019 | S316 | 0.263 |  |
| PER48_09_2019 | S317 | 0.286 |  |
| PER48_09_2019 | T319 | 0.069 |  |
| PER13_06_2010 | T219 | 0.966 | POSITIVE |
| PER13_06_2010 | T220 | 0.939 | POSITIVE |
| PER13_06_2010 | T227 | 0.969 | POSITIVE |
| PER13_06_2010 | T228 | 0.950 | POSITIVE |
| PER13_06_2010 | T231 | 0.968 | POSITIVE |
| PER13_06_2010 | T235 | 0.851 | POSITIVE |
| PER13_06_2010 | T238 | 0.771 | POSITIVE |
| PER13_06_2010 | T239 | 0.619 | POSITIVE |
| PER13_06_2010 | T241 | 0.455 |  |
| PER13_06_2010 | T245 | 0.696 | POSITIVE |
| PER13_06_2010 | T246 | 0.584 | POSITIVE |
| PER13_06_2010 | T249 | 0.714 | POSITIVE |
| PER13_06_2010 | S250 | 0.525 | POSITIVE |
| PER13_06_2010 | T252 | 0.620 | POSITIVE |
| PER13_06_2010 | T259 | 0.715 | POSITIVE |
| PER13_06_2010 | S260 | 0.722 | POSITIVE |
| PER13_06_2010 | T264 | 0.801 | POSITIVE |
| PER13_06_2010 | S267 | 0.805 | POSITIVE |
| PER13_06_2010 | T268 | 0.753 | POSITIVE |
| PER13_06_2010 | T269 | 0.856 | POSITIVE |
| PER13_06_2010 | S270 | 0.882 | POSITIVE |
| PER13_06_2010 | S275 | 0.844 | POSITIVE |
| PER13_06_2010 | S277 | 0.900 | POSITIVE |
| PER13_06_2010 | T281 | 0.792 | POSITIVE |
| PER13_06_2010 | T282 | 0.657 | POSITIVE |
| PER13_06_2010 | S283 | 0.784 | POSITIVE |
| PER13_06_2010 | T288 | 0.634 | POSITIVE |
| PER13_06_2010 | S291 | 0.839 | POSITIVE |
| PER13_06_2010 | T292 | 0.662 | POSITIVE |
| PER13_06_2010 | T293 | 0.583 | POSITIVE |
| PER13_06_2010 | S294 | 0.762 | POSITIVE |
| PER13_06_2010 | S299 | 0.808 | POSITIVE |
| PER13_06_2010 | S301 | 0.474 |  |
| PER13_06_2010 | T305 | 0.395 |  |
| PER13_06_2010 | T306 | 0.304 |  |
| PER13_06_2010 | S307 | 0.545 | POSITIVE |
| PER13_06_2010 | S311 | 0.450 |  |
| PER13_06_2010 | S313 | 0.471 |  |
| PER13_06_2010 | S315 | 0.367 |  |
| PER13_06_2010 | S316 | 0.254 |  |
| PER13_06_2010 | S317 | 0.276 |  |
| PER13_06_2010 | T319 | 0.065 |  |
| PER53_06_2020 | T219 | 0.966 | POSITIVE |
| PER53_06_2020 | T220 | 0.938 | POSITIVE |
| PER53_06_2020 | T227 | 0.968 | POSITIVE |
| PER53_06_2020 | T228 | 0.949 | POSITIVE |
| PER53_06_2020 | T231 | 0.968 | POSITIVE |
| PER53_06_2020 | T235 | 0.848 | POSITIVE |
| PER53_06_2020 | T238 | 0.768 | POSITIVE |
| PER53_06_2020 | T239 | 0.615 | POSITIVE |
| PER53_06_2020 | T241 | 0.450 |  |
| PER53_06_2020 | T245 | 0.691 | POSITIVE |
| PER53_06_2020 | T246 | 0.580 | POSITIVE |
| PER53_06_2020 | T249 | 0.711 | POSITIVE |
| PER53_06_2020 | S250 | 0.522 | POSITIVE |
| PER53_06_2020 | T252 | 0.620 | POSITIVE |
| PER53_06_2020 | T259 | 0.715 | POSITIVE |
| PER53_06_2020 | S260 | 0.718 | POSITIVE |
| PER53_06_2020 | T264 | 0.781 | POSITIVE |
| PER53_06_2020 | S267 | 0.787 | POSITIVE |
| PER53_06_2020 | T268 | 0.724 | POSITIVE |
| PER53_06_2020 | T269 | 0.808 | POSITIVE |
| PER53_06_2020 | S270 | 0.879 | POSITIVE |
| PER53_06_2020 | S275 | 0.806 | POSITIVE |
| PER53_06_2020 | S277 | 0.837 | POSITIVE |
| PER53_06_2020 | T281 | 0.723 | POSITIVE |
| PER53_06_2020 | T282 | 0.571 | POSITIVE |
| PER53_06_2020 | S283 | 0.712 | POSITIVE |
| PER53_06_2020 | T288 | 0.629 | POSITIVE |
| PER53_06_2020 | S291 | 0.835 | POSITIVE |
| PER53_06_2020 | T292 | 0.657 | POSITIVE |
| PER53_06_2020 | T293 | 0.577 | POSITIVE |
| PER53_06_2020 | S294 | 0.758 | POSITIVE |
| PER53_06_2020 | S299 | 0.804 | POSITIVE |
| PER53_06_2020 | S301 | 0.468 |  |
| PER53_06_2020 | T305 | 0.389 |  |
| PER53_06_2020 | T306 | 0.299 |  |
| PER53_06_2020 | S307 | 0.539 | POSITIVE |
| PER53_06_2020 | S311 | 0.446 |  |
| PER53_06_2020 | S313 | 0.467 |  |
| PER53_06_2020 | S315 | 0.363 |  |
| PER53_06_2020 | S316 | 0.252 |  |
| PER53_06_2020 | S317 | 0.274 |  |
| PER53_06_2020 | T319 | 0.065 |  |
| PER15_06_2018 | T219 | 0.966 | POSITIVE |
| PER15_06_2018 | T220 | 0.938 | POSITIVE |
| PER15_06_2018 | T227 | 0.968 | POSITIVE |
| PER15_06_2018 | T228 | 0.949 | POSITIVE |
| PER15_06_2018 | T231 | 0.968 | POSITIVE |
| PER15_06_2018 | T235 | 0.848 | POSITIVE |
| PER15_06_2018 | T238 | 0.768 | POSITIVE |
| PER15_06_2018 | T239 | 0.615 | POSITIVE |
| PER15_06_2018 | T241 | 0.450 |  |
| PER15_06_2018 | T245 | 0.691 | POSITIVE |
| PER15_06_2018 | T246 | 0.580 | POSITIVE |
| PER15_06_2018 | T249 | 0.711 | POSITIVE |
| PER15_06_2018 | S250 | 0.522 | POSITIVE |
| PER15_06_2018 | T252 | 0.620 | POSITIVE |
| PER15_06_2018 | T259 | 0.715 | POSITIVE |
| PER15_06_2018 | S260 | 0.718 | POSITIVE |
| PER15_06_2018 | T264 | 0.781 | POSITIVE |
| PER15_06_2018 | S267 | 0.787 | POSITIVE |
| PER15_06_2018 | T268 | 0.724 | POSITIVE |
| PER15_06_2018 | T269 | 0.808 | POSITIVE |
| PER15_06_2018 | S270 | 0.879 | POSITIVE |
| PER15_06_2018 | S275 | 0.806 | POSITIVE |
| PER15_06_2018 | S277 | 0.837 | POSITIVE |
| PER15_06_2018 | T281 | 0.723 | POSITIVE |
| PER15_06_2018 | T282 | 0.571 | POSITIVE |
| PER15_06_2018 | S283 | 0.712 | POSITIVE |
| PER15_06_2018 | T288 | 0.629 | POSITIVE |
| PER15_06_2018 | S291 | 0.835 | POSITIVE |
| PER15_06_2018 | T292 | 0.657 | POSITIVE |
| PER15_06_2018 | T293 | 0.577 | POSITIVE |
| PER15_06_2018 | S294 | 0.758 | POSITIVE |
| PER15_06_2018 | S299 | 0.804 | POSITIVE |
| PER15_06_2018 | S301 | 0.468 |  |
| PER15_06_2018 | T305 | 0.389 |  |
| PER15_06_2018 | T306 | 0.299 |  |
| PER15_06_2018 | S307 | 0.539 | POSITIVE |
| PER15_06_2018 | S311 | 0.446 |  |
| PER15_06_2018 | S313 | 0.467 |  |
| PER15_06_2018 | S315 | 0.363 |  |
| PER15_06_2018 | S316 | 0.252 |  |
| PER15_06_2018 | S317 | 0.274 |  |
| PER15_06_2018 | T319 | 0.065 |  |
| PER7_04_2010 | T219 | 0.966 | POSITIVE |
| PER7_04_2010 | T220 | 0.939 | POSITIVE |
| PER7_04_2010 | T227 | 0.972 | POSITIVE |
| PER7_04_2010 | T228 | 0.940 | POSITIVE |
| PER7_04_2010 | T231 | 0.958 | POSITIVE |
| PER7_04_2010 | T235 | 0.924 | POSITIVE |
| PER7_04_2010 | T239 | 0.703 | POSITIVE |
| PER7_04_2010 | T241 | 0.545 | POSITIVE |
| PER7_04_2010 | T245 | 0.668 | POSITIVE |
| PER7_04_2010 | T246 | 0.548 | POSITIVE |
| PER7_04_2010 | T249 | 0.730 | POSITIVE |
| PER7_04_2010 | S250 | 0.557 | POSITIVE |
| PER7_04_2010 | T252 | 0.639 | POSITIVE |
| PER7_04_2010 | T259 | 0.710 | POSITIVE |
| PER7_04_2010 | S260 | 0.718 | POSITIVE |
| PER7_04_2010 | T264 | 0.798 | POSITIVE |
| PER7_04_2010 | S267 | 0.802 | POSITIVE |
| PER7_04_2010 | T268 | 0.750 | POSITIVE |
| PER7_04_2010 | T269 | 0.854 | POSITIVE |
| PER7_04_2010 | S270 | 0.880 | POSITIVE |
| PER7_04_2010 | S275 | 0.841 | POSITIVE |
| PER7_04_2010 | S277 | 0.898 | POSITIVE |
| PER7_04_2010 | T281 | 0.788 | POSITIVE |
| PER7_04_2010 | T282 | 0.653 | POSITIVE |
| PER7_04_2010 | S283 | 0.781 | POSITIVE |
| PER7_04_2010 | T288 | 0.629 | POSITIVE |
| PER7_04_2010 | S291 | 0.835 | POSITIVE |
| PER7_04_2010 | T292 | 0.656 | POSITIVE |
| PER7_04_2010 | T293 | 0.576 | POSITIVE |
| PER7_04_2010 | S294 | 0.757 | POSITIVE |
| PER7_04_2010 | S299 | 0.804 | POSITIVE |
| PER7_04_2010 | S301 | 0.468 |  |
| PER7_04_2010 | T305 | 0.389 |  |
| PER7_04_2010 | T306 | 0.299 |  |
| PER7_04_2010 | S307 | 0.540 | POSITIVE |
| PER7_04_2010 | S311 | 0.446 |  |
| PER7_04_2010 | S313 | 0.468 |  |
| PER7_04_2010 | S315 | 0.364 |  |
| PER7_04_2010 | S316 | 0.252 |  |
| PER7_04_2010 | S317 | 0.274 |  |
| PER7_04_2010 | T319 | 0.065 |  |
| PER32_07_2019 | T219 | 0.966 | POSITIVE |
| PER32_07_2019 | T220 | 0.938 | POSITIVE |
| PER32_07_2019 | T227 | 0.968 | POSITIVE |
| PER32_07_2019 | T228 | 0.949 | POSITIVE |
| PER32_07_2019 | T231 | 0.968 | POSITIVE |
| PER32_07_2019 | T235 | 0.848 | POSITIVE |
| PER32_07_2019 | T238 | 0.768 | POSITIVE |
| PER32_07_2019 | T239 | 0.615 | POSITIVE |
| PER32_07_2019 | T241 | 0.450 |  |
| PER32_07_2019 | T245 | 0.691 | POSITIVE |
| PER32_07_2019 | T246 | 0.580 | POSITIVE |
| PER32_07_2019 | T249 | 0.711 | POSITIVE |
| PER32_07_2019 | S250 | 0.522 | POSITIVE |
| PER32_07_2019 | T252 | 0.620 | POSITIVE |
| PER32_07_2019 | T259 | 0.715 | POSITIVE |
| PER32_07_2019 | S260 | 0.718 | POSITIVE |
| PER32_07_2019 | T264 | 0.781 | POSITIVE |
| PER32_07_2019 | S267 | 0.787 | POSITIVE |
| PER32_07_2019 | T268 | 0.724 | POSITIVE |
| PER32_07_2019 | T269 | 0.808 | POSITIVE |
| PER32_07_2019 | S270 | 0.879 | POSITIVE |
| PER32_07_2019 | S275 | 0.806 | POSITIVE |
| PER32_07_2019 | S277 | 0.837 | POSITIVE |
| PER32_07_2019 | T281 | 0.723 | POSITIVE |
| PER32_07_2019 | T282 | 0.571 | POSITIVE |
| PER32_07_2019 | S283 | 0.712 | POSITIVE |
| PER32_07_2019 | T288 | 0.629 | POSITIVE |
| PER32_07_2019 | S291 | 0.835 | POSITIVE |
| PER32_07_2019 | T292 | 0.657 | POSITIVE |
| PER32_07_2019 | T293 | 0.577 | POSITIVE |
| PER32_07_2019 | S294 | 0.758 | POSITIVE |
| PER32_07_2019 | S299 | 0.804 | POSITIVE |
| PER32_07_2019 | S301 | 0.468 |  |
| PER32_07_2019 | T305 | 0.389 |  |
| PER32_07_2019 | T306 | 0.299 |  |
| PER32_07_2019 | S307 | 0.539 | POSITIVE |
| PER32_07_2019 | S311 | 0.446 |  |
| PER32_07_2019 | S313 | 0.467 |  |
| PER32_07_2019 | S315 | 0.363 |  |
| PER32_07_2019 | S316 | 0.252 |  |
| PER32_07_2019 | S317 | 0.274 |  |
| PER32_07_2019 | T319 | 0.065 |  |
| PER47_09_2019 | T219 | 0.968 | POSITIVE |
| PER47_09_2019 | T220 | 0.941 | POSITIVE |
| PER47_09_2019 | T227 | 0.969 | POSITIVE |
| PER47_09_2019 | T228 | 0.947 | POSITIVE |
| PER47_09_2019 | T231 | 0.967 | POSITIVE |
| PER47_09_2019 | T235 | 0.815 | POSITIVE |
| PER47_09_2019 | T238 | 0.710 | POSITIVE |
| PER47_09_2019 | T239 | 0.549 | POSITIVE |
| PER47_09_2019 | T241 | 0.363 |  |
| PER47_09_2019 | T245 | 0.603 | POSITIVE |
| PER47_09_2019 | T246 | 0.444 |  |
| PER47_09_2019 | T249 | 0.527 | POSITIVE |
| PER47_09_2019 | T252 | 0.598 | POSITIVE |
| PER47_09_2019 | T259 | 0.548 | POSITIVE |
| PER47_09_2019 | S260 | 0.550 | POSITIVE |
| PER47_09_2019 | T264 | 0.629 | POSITIVE |
| PER47_09_2019 | T268 | 0.494 |  |
| PER47_09_2019 | T269 | 0.755 | POSITIVE |
| PER47_09_2019 | S270 | 0.864 | POSITIVE |
| PER47_09_2019 | S275 | 0.805 | POSITIVE |
| PER47_09_2019 | S277 | 0.785 | POSITIVE |
| PER47_09_2019 | T281 | 0.637 | POSITIVE |
| PER47_09_2019 | T282 | 0.500 | POSITIVE |
| PER47_09_2019 | S283 | 0.553 | POSITIVE |
| PER47_09_2019 | T288 | 0.481 |  |
| PER47_09_2019 | S291 | 0.776 | POSITIVE |
| PER47_09_2019 | T292 | 0.407 |  |
| PER47_09_2019 | S294 | 0.628 | POSITIVE |
| PER47_09_2019 | S299 | 0.678 | POSITIVE |
| PER47_09_2019 | S301 | 0.448 |  |
| PER47_09_2019 | T305 | 0.401 |  |
| PER47_09_2019 | T306 | 0.290 |  |
| PER47_09_2019 | S307 | 0.545 | POSITIVE |
| PER47_09_2019 | S311 | 0.490 |  |
| PER47_09_2019 | S313 | 0.511 | POSITIVE |
| PER47_09_2019 | S315 | 0.369 |  |
| PER47_09_2019 | S316 | 0.263 |  |
| PER47_09_2019 | S317 | 0.286 |  |
| PER47_09_2019 | T319 | 0.069 |  |
| PER3_05_2009 | T219 | 0.970 | POSITIVE |
| PER3_05_2009 | T220 | 0.940 | POSITIVE |
| PER3_05_2009 | T227 | 0.970 | POSITIVE |
| PER3_05_2009 | T228 | 0.952 | POSITIVE |
| PER3_05_2009 | T231 | 0.970 | POSITIVE |
| PER3_05_2009 | T235 | 0.860 | POSITIVE |
| PER3_05_2009 | T238 | 0.785 | POSITIVE |
| PER3_05_2009 | T239 | 0.637 | POSITIVE |
| PER3_05_2009 | T241 | 0.475 |  |
| PER3_05_2009 | T245 | 0.712 | POSITIVE |
| PER3_05_2009 | T246 | 0.603 | POSITIVE |
| PER3_05_2009 | T249 | 0.730 | POSITIVE |
| PER3_05_2009 | S250 | 0.545 | POSITIVE |
| PER3_05_2009 | T252 | 0.641 | POSITIVE |
| PER3_05_2009 | T259 | 0.723 | POSITIVE |
| PER3_05_2009 | S260 | 0.723 | POSITIVE |
| PER3_05_2009 | T264 | 0.734 | POSITIVE |
| PER3_05_2009 | S267 | 0.762 | POSITIVE |
| PER3_05_2009 | T268 | 0.692 | POSITIVE |
| PER3_05_2009 | T269 | 0.775 | POSITIVE |
| PER3_05_2009 | S270 | 0.808 | POSITIVE |
| PER3_05_2009 | S275 | 0.533 | POSITIVE |
| PER3_05_2009 | S277 | 0.800 | POSITIVE |
| PER3_05_2009 | T281 | 0.671 | POSITIVE |
| PER3_05_2009 | T282 | 0.568 | POSITIVE |
| PER3_05_2009 | S283 | 0.629 | POSITIVE |
| PER3_05_2009 | S284 | 0.623 | POSITIVE |
| PER3_05_2009 | T288 | 0.561 | POSITIVE |
| PER3_05_2009 | S291 | 0.692 | POSITIVE |
| PER3_05_2009 | T292 | 0.477 |  |
| PER3_05_2009 | T293 | 0.429 |  |
| PER3_05_2009 | S294 | 0.492 |  |
| PER3_05_2009 | S299 | 0.411 |  |
| PER3_05_2009 | S301 | 0.290 |  |
| PER3_05_2009 | T305 | 0.199 |  |
| PER3_05_2009 | T306 | 0.168 |  |
| PER3_05_2009 | S307 | 0.208 |  |
| PER3_05_2009 | S311 | 0.393 |  |
| PER3_05_2009 | S313 | 0.405 |  |
| PER3_05_2009 | S315 | 0.369 |  |
| PER3_05_2009 | S316 | 0.317 |  |
| PER3_05_2009 | S317 | 0.286 |  |
| PER3_05_2009 | T319 | 0.095 |  |
| PER3_05_2009 | T320 | 0.050 |  |
| PER46_09_2019 | T219 | 0.968 | POSITIVE |
| PER46_09_2019 | T220 | 0.941 | POSITIVE |
| PER46_09_2019 | T227 | 0.969 | POSITIVE |
| PER46_09_2019 | T228 | 0.947 | POSITIVE |
| PER46_09_2019 | T231 | 0.967 | POSITIVE |
| PER46_09_2019 | T235 | 0.815 | POSITIVE |
| PER46_09_2019 | T238 | 0.710 | POSITIVE |
| PER46_09_2019 | T239 | 0.549 | POSITIVE |
| PER46_09_2019 | T241 | 0.363 |  |
| PER46_09_2019 | T245 | 0.603 | POSITIVE |
| PER46_09_2019 | T246 | 0.444 |  |
| PER46_09_2019 | T249 | 0.527 | POSITIVE |
| PER46_09_2019 | T252 | 0.598 | POSITIVE |
| PER46_09_2019 | T259 | 0.548 | POSITIVE |
| PER46_09_2019 | S260 | 0.550 | POSITIVE |
| PER46_09_2019 | T264 | 0.629 | POSITIVE |
| PER46_09_2019 | T268 | 0.494 |  |
| PER46_09_2019 | T269 | 0.755 | POSITIVE |
| PER46_09_2019 | S270 | 0.864 | POSITIVE |
| PER46_09_2019 | S275 | 0.805 | POSITIVE |
| PER46_09_2019 | S277 | 0.785 | POSITIVE |
| PER46_09_2019 | T281 | 0.637 | POSITIVE |
| PER46_09_2019 | T282 | 0.500 | POSITIVE |
| PER46_09_2019 | S283 | 0.553 | POSITIVE |
| PER46_09_2019 | T288 | 0.481 |  |
| PER46_09_2019 | S291 | 0.776 | POSITIVE |
| PER46_09_2019 | T292 | 0.407 |  |
| PER46_09_2019 | S294 | 0.628 | POSITIVE |
| PER46_09_2019 | S299 | 0.678 | POSITIVE |
| PER46_09_2019 | S301 | 0.448 |  |
| PER46_09_2019 | T305 | 0.401 |  |
| PER46_09_2019 | T306 | 0.290 |  |
| PER46_09_2019 | S307 | 0.545 | POSITIVE |
| PER46_09_2019 | S311 | 0.490 |  |
| PER46_09_2019 | S313 | 0.511 | POSITIVE |
| PER46_09_2019 | S315 | 0.369 |  |
| PER46_09_2019 | S316 | 0.263 |  |
| PER46_09_2019 | S317 | 0.286 |  |
| PER46_09_2019 | T319 | 0.069 |  |
| PER31_04_2019 | T219 | 0.965 | POSITIVE |
| PER31_04_2019 | T220 | 0.938 | POSITIVE |
| PER31_04_2019 | T227 | 0.970 | POSITIVE |
| PER31_04_2019 | T228 | 0.959 | POSITIVE |
| PER31_04_2019 | T231 | 0.979 | POSITIVE |
| PER31_04_2019 | T235 | 0.857 | POSITIVE |
| PER31_04_2019 | T238 | 0.815 | POSITIVE |
| PER31_04_2019 | T239 | 0.650 | POSITIVE |
| PER31_04_2019 | T241 | 0.491 |  |
| PER31_04_2019 | T245 | 0.680 | POSITIVE |
| PER31_04_2019 | T246 | 0.570 | POSITIVE |
| PER31_04_2019 | T249 | 0.707 | POSITIVE |
| PER31_04_2019 | S250 | 0.527 | POSITIVE |
| PER31_04_2019 | T252 | 0.619 | POSITIVE |
| PER31_04_2019 | T259 | 0.715 | POSITIVE |
| PER31_04_2019 | S260 | 0.718 | POSITIVE |
| PER31_04_2019 | T264 | 0.781 | POSITIVE |
| PER31_04_2019 | S267 | 0.787 | POSITIVE |
| PER31_04_2019 | T268 | 0.723 | POSITIVE |
| PER31_04_2019 | T269 | 0.808 | POSITIVE |
| PER31_04_2019 | S270 | 0.879 | POSITIVE |
| PER31_04_2019 | S275 | 0.806 | POSITIVE |
| PER31_04_2019 | S277 | 0.836 | POSITIVE |
| PER31_04_2019 | T281 | 0.723 | POSITIVE |
| PER31_04_2019 | T282 | 0.571 | POSITIVE |
| PER31_04_2019 | S283 | 0.711 | POSITIVE |
| PER31_04_2019 | T288 | 0.628 | POSITIVE |
| PER31_04_2019 | S291 | 0.835 | POSITIVE |
| PER31_04_2019 | T292 | 0.656 | POSITIVE |
| PER31_04_2019 | T293 | 0.576 | POSITIVE |
| PER31_04_2019 | S294 | 0.757 | POSITIVE |
| PER31_04_2019 | S299 | 0.804 | POSITIVE |
| PER31_04_2019 | S301 | 0.467 |  |
| PER31_04_2019 | T305 | 0.388 |  |
| PER31_04_2019 | T306 | 0.298 |  |
| PER31_04_2019 | S307 | 0.538 | POSITIVE |
| PER31_04_2019 | S311 | 0.445 |  |
| PER31_04_2019 | S313 | 0.467 |  |
| PER31_04_2019 | S315 | 0.363 |  |
| PER31_04_2019 | S316 | 0.252 |  |
| PER31_04_2019 | S317 | 0.274 |  |
| PER31_04_2019 | T319 | 0.065 |  |
| PER30_04_2019 | T219 | 0.966 | POSITIVE |
| PER30_04_2019 | T220 | 0.938 | POSITIVE |
| PER30_04_2019 | T227 | 0.968 | POSITIVE |
| PER30_04_2019 | T228 | 0.949 | POSITIVE |
| PER30_04_2019 | T231 | 0.968 | POSITIVE |
| PER30_04_2019 | T235 | 0.848 | POSITIVE |
| PER30_04_2019 | T238 | 0.768 | POSITIVE |
| PER30_04_2019 | T239 | 0.615 | POSITIVE |
| PER30_04_2019 | T241 | 0.450 |  |
| PER30_04_2019 | T245 | 0.691 | POSITIVE |
| PER30_04_2019 | T246 | 0.580 | POSITIVE |
| PER30_04_2019 | T249 | 0.711 | POSITIVE |
| PER30_04_2019 | S250 | 0.522 | POSITIVE |
| PER30_04_2019 | T252 | 0.620 | POSITIVE |
| PER30_04_2019 | T259 | 0.715 | POSITIVE |
| PER30_04_2019 | S260 | 0.718 | POSITIVE |
| PER30_04_2019 | T264 | 0.781 | POSITIVE |
| PER30_04_2019 | S267 | 0.787 | POSITIVE |
| PER30_04_2019 | T268 | 0.724 | POSITIVE |
| PER30_04_2019 | T269 | 0.808 | POSITIVE |
| PER30_04_2019 | S270 | 0.879 | POSITIVE |
| PER30_04_2019 | S275 | 0.806 | POSITIVE |
| PER30_04_2019 | S277 | 0.837 | POSITIVE |
| PER30_04_2019 | T281 | 0.723 | POSITIVE |
| PER30_04_2019 | T282 | 0.571 | POSITIVE |
| PER30_04_2019 | S283 | 0.712 | POSITIVE |
| PER30_04_2019 | T288 | 0.629 | POSITIVE |
| PER30_04_2019 | S291 | 0.835 | POSITIVE |
| PER30_04_2019 | T292 | 0.657 | POSITIVE |
| PER30_04_2019 | T293 | 0.577 | POSITIVE |
| PER30_04_2019 | S294 | 0.758 | POSITIVE |
| PER30_04_2019 | S299 | 0.804 | POSITIVE |
| PER30_04_2019 | S301 | 0.468 |  |
| PER30_04_2019 | T305 | 0.389 |  |
| PER30_04_2019 | T306 | 0.299 |  |
| PER30_04_2019 | S307 | 0.539 | POSITIVE |
| PER30_04_2019 | S311 | 0.446 |  |
| PER30_04_2019 | S313 | 0.467 |  |
| PER30_04_2019 | S315 | 0.363 |  |
| PER30_04_2019 | S316 | 0.252 |  |
| PER30_04_2019 | S317 | 0.274 |  |
| PER30_04_2019 | T319 | 0.065 |  |
| PER23_07_2018 | T219 | 0.984 | POSITIVE |
| PER23_07_2018 | T220 | 0.961 | POSITIVE |
| PER23_07_2018 | T227 | 0.969 | POSITIVE |
| PER23_07_2018 | T228 | 0.950 | POSITIVE |
| PER23_07_2018 | T231 | 0.971 | POSITIVE |
| PER23_07_2018 | T235 | 0.889 | POSITIVE |
| PER23_07_2018 | T238 | 0.774 | POSITIVE |
| PER23_07_2018 | T239 | 0.653 | POSITIVE |
| PER23_07_2018 | T241 | 0.612 | POSITIVE |
| PER23_07_2018 | S243 | 0.746 | POSITIVE |
| PER23_07_2018 | T245 | 0.742 | POSITIVE |
| PER23_07_2018 | T246 | 0.616 | POSITIVE |
| PER23_07_2018 | T249 | 0.799 | POSITIVE |
| PER23_07_2018 | S250 | 0.529 | POSITIVE |
| PER23_07_2018 | T252 | 0.651 | POSITIVE |
| PER23_07_2018 | T259 | 0.783 | POSITIVE |
| PER23_07_2018 | S260 | 0.731 | POSITIVE |
| PER23_07_2018 | T264 | 0.818 | POSITIVE |
| PER23_07_2018 | S267 | 0.806 | POSITIVE |
| PER23_07_2018 | T268 | 0.782 | POSITIVE |
| PER23_07_2018 | T269 | 0.789 | POSITIVE |
| PER23_07_2018 | S270 | 0.829 | POSITIVE |
| PER23_07_2018 | S275 | 0.713 | POSITIVE |
| PER23_07_2018 | S277 | 0.909 | POSITIVE |
| PER23_07_2018 | T281 | 0.827 | POSITIVE |
| PER23_07_2018 | T282 | 0.757 | POSITIVE |
| PER23_07_2018 | S283 | 0.850 | POSITIVE |
| PER23_07_2018 | T288 | 0.832 | POSITIVE |
| PER23_07_2018 | S291 | 0.927 | POSITIVE |
| PER23_07_2018 | T292 | 0.844 | POSITIVE |
| PER23_07_2018 | T293 | 0.776 | POSITIVE |
| PER23_07_2018 | S294 | 0.893 | POSITIVE |
| PER23_07_2018 | S299 | 0.917 | POSITIVE |
| PER23_07_2018 | S301 | 0.768 | POSITIVE |
| PER23_07_2018 | T305 | 0.640 | POSITIVE |
| PER23_07_2018 | T306 | 0.536 | POSITIVE |
| PER23_07_2018 | S307 | 0.588 | POSITIVE |
| PER23_07_2018 | S311 | 0.657 | POSITIVE |
| PER23_07_2018 | S315 | 0.590 | POSITIVE |
| PER23_07_2018 | S316 | 0.584 | POSITIVE |
| PER23_07_2018 | S317 | 0.489 |  |
| PER23_07_2018 | T319 | 0.154 |  |
| PER23_07_2018 | T320 | 0.114 |  |
| PER25_07_2018 | T219 | 0.966 | POSITIVE |
| PER25_07_2018 | T220 | 0.939 | POSITIVE |
| PER25_07_2018 | T227 | 0.969 | POSITIVE |
| PER25_07_2018 | T228 | 0.950 | POSITIVE |
| PER25_07_2018 | T231 | 0.968 | POSITIVE |
| PER25_07_2018 | T235 | 0.851 | POSITIVE |
| PER25_07_2018 | T238 | 0.771 | POSITIVE |
| PER25_07_2018 | T239 | 0.619 | POSITIVE |
| PER25_07_2018 | T241 | 0.455 |  |
| PER25_07_2018 | T245 | 0.696 | POSITIVE |
| PER25_07_2018 | T246 | 0.584 | POSITIVE |
| PER25_07_2018 | T249 | 0.714 | POSITIVE |
| PER25_07_2018 | S250 | 0.525 | POSITIVE |
| PER25_07_2018 | T252 | 0.620 | POSITIVE |
| PER25_07_2018 | T259 | 0.715 | POSITIVE |
| PER25_07_2018 | S260 | 0.722 | POSITIVE |
| PER25_07_2018 | T264 | 0.801 | POSITIVE |
| PER25_07_2018 | S267 | 0.805 | POSITIVE |
| PER25_07_2018 | T268 | 0.753 | POSITIVE |
| PER25_07_2018 | T269 | 0.856 | POSITIVE |
| PER25_07_2018 | S270 | 0.882 | POSITIVE |
| PER25_07_2018 | S275 | 0.844 | POSITIVE |
| PER25_07_2018 | S277 | 0.900 | POSITIVE |
| PER25_07_2018 | T281 | 0.792 | POSITIVE |
| PER25_07_2018 | T282 | 0.657 | POSITIVE |
| PER25_07_2018 | S283 | 0.784 | POSITIVE |
| PER25_07_2018 | T288 | 0.634 | POSITIVE |
| PER25_07_2018 | S291 | 0.839 | POSITIVE |
| PER25_07_2018 | T292 | 0.662 | POSITIVE |
| PER25_07_2018 | T293 | 0.583 | POSITIVE |
| PER25_07_2018 | S294 | 0.762 | POSITIVE |
| PER25_07_2018 | S299 | 0.808 | POSITIVE |
| PER25_07_2018 | S301 | 0.474 |  |
| PER25_07_2018 | T305 | 0.395 |  |
| PER25_07_2018 | T306 | 0.304 |  |
| PER25_07_2018 | S307 | 0.545 | POSITIVE |
| PER25_07_2018 | S311 | 0.450 |  |
| PER25_07_2018 | S313 | 0.471 |  |
| PER25_07_2018 | S315 | 0.367 |  |
| PER25_07_2018 | S316 | 0.254 |  |
| PER25_07_2018 | S317 | 0.276 |  |
| PER25_07_2018 | T319 | 0.065 |  |
| PER14_06_2018 | T219 | 0.968 | POSITIVE |
| PER14_06_2018 | T220 | 0.941 | POSITIVE |
| PER14_06_2018 | T227 | 0.969 | POSITIVE |
| PER14_06_2018 | T228 | 0.947 | POSITIVE |
| PER14_06_2018 | T231 | 0.967 | POSITIVE |
| PER14_06_2018 | T235 | 0.815 | POSITIVE |
| PER14_06_2018 | T238 | 0.710 | POSITIVE |
| PER14_06_2018 | T239 | 0.549 | POSITIVE |
| PER14_06_2018 | T241 | 0.363 |  |
| PER14_06_2018 | T245 | 0.603 | POSITIVE |
| PER14_06_2018 | T246 | 0.444 |  |
| PER14_06_2018 | T249 | 0.527 | POSITIVE |
| PER14_06_2018 | T252 | 0.598 | POSITIVE |
| PER14_06_2018 | T259 | 0.548 | POSITIVE |
| PER14_06_2018 | S260 | 0.550 | POSITIVE |
| PER14_06_2018 | T264 | 0.629 | POSITIVE |
| PER14_06_2018 | T268 | 0.494 |  |
| PER14_06_2018 | T269 | 0.755 | POSITIVE |
| PER14_06_2018 | S270 | 0.864 | POSITIVE |
| PER14_06_2018 | S275 | 0.805 | POSITIVE |
| PER14_06_2018 | S277 | 0.785 | POSITIVE |
| PER14_06_2018 | T281 | 0.637 | POSITIVE |
| PER14_06_2018 | T282 | 0.500 | POSITIVE |
| PER14_06_2018 | S283 | 0.553 | POSITIVE |
| PER14_06_2018 | T288 | 0.481 |  |
| PER14_06_2018 | S291 | 0.776 | POSITIVE |
| PER14_06_2018 | T292 | 0.407 |  |
| PER14_06_2018 | S294 | 0.628 | POSITIVE |
| PER14_06_2018 | S299 | 0.678 | POSITIVE |
| PER14_06_2018 | S301 | 0.448 |  |
| PER14_06_2018 | T305 | 0.401 |  |
| PER14_06_2018 | T306 | 0.290 |  |
| PER14_06_2018 | S307 | 0.545 | POSITIVE |
| PER14_06_2018 | S311 | 0.490 |  |
| PER14_06_2018 | S313 | 0.511 | POSITIVE |
| PER14_06_2018 | S315 | 0.369 |  |
| PER14_06_2018 | S316 | 0.263 |  |
| PER14_06_2018 | S317 | 0.286 |  |
| PER14_06_2018 | T319 | 0.069 |  |
| PER43_09_2019 | T219 | 0.968 | POSITIVE |
| PER43_09_2019 | T220 | 0.941 | POSITIVE |
| PER43_09_2019 | T227 | 0.969 | POSITIVE |
| PER43_09_2019 | T228 | 0.947 | POSITIVE |
| PER43_09_2019 | T231 | 0.967 | POSITIVE |
| PER43_09_2019 | T235 | 0.815 | POSITIVE |
| PER43_09_2019 | T238 | 0.710 | POSITIVE |
| PER43_09_2019 | T239 | 0.549 | POSITIVE |
| PER43_09_2019 | T241 | 0.363 |  |
| PER43_09_2019 | T245 | 0.603 | POSITIVE |
| PER43_09_2019 | T246 | 0.444 |  |
| PER43_09_2019 | T249 | 0.527 | POSITIVE |
| PER43_09_2019 | T252 | 0.598 | POSITIVE |
| PER43_09_2019 | T259 | 0.548 | POSITIVE |
| PER43_09_2019 | S260 | 0.550 | POSITIVE |
| PER43_09_2019 | T264 | 0.629 | POSITIVE |
| PER43_09_2019 | T268 | 0.494 |  |
| PER43_09_2019 | T269 | 0.755 | POSITIVE |
| PER43_09_2019 | S270 | 0.864 | POSITIVE |
| PER43_09_2019 | S275 | 0.805 | POSITIVE |
| PER43_09_2019 | S277 | 0.785 | POSITIVE |
| PER43_09_2019 | T281 | 0.637 | POSITIVE |
| PER43_09_2019 | T282 | 0.500 | POSITIVE |
| PER43_09_2019 | S283 | 0.553 | POSITIVE |
| PER43_09_2019 | T288 | 0.481 |  |
| PER43_09_2019 | S291 | 0.776 | POSITIVE |
| PER43_09_2019 | T292 | 0.407 |  |
| PER43_09_2019 | S294 | 0.628 | POSITIVE |
| PER43_09_2019 | S299 | 0.678 | POSITIVE |
| PER43_09_2019 | S301 | 0.448 |  |
| PER43_09_2019 | T305 | 0.401 |  |
| PER43_09_2019 | T306 | 0.290 |  |
| PER43_09_2019 | S307 | 0.545 | POSITIVE |
| PER43_09_2019 | S311 | 0.490 |  |
| PER43_09_2019 | S313 | 0.511 | POSITIVE |
| PER43_09_2019 | S315 | 0.369 |  |
| PER43_09_2019 | S316 | 0.263 |  |
| PER43_09_2019 | S317 | 0.286 |  |
| PER43_09_2019 | T319 | 0.069 |  |
| PER34_07_2019 | T219 | 0.966 | POSITIVE |
| PER34_07_2019 | T220 | 0.938 | POSITIVE |
| PER34_07_2019 | T227 | 0.968 | POSITIVE |
| PER34_07_2019 | T228 | 0.949 | POSITIVE |
| PER34_07_2019 | T231 | 0.968 | POSITIVE |
| PER34_07_2019 | T235 | 0.848 | POSITIVE |
| PER34_07_2019 | T238 | 0.768 | POSITIVE |
| PER34_07_2019 | T239 | 0.615 | POSITIVE |
| PER34_07_2019 | T241 | 0.450 |  |
| PER34_07_2019 | T245 | 0.691 | POSITIVE |
| PER34_07_2019 | T246 | 0.580 | POSITIVE |
| PER34_07_2019 | T249 | 0.711 | POSITIVE |
| PER34_07_2019 | S250 | 0.522 | POSITIVE |
| PER34_07_2019 | T252 | 0.620 | POSITIVE |
| PER34_07_2019 | T259 | 0.715 | POSITIVE |
| PER34_07_2019 | S260 | 0.718 | POSITIVE |
| PER34_07_2019 | T264 | 0.781 | POSITIVE |
| PER34_07_2019 | S267 | 0.787 | POSITIVE |
| PER34_07_2019 | T268 | 0.724 | POSITIVE |
| PER34_07_2019 | T269 | 0.808 | POSITIVE |
| PER34_07_2019 | S270 | 0.879 | POSITIVE |
| PER34_07_2019 | S275 | 0.806 | POSITIVE |
| PER34_07_2019 | S277 | 0.837 | POSITIVE |
| PER34_07_2019 | T281 | 0.723 | POSITIVE |
| PER34_07_2019 | T282 | 0.571 | POSITIVE |
| PER34_07_2019 | S283 | 0.712 | POSITIVE |
| PER34_07_2019 | T288 | 0.629 | POSITIVE |
| PER34_07_2019 | S291 | 0.835 | POSITIVE |
| PER34_07_2019 | T292 | 0.657 | POSITIVE |
| PER34_07_2019 | T293 | 0.577 | POSITIVE |
| PER34_07_2019 | S294 | 0.758 | POSITIVE |
| PER34_07_2019 | S299 | 0.804 | POSITIVE |
| PER34_07_2019 | S301 | 0.468 |  |
| PER34_07_2019 | T305 | 0.389 |  |
| PER34_07_2019 | T306 | 0.299 |  |
| PER34_07_2019 | S307 | 0.539 | POSITIVE |
| PER34_07_2019 | S311 | 0.446 |  |
| PER34_07_2019 | S313 | 0.467 |  |
| PER34_07_2019 | S315 | 0.363 |  |
| PER34_07_2019 | S316 | 0.252 |  |
| PER34_07_2019 | S317 | 0.274 |  |
| PER34_07_2019 | T319 | 0.065 |  |
| JN257693_A.D | T219 | 0.971 | POSITIVE |
| JN257693_A.D | T220 | 0.942 | POSITIVE |
| JN257693_A.D | T227 | 0.969 | POSITIVE |
| JN257693_A.D | T228 | 0.948 | POSITIVE |
| JN257693_A.D | T231 | 0.969 | POSITIVE |
| JN257693_A.D | T235 | 0.843 | POSITIVE |
| JN257693_A.D | T238 | 0.758 | POSITIVE |
| JN257693_A.D | T239 | 0.600 | POSITIVE |
| JN257693_A.D | T241 | 0.446 |  |
| JN257693_A.D | T245 | 0.686 | POSITIVE |
| JN257693_A.D | T246 | 0.570 | POSITIVE |
| JN257693_A.D | T249 | 0.698 | POSITIVE |
| JN257693_A.D | S250 | 0.520 | POSITIVE |
| JN257693_A.D | T252 | 0.616 | POSITIVE |
| JN257693_A.D | T259 | 0.718 | POSITIVE |
| JN257693_A.D | S260 | 0.732 | POSITIVE |
| JN257693_A.D | T264 | 0.750 | POSITIVE |
| JN257693_A.D | S267 | 0.753 | POSITIVE |
| JN257693_A.D | T268 | 0.700 | POSITIVE |
| JN257693_A.D | T269 | 0.773 | POSITIVE |
| JN257693_A.D | S270 | 0.804 | POSITIVE |
| JN257693_A.D | S275 | 0.591 | POSITIVE |
| JN257693_A.D | S277 | 0.814 | POSITIVE |
| JN257693_A.D | T281 | 0.721 | POSITIVE |
| JN257693_A.D | T282 | 0.543 | POSITIVE |
| JN257693_A.D | S283 | 0.611 | POSITIVE |
| JN257693_A.D | T288 | 0.478 |  |
| JN257693_A.D | S291 | 0.696 | POSITIVE |
| JN257693_A.D | T292 | 0.487 |  |
| JN257693_A.D | T293 | 0.384 |  |
| JN257693_A.D | S294 | 0.462 |  |
| JN257693_A.D | S299 | 0.442 |  |
| JN257693_A.D | S301 | 0.326 |  |
| JN257693_A.D | T305 | 0.221 |  |
| JN257693_A.D | T306 | 0.191 |  |
| JN257693_A.D | S307 | 0.240 |  |
| JN257693_A.D | S311 | 0.436 |  |
| JN257693_A.D | S313 | 0.436 |  |
| JN257693_A.D | S315 | 0.367 |  |
| JN257693_A.D | S316 | 0.345 |  |
| JN257693_A.D | S317 | 0.299 |  |
| JN257693_A.D | T319 | 0.100 |  |
| JN257693_A.D | T320 | 0.056 |  |
| PER35_07_2019 | T219 | 0.966 | POSITIVE |
| PER35_07_2019 | T220 | 0.938 | POSITIVE |
| PER35_07_2019 | T227 | 0.968 | POSITIVE |
| PER35_07_2019 | T228 | 0.949 | POSITIVE |
| PER35_07_2019 | T231 | 0.968 | POSITIVE |
| PER35_07_2019 | T235 | 0.848 | POSITIVE |
| PER35_07_2019 | T238 | 0.768 | POSITIVE |
| PER35_07_2019 | T239 | 0.615 | POSITIVE |
| PER35_07_2019 | T241 | 0.450 |  |
| PER35_07_2019 | T245 | 0.691 | POSITIVE |
| PER35_07_2019 | T246 | 0.580 | POSITIVE |
| PER35_07_2019 | T249 | 0.711 | POSITIVE |
| PER35_07_2019 | S250 | 0.522 | POSITIVE |
| PER35_07_2019 | T252 | 0.620 | POSITIVE |
| PER35_07_2019 | T259 | 0.715 | POSITIVE |
| PER35_07_2019 | S260 | 0.718 | POSITIVE |
| PER35_07_2019 | T264 | 0.781 | POSITIVE |
| PER35_07_2019 | S267 | 0.787 | POSITIVE |
| PER35_07_2019 | T268 | 0.724 | POSITIVE |
| PER35_07_2019 | T269 | 0.808 | POSITIVE |
| PER35_07_2019 | S270 | 0.879 | POSITIVE |
| PER35_07_2019 | S275 | 0.806 | POSITIVE |
| PER35_07_2019 | S277 | 0.837 | POSITIVE |
| PER35_07_2019 | T281 | 0.723 | POSITIVE |
| PER35_07_2019 | T282 | 0.571 | POSITIVE |
| PER35_07_2019 | S283 | 0.712 | POSITIVE |
| PER35_07_2019 | T288 | 0.629 | POSITIVE |
| PER35_07_2019 | S291 | 0.835 | POSITIVE |
| PER35_07_2019 | T292 | 0.657 | POSITIVE |
| PER35_07_2019 | T293 | 0.577 | POSITIVE |
| PER35_07_2019 | S294 | 0.758 | POSITIVE |
| PER35_07_2019 | S299 | 0.804 | POSITIVE |
| PER35_07_2019 | S301 | 0.468 |  |
| PER35_07_2019 | T305 | 0.389 |  |
| PER35_07_2019 | T306 | 0.299 |  |
| PER35_07_2019 | S307 | 0.539 | POSITIVE |
| PER35_07_2019 | S311 | 0.446 |  |
| PER35_07_2019 | S313 | 0.467 |  |
| PER35_07_2019 | S315 | 0.363 |  |
| PER35_07_2019 | S316 | 0.252 |  |
| PER35_07_2019 | S317 | 0.274 |  |
| PER35_07_2019 | T319 | 0.065 |  |
| PER1_05_2009 | T219 | 0.970 | POSITIVE |
| PER1_05_2009 | T220 | 0.941 | POSITIVE |
| PER1_05_2009 | T227 | 0.971 | POSITIVE |
| PER1_05_2009 | T228 | 0.951 | POSITIVE |
| PER1_05_2009 | T231 | 0.969 | POSITIVE |
| PER1_05_2009 | T235 | 0.852 | POSITIVE |
| PER1_05_2009 | T238 | 0.817 | POSITIVE |
| PER1_05_2009 | T239 | 0.597 | POSITIVE |
| PER1_05_2009 | T241 | 0.462 |  |
| PER1_05_2009 | T245 | 0.745 | POSITIVE |
| PER1_05_2009 | T246 | 0.580 | POSITIVE |
| PER1_05_2009 | T249 | 0.782 | POSITIVE |
| PER1_05_2009 | S250 | 0.634 | POSITIVE |
| PER1_05_2009 | T252 | 0.622 | POSITIVE |
| PER1_05_2009 | T259 | 0.707 | POSITIVE |
| PER1_05_2009 | S260 | 0.720 | POSITIVE |
| PER1_05_2009 | T264 | 0.740 | POSITIVE |
| PER1_05_2009 | S267 | 0.772 | POSITIVE |
| PER1_05_2009 | T268 | 0.700 | POSITIVE |
| PER1_05_2009 | T269 | 0.777 | POSITIVE |
| PER1_05_2009 | S270 | 0.793 | POSITIVE |
| PER1_05_2009 | S275 | 0.662 | POSITIVE |
| PER1_05_2009 | S277 | 0.885 | POSITIVE |
| PER1_05_2009 | T281 | 0.757 | POSITIVE |
| PER1_05_2009 | T282 | 0.633 | POSITIVE |
| PER1_05_2009 | S283 | 0.765 | POSITIVE |
| PER1_05_2009 | T288 | 0.528 | POSITIVE |
| PER1_05_2009 | S291 | 0.640 | POSITIVE |
| PER1_05_2009 | T292 | 0.740 | POSITIVE |
| PER1_05_2009 | T293 | 0.466 |  |
| PER1_05_2009 | S294 | 0.627 | POSITIVE |
| PER1_05_2009 | T305 | 0.158 |  |
| PER1_05_2009 | T306 | 0.232 |  |
| PER1_05_2009 | S315 | 0.330 |  |
| PER1_05_2009 | S316 | 0.292 |  |
| PER1_05_2009 | T319 | 0.051 |  |
| PER26_08_2018 | T219 | 0.966 | POSITIVE |
| PER26_08_2018 | T220 | 0.938 | POSITIVE |
| PER26_08_2018 | T227 | 0.968 | POSITIVE |
| PER26_08_2018 | T228 | 0.949 | POSITIVE |
| PER26_08_2018 | T231 | 0.968 | POSITIVE |
| PER26_08_2018 | T235 | 0.848 | POSITIVE |
| PER26_08_2018 | T238 | 0.768 | POSITIVE |
| PER26_08_2018 | T239 | 0.615 | POSITIVE |
| PER26_08_2018 | T241 | 0.450 |  |
| PER26_08_2018 | T245 | 0.691 | POSITIVE |
| PER26_08_2018 | T246 | 0.580 | POSITIVE |
| PER26_08_2018 | T249 | 0.711 | POSITIVE |
| PER26_08_2018 | S250 | 0.522 | POSITIVE |
| PER26_08_2018 | T252 | 0.620 | POSITIVE |
| PER26_08_2018 | T259 | 0.715 | POSITIVE |
| PER26_08_2018 | S260 | 0.718 | POSITIVE |
| PER26_08_2018 | T264 | 0.781 | POSITIVE |
| PER26_08_2018 | S267 | 0.787 | POSITIVE |
| PER26_08_2018 | T268 | 0.724 | POSITIVE |
| PER26_08_2018 | T269 | 0.808 | POSITIVE |
| PER26_08_2018 | S270 | 0.879 | POSITIVE |
| PER26_08_2018 | S275 | 0.806 | POSITIVE |
| PER26_08_2018 | S277 | 0.837 | POSITIVE |
| PER26_08_2018 | T281 | 0.723 | POSITIVE |
| PER26_08_2018 | T282 | 0.571 | POSITIVE |
| PER26_08_2018 | S283 | 0.712 | POSITIVE |
| PER26_08_2018 | T288 | 0.629 | POSITIVE |
| PER26_08_2018 | S291 | 0.835 | POSITIVE |
| PER26_08_2018 | T292 | 0.657 | POSITIVE |
| PER26_08_2018 | T293 | 0.577 | POSITIVE |
| PER26_08_2018 | S294 | 0.758 | POSITIVE |
| PER26_08_2018 | S299 | 0.804 | POSITIVE |
| PER26_08_2018 | S301 | 0.468 |  |
| PER26_08_2018 | T305 | 0.389 |  |
| PER26_08_2018 | T306 | 0.299 |  |
| PER26_08_2018 | S307 | 0.539 | POSITIVE |
| PER26_08_2018 | S311 | 0.446 |  |
| PER26_08_2018 | S313 | 0.467 |  |
| PER26_08_2018 | S315 | 0.363 |  |
| PER26_08_2018 | S316 | 0.252 |  |
| PER26_08_2018 | S317 | 0.274 |  |
| PER26_08_2018 | T319 | 0.065 |  |
| PER6_03_2010 | T219 | 0.971 | POSITIVE |
| PER6_03_2010 | T220 | 0.942 | POSITIVE |
| PER6_03_2010 | T227 | 0.969 | POSITIVE |
| PER6_03_2010 | T228 | 0.947 | POSITIVE |
| PER6_03_2010 | T231 | 0.966 | POSITIVE |
| PER6_03_2010 | T235 | 0.827 | POSITIVE |
| PER6_03_2010 | T238 | 0.766 | POSITIVE |
| PER6_03_2010 | T239 | 0.635 | POSITIVE |
| PER6_03_2010 | T245 | 0.628 | POSITIVE |
| PER6_03_2010 | T246 | 0.508 | POSITIVE |
| PER6_03_2010 | T249 | 0.707 | POSITIVE |
| PER6_03_2010 | S250 | 0.627 | POSITIVE |
| PER6_03_2010 | T252 | 0.599 | POSITIVE |
| PER6_03_2010 | T259 | 0.717 | POSITIVE |
| PER6_03_2010 | S260 | 0.716 | POSITIVE |
| PER6_03_2010 | T264 | 0.740 | POSITIVE |
| PER6_03_2010 | S267 | 0.751 | POSITIVE |
| PER6_03_2010 | T268 | 0.690 | POSITIVE |
| PER6_03_2010 | T269 | 0.765 | POSITIVE |
| PER6_03_2010 | S270 | 0.791 | POSITIVE |
| PER6_03_2010 | S275 | 0.576 | POSITIVE |
| PER6_03_2010 | S277 | 0.804 | POSITIVE |
| PER6_03_2010 | T281 | 0.701 | POSITIVE |
| PER6_03_2010 | T282 | 0.538 | POSITIVE |
| PER6_03_2010 | S283 | 0.604 | POSITIVE |
| PER6_03_2010 | T288 | 0.472 |  |
| PER6_03_2010 | S291 | 0.691 | POSITIVE |
| PER6_03_2010 | T292 | 0.480 |  |
| PER6_03_2010 | T293 | 0.377 |  |
| PER6_03_2010 | S294 | 0.455 |  |
| PER6_03_2010 | S299 | 0.435 |  |
| PER6_03_2010 | S301 | 0.319 |  |
| PER6_03_2010 | T305 | 0.216 |  |
| PER6_03_2010 | T306 | 0.187 |  |
| PER6_03_2010 | S307 | 0.235 |  |
| PER6_03_2010 | S311 | 0.432 |  |
| PER6_03_2010 | S313 | 0.432 |  |
| PER6_03_2010 | S315 | 0.363 |  |
| PER6_03_2010 | S316 | 0.342 |  |
| PER6_03_2010 | S317 | 0.297 |  |
| PER6_03_2010 | T319 | 0.100 |  |
| PER6_03_2010 | T320 | 0.055 |  |
| PER19_06_2018 | T219 | 0.968 | POSITIVE |
| PER19_06_2018 | T220 | 0.941 | POSITIVE |
| PER19_06_2018 | T227 | 0.969 | POSITIVE |
| PER19_06_2018 | T228 | 0.947 | POSITIVE |
| PER19_06_2018 | T231 | 0.967 | POSITIVE |
| PER19_06_2018 | T235 | 0.815 | POSITIVE |
| PER19_06_2018 | T238 | 0.710 | POSITIVE |
| PER19_06_2018 | T239 | 0.549 | POSITIVE |
| PER19_06_2018 | T241 | 0.363 |  |
| PER19_06_2018 | T245 | 0.603 | POSITIVE |
| PER19_06_2018 | T246 | 0.444 |  |
| PER19_06_2018 | T249 | 0.527 | POSITIVE |
| PER19_06_2018 | T252 | 0.598 | POSITIVE |
| PER19_06_2018 | T259 | 0.548 | POSITIVE |
| PER19_06_2018 | S260 | 0.550 | POSITIVE |
| PER19_06_2018 | T264 | 0.629 | POSITIVE |
| PER19_06_2018 | T268 | 0.494 |  |
| PER19_06_2018 | T269 | 0.755 | POSITIVE |
| PER19_06_2018 | S270 | 0.864 | POSITIVE |
| PER19_06_2018 | S275 | 0.805 | POSITIVE |
| PER19_06_2018 | S277 | 0.785 | POSITIVE |
| PER19_06_2018 | T281 | 0.637 | POSITIVE |
| PER19_06_2018 | T282 | 0.500 | POSITIVE |
| PER19_06_2018 | S283 | 0.553 | POSITIVE |
| PER19_06_2018 | T288 | 0.481 |  |
| PER19_06_2018 | S291 | 0.776 | POSITIVE |
| PER19_06_2018 | T292 | 0.407 |  |
| PER19_06_2018 | S294 | 0.628 | POSITIVE |
| PER19_06_2018 | S299 | 0.678 | POSITIVE |
| PER19_06_2018 | S301 | 0.448 |  |
| PER19_06_2018 | T305 | 0.401 |  |
| PER19_06_2018 | T306 | 0.290 |  |
| PER19_06_2018 | S307 | 0.545 | POSITIVE |
| PER19_06_2018 | S311 | 0.490 |  |
| PER19_06_2018 | S313 | 0.511 | POSITIVE |
| PER19_06_2018 | S315 | 0.369 |  |
| PER19_06_2018 | S316 | 0.263 |  |
| PER19_06_2018 | S317 | 0.286 |  |
| PER19_06_2018 | T319 | 0.069 |  |
| PER49_09_2019 | T219 | 0.968 | POSITIVE |
| PER49_09_2019 | T220 | 0.941 | POSITIVE |
| PER49_09_2019 | T227 | 0.969 | POSITIVE |
| PER49_09_2019 | T228 | 0.947 | POSITIVE |
| PER49_09_2019 | T231 | 0.967 | POSITIVE |
| PER49_09_2019 | T235 | 0.815 | POSITIVE |
| PER49_09_2019 | T238 | 0.710 | POSITIVE |
| PER49_09_2019 | T239 | 0.549 | POSITIVE |
| PER49_09_2019 | T241 | 0.363 |  |
| PER49_09_2019 | T245 | 0.603 | POSITIVE |
| PER49_09_2019 | T246 | 0.444 |  |
| PER49_09_2019 | T249 | 0.527 | POSITIVE |
| PER49_09_2019 | T252 | 0.598 | POSITIVE |
| PER49_09_2019 | T259 | 0.548 | POSITIVE |
| PER49_09_2019 | S260 | 0.550 | POSITIVE |
| PER49_09_2019 | T264 | 0.629 | POSITIVE |
| PER49_09_2019 | T268 | 0.494 |  |
| PER49_09_2019 | T269 | 0.755 | POSITIVE |
| PER49_09_2019 | S270 | 0.864 | POSITIVE |
| PER49_09_2019 | S275 | 0.805 | POSITIVE |
| PER49_09_2019 | S277 | 0.785 | POSITIVE |
| PER49_09_2019 | T281 | 0.637 | POSITIVE |
| PER49_09_2019 | T282 | 0.500 | POSITIVE |
| PER49_09_2019 | S283 | 0.553 | POSITIVE |
| PER49_09_2019 | T288 | 0.481 |  |
| PER49_09_2019 | S291 | 0.776 | POSITIVE |
| PER49_09_2019 | T292 | 0.407 |  |
| PER49_09_2019 | S294 | 0.628 | POSITIVE |
| PER49_09_2019 | S299 | 0.678 | POSITIVE |
| PER49_09_2019 | S301 | 0.448 |  |
| PER49_09_2019 | T305 | 0.401 |  |
| PER49_09_2019 | T306 | 0.290 |  |
| PER49_09_2019 | S307 | 0.545 | POSITIVE |
| PER49_09_2019 | S311 | 0.490 |  |
| PER49_09_2019 | S313 | 0.511 | POSITIVE |
| PER49_09_2019 | S315 | 0.369 |  |
| PER49_09_2019 | S316 | 0.263 |  |
| PER49_09_2019 | S317 | 0.286 |  |
| PER49_09_2019 | T319 | 0.069 |  |
| PER28_04_2019 | T219 | 0.966 | POSITIVE |
| PER28_04_2019 | T220 | 0.939 | POSITIVE |
| PER28_04_2019 | T227 | 0.969 | POSITIVE |
| PER28_04_2019 | T228 | 0.948 | POSITIVE |
| PER28_04_2019 | T231 | 0.967 | POSITIVE |
| PER28_04_2019 | T235 | 0.822 | POSITIVE |
| PER28_04_2019 | T238 | 0.722 | POSITIVE |
| PER28_04_2019 | T239 | 0.567 | POSITIVE |
| PER28_04_2019 | T241 | 0.368 |  |
| PER28_04_2019 | T245 | 0.612 | POSITIVE |
| PER28_04_2019 | T246 | 0.454 |  |
| PER28_04_2019 | T249 | 0.545 | POSITIVE |
| PER28_04_2019 | T252 | 0.620 | POSITIVE |
| PER28_04_2019 | T259 | 0.634 | POSITIVE |
| PER28_04_2019 | S260 | 0.607 | POSITIVE |
| PER28_04_2019 | T264 | 0.767 | POSITIVE |
| PER28_04_2019 | S267 | 0.771 | POSITIVE |
| PER28_04_2019 | T268 | 0.705 | POSITIVE |
| PER28_04_2019 | T269 | 0.797 | POSITIVE |
| PER28_04_2019 | S270 | 0.874 | POSITIVE |
| PER28_04_2019 | S275 | 0.806 | POSITIVE |
| PER28_04_2019 | S277 | 0.826 | POSITIVE |
| PER28_04_2019 | T281 | 0.653 | POSITIVE |
| PER28_04_2019 | T282 | 0.509 | POSITIVE |
| PER28_04_2019 | S283 | 0.554 | POSITIVE |
| PER28_04_2019 | T288 | 0.477 |  |
| PER28_04_2019 | S291 | 0.770 | POSITIVE |
| PER28_04_2019 | T292 | 0.398 |  |
| PER28_04_2019 | S294 | 0.605 | POSITIVE |
| PER28_04_2019 | S299 | 0.654 | POSITIVE |
| PER28_04_2019 | S301 | 0.416 |  |
| PER28_04_2019 | T305 | 0.363 |  |
| PER28_04_2019 | T306 | 0.277 |  |
| PER28_04_2019 | S307 | 0.511 | POSITIVE |
| PER28_04_2019 | S311 | 0.458 |  |
| PER28_04_2019 | S313 | 0.466 |  |
| PER28_04_2019 | S315 | 0.362 |  |
| PER28_04_2019 | S316 | 0.258 |  |
| PER28_04_2019 | S317 | 0.272 |  |
| PER28_04_2019 | T319 | 0.063 |  |
| PER20_06_2018 | T219 | 0.966 | POSITIVE |
| PER20_06_2018 | T220 | 0.939 | POSITIVE |
| PER20_06_2018 | T227 | 0.969 | POSITIVE |
| PER20_06_2018 | T228 | 0.948 | POSITIVE |
| PER20_06_2018 | T231 | 0.967 | POSITIVE |
| PER20_06_2018 | T235 | 0.822 | POSITIVE |
| PER20_06_2018 | T238 | 0.722 | POSITIVE |
| PER20_06_2018 | T239 | 0.567 | POSITIVE |
| PER20_06_2018 | T241 | 0.368 |  |
| PER20_06_2018 | T245 | 0.612 | POSITIVE |
| PER20_06_2018 | T246 | 0.454 |  |
| PER20_06_2018 | T249 | 0.545 | POSITIVE |
| PER20_06_2018 | T252 | 0.620 | POSITIVE |
| PER20_06_2018 | T259 | 0.634 | POSITIVE |
| PER20_06_2018 | S260 | 0.607 | POSITIVE |
| PER20_06_2018 | T264 | 0.767 | POSITIVE |
| PER20_06_2018 | S267 | 0.771 | POSITIVE |
| PER20_06_2018 | T268 | 0.705 | POSITIVE |
| PER20_06_2018 | T269 | 0.797 | POSITIVE |
| PER20_06_2018 | S270 | 0.874 | POSITIVE |
| PER20_06_2018 | S275 | 0.806 | POSITIVE |
| PER20_06_2018 | S277 | 0.826 | POSITIVE |
| PER20_06_2018 | T281 | 0.653 | POSITIVE |
| PER20_06_2018 | T282 | 0.509 | POSITIVE |
| PER20_06_2018 | S283 | 0.554 | POSITIVE |
| PER20_06_2018 | T288 | 0.477 |  |
| PER20_06_2018 | S291 | 0.770 | POSITIVE |
| PER20_06_2018 | T292 | 0.398 |  |
| PER20_06_2018 | S294 | 0.605 | POSITIVE |
| PER20_06_2018 | S299 | 0.654 | POSITIVE |
| PER20_06_2018 | S301 | 0.416 |  |
| PER20_06_2018 | T305 | 0.363 |  |
| PER20_06_2018 | T306 | 0.277 |  |
| PER20_06_2018 | S307 | 0.511 | POSITIVE |
| PER20_06_2018 | S311 | 0.458 |  |
| PER20_06_2018 | S313 | 0.466 |  |
| PER20_06_2018 | S315 | 0.362 |  |
| PER20_06_2018 | S316 | 0.258 |  |
| PER20_06_2018 | S317 | 0.272 |  |
| PER20_06_2018 | T319 | 0.063 |  |
| PER27_10_2018 | T219 | 0.966 | POSITIVE |
| PER27_10_2018 | T220 | 0.938 | POSITIVE |
| PER27_10_2018 | T227 | 0.968 | POSITIVE |
| PER27_10_2018 | T228 | 0.949 | POSITIVE |
| PER27_10_2018 | T231 | 0.968 | POSITIVE |
| PER27_10_2018 | T235 | 0.848 | POSITIVE |
| PER27_10_2018 | T238 | 0.768 | POSITIVE |
| PER27_10_2018 | T239 | 0.615 | POSITIVE |
| PER27_10_2018 | T241 | 0.450 |  |
| PER27_10_2018 | T245 | 0.691 | POSITIVE |
| PER27_10_2018 | T246 | 0.580 | POSITIVE |
| PER27_10_2018 | T249 | 0.711 | POSITIVE |
| PER27_10_2018 | S250 | 0.522 | POSITIVE |
| PER27_10_2018 | T252 | 0.620 | POSITIVE |
| PER27_10_2018 | T259 | 0.715 | POSITIVE |
| PER27_10_2018 | S260 | 0.718 | POSITIVE |
| PER27_10_2018 | T264 | 0.781 | POSITIVE |
| PER27_10_2018 | S267 | 0.787 | POSITIVE |
| PER27_10_2018 | T268 | 0.724 | POSITIVE |
| PER27_10_2018 | T269 | 0.808 | POSITIVE |
| PER27_10_2018 | S270 | 0.879 | POSITIVE |
| PER27_10_2018 | S275 | 0.806 | POSITIVE |
| PER27_10_2018 | S277 | 0.837 | POSITIVE |
| PER27_10_2018 | T281 | 0.723 | POSITIVE |
| PER27_10_2018 | T282 | 0.571 | POSITIVE |
| PER27_10_2018 | S283 | 0.712 | POSITIVE |
| PER27_10_2018 | T288 | 0.629 | POSITIVE |
| PER27_10_2018 | S291 | 0.835 | POSITIVE |
| PER27_10_2018 | T292 | 0.657 | POSITIVE |
| PER27_10_2018 | T293 | 0.577 | POSITIVE |
| PER27_10_2018 | S294 | 0.758 | POSITIVE |
| PER27_10_2018 | S299 | 0.804 | POSITIVE |
| PER27_10_2018 | S301 | 0.468 |  |
| PER27_10_2018 | T305 | 0.389 |  |
| PER27_10_2018 | T306 | 0.299 |  |
| PER27_10_2018 | S307 | 0.539 | POSITIVE |
| PER27_10_2018 | S311 | 0.446 |  |
| PER27_10_2018 | S313 | 0.467 |  |
| PER27_10_2018 | S315 | 0.363 |  |
| PER27_10_2018 | S316 | 0.252 |  |
| PER27_10_2018 | S317 | 0.274 |  |
| PER27_10_2018 | T319 | 0.065 |  |
| PER12_05_2010 | T219 | 0.966 | POSITIVE |
| PER12_05_2010 | T220 | 0.939 | POSITIVE |
| PER12_05_2010 | T227 | 0.969 | POSITIVE |
| PER12_05_2010 | T228 | 0.950 | POSITIVE |
| PER12_05_2010 | T231 | 0.968 | POSITIVE |
| PER12_05_2010 | T235 | 0.851 | POSITIVE |
| PER12_05_2010 | T238 | 0.771 | POSITIVE |
| PER12_05_2010 | T239 | 0.619 | POSITIVE |
| PER12_05_2010 | T241 | 0.455 |  |
| PER12_05_2010 | T245 | 0.696 | POSITIVE |
| PER12_05_2010 | T246 | 0.584 | POSITIVE |
| PER12_05_2010 | T249 | 0.714 | POSITIVE |
| PER12_05_2010 | S250 | 0.525 | POSITIVE |
| PER12_05_2010 | T252 | 0.620 | POSITIVE |
| PER12_05_2010 | T259 | 0.715 | POSITIVE |
| PER12_05_2010 | S260 | 0.722 | POSITIVE |
| PER12_05_2010 | T264 | 0.801 | POSITIVE |
| PER12_05_2010 | S267 | 0.805 | POSITIVE |
| PER12_05_2010 | T268 | 0.753 | POSITIVE |
| PER12_05_2010 | T269 | 0.856 | POSITIVE |
| PER12_05_2010 | S270 | 0.882 | POSITIVE |
| PER12_05_2010 | S275 | 0.844 | POSITIVE |
| PER12_05_2010 | S277 | 0.900 | POSITIVE |
| PER12_05_2010 | T281 | 0.792 | POSITIVE |
| PER12_05_2010 | T282 | 0.657 | POSITIVE |
| PER12_05_2010 | S283 | 0.784 | POSITIVE |
| PER12_05_2010 | T288 | 0.634 | POSITIVE |
| PER12_05_2010 | S291 | 0.839 | POSITIVE |
| PER12_05_2010 | T292 | 0.662 | POSITIVE |
| PER12_05_2010 | T293 | 0.583 | POSITIVE |
| PER12_05_2010 | S294 | 0.762 | POSITIVE |
| PER12_05_2010 | S299 | 0.808 | POSITIVE |
| PER12_05_2010 | S301 | 0.474 |  |
| PER12_05_2010 | T305 | 0.395 |  |
| PER12_05_2010 | T306 | 0.304 |  |
| PER12_05_2010 | S307 | 0.545 | POSITIVE |
| PER12_05_2010 | S311 | 0.450 |  |
| PER12_05_2010 | S313 | 0.471 |  |
| PER12_05_2010 | S315 | 0.367 |  |
| PER12_05_2010 | S316 | 0.254 |  |
| PER12_05_2010 | S317 | 0.276 |  |
| PER12_05_2010 | T319 | 0.065 |  |
| PER8_05_2010 | T219 | 0.966 | POSITIVE |
| PER8_05_2010 | T220 | 0.939 | POSITIVE |
| PER8_05_2010 | T227 | 0.969 | POSITIVE |
| PER8_05_2010 | T228 | 0.950 | POSITIVE |
| PER8_05_2010 | T231 | 0.968 | POSITIVE |
| PER8_05_2010 | T235 | 0.851 | POSITIVE |
| PER8_05_2010 | T238 | 0.771 | POSITIVE |
| PER8_05_2010 | T239 | 0.619 | POSITIVE |
| PER8_05_2010 | T241 | 0.455 |  |
| PER8_05_2010 | T245 | 0.696 | POSITIVE |
| PER8_05_2010 | T246 | 0.584 | POSITIVE |
| PER8_05_2010 | T249 | 0.714 | POSITIVE |
| PER8_05_2010 | S250 | 0.525 | POSITIVE |
| PER8_05_2010 | T252 | 0.620 | POSITIVE |
| PER8_05_2010 | T259 | 0.715 | POSITIVE |
| PER8_05_2010 | S260 | 0.722 | POSITIVE |
| PER8_05_2010 | T264 | 0.801 | POSITIVE |
| PER8_05_2010 | S267 | 0.805 | POSITIVE |
| PER8_05_2010 | T268 | 0.753 | POSITIVE |
| PER8_05_2010 | T269 | 0.856 | POSITIVE |
| PER8_05_2010 | S270 | 0.882 | POSITIVE |
| PER8_05_2010 | S275 | 0.844 | POSITIVE |
| PER8_05_2010 | S277 | 0.900 | POSITIVE |
| PER8_05_2010 | T281 | 0.792 | POSITIVE |
| PER8_05_2010 | T282 | 0.657 | POSITIVE |
| PER8_05_2010 | S283 | 0.784 | POSITIVE |
| PER8_05_2010 | T288 | 0.634 | POSITIVE |
| PER8_05_2010 | S291 | 0.839 | POSITIVE |
| PER8_05_2010 | T292 | 0.662 | POSITIVE |
| PER8_05_2010 | T293 | 0.583 | POSITIVE |
| PER8_05_2010 | S294 | 0.762 | POSITIVE |
| PER8_05_2010 | S299 | 0.808 | POSITIVE |
| PER8_05_2010 | S301 | 0.474 |  |
| PER8_05_2010 | T305 | 0.395 |  |
| PER8_05_2010 | T306 | 0.304 |  |
| PER8_05_2010 | S307 | 0.545 | POSITIVE |
| PER8_05_2010 | S311 | 0.450 |  |
| PER8_05_2010 | S313 | 0.471 |  |
| PER8_05_2010 | S315 | 0.367 |  |
| PER8_05_2010 | S316 | 0.254 |  |
| PER8_05_2010 | S317 | 0.276 |  |
| PER8_05_2010 | T319 | 0.065 |  |
| PER50_09_2019 | T219 | 0.968 | POSITIVE |
| PER50_09_2019 | T220 | 0.941 | POSITIVE |
| PER50_09_2019 | T227 | 0.969 | POSITIVE |
| PER50_09_2019 | T228 | 0.947 | POSITIVE |
| PER50_09_2019 | T231 | 0.967 | POSITIVE |
| PER50_09_2019 | T235 | 0.815 | POSITIVE |
| PER50_09_2019 | T238 | 0.710 | POSITIVE |
| PER50_09_2019 | T239 | 0.549 | POSITIVE |
| PER50_09_2019 | T241 | 0.363 |  |
| PER50_09_2019 | T245 | 0.603 | POSITIVE |
| PER50_09_2019 | T246 | 0.444 |  |
| PER50_09_2019 | T249 | 0.527 | POSITIVE |
| PER50_09_2019 | T252 | 0.598 | POSITIVE |
| PER50_09_2019 | T259 | 0.548 | POSITIVE |
| PER50_09_2019 | S260 | 0.550 | POSITIVE |
| PER50_09_2019 | T264 | 0.629 | POSITIVE |
| PER50_09_2019 | T268 | 0.494 |  |
| PER50_09_2019 | T269 | 0.755 | POSITIVE |
| PER50_09_2019 | S270 | 0.864 | POSITIVE |
| PER50_09_2019 | S275 | 0.805 | POSITIVE |
| PER50_09_2019 | S277 | 0.785 | POSITIVE |
| PER50_09_2019 | T281 | 0.637 | POSITIVE |
| PER50_09_2019 | T282 | 0.500 | POSITIVE |
| PER50_09_2019 | S283 | 0.553 | POSITIVE |
| PER50_09_2019 | T288 | 0.481 |  |
| PER50_09_2019 | S291 | 0.776 | POSITIVE |
| PER50_09_2019 | T292 | 0.407 |  |
| PER50_09_2019 | S294 | 0.628 | POSITIVE |
| PER50_09_2019 | S299 | 0.678 | POSITIVE |
| PER50_09_2019 | S301 | 0.448 |  |
| PER50_09_2019 | T305 | 0.401 |  |
| PER50_09_2019 | T306 | 0.290 |  |
| PER50_09_2019 | S307 | 0.545 | POSITIVE |
| PER50_09_2019 | S311 | 0.490 |  |
| PER50_09_2019 | S313 | 0.511 | POSITIVE |
| PER50_09_2019 | S315 | 0.369 |  |
| PER50_09_2019 | S316 | 0.263 |  |
| PER50_09_2019 | S317 | 0.286 |  |
| PER50_09_2019 | T319 | 0.069 |  |
| PER45_09_2019 | T219 | 0.968 | POSITIVE |
| PER45_09_2019 | T220 | 0.941 | POSITIVE |
| PER45_09_2019 | T227 | 0.969 | POSITIVE |
| PER45_09_2019 | T228 | 0.947 | POSITIVE |
| PER45_09_2019 | T231 | 0.967 | POSITIVE |
| PER45_09_2019 | T235 | 0.815 | POSITIVE |
| PER45_09_2019 | T238 | 0.710 | POSITIVE |
| PER45_09_2019 | T239 | 0.549 | POSITIVE |
| PER45_09_2019 | T241 | 0.363 |  |
| PER45_09_2019 | T245 | 0.603 | POSITIVE |
| PER45_09_2019 | T246 | 0.444 |  |
| PER45_09_2019 | T249 | 0.527 | POSITIVE |
| PER45_09_2019 | T252 | 0.598 | POSITIVE |
| PER45_09_2019 | T259 | 0.548 | POSITIVE |
| PER45_09_2019 | S260 | 0.550 | POSITIVE |
| PER45_09_2019 | T264 | 0.629 | POSITIVE |
| PER45_09_2019 | T268 | 0.494 |  |
| PER45_09_2019 | T269 | 0.755 | POSITIVE |
| PER45_09_2019 | S270 | 0.864 | POSITIVE |
| PER45_09_2019 | S275 | 0.805 | POSITIVE |
| PER45_09_2019 | S277 | 0.785 | POSITIVE |
| PER45_09_2019 | T281 | 0.637 | POSITIVE |
| PER45_09_2019 | T282 | 0.500 | POSITIVE |
| PER45_09_2019 | S283 | 0.553 | POSITIVE |
| PER45_09_2019 | T288 | 0.481 |  |
| PER45_09_2019 | S291 | 0.776 | POSITIVE |
| PER45_09_2019 | T292 | 0.407 |  |
| PER45_09_2019 | S294 | 0.628 | POSITIVE |
| PER45_09_2019 | S299 | 0.678 | POSITIVE |
| PER45_09_2019 | S301 | 0.448 |  |
| PER45_09_2019 | T305 | 0.401 |  |
| PER45_09_2019 | T306 | 0.290 |  |
| PER45_09_2019 | S307 | 0.545 | POSITIVE |
| PER45_09_2019 | S311 | 0.490 |  |
| PER45_09_2019 | S313 | 0.511 | POSITIVE |
| PER45_09_2019 | S315 | 0.369 |  |
| PER45_09_2019 | S316 | 0.263 |  |
| PER45_09_2019 | S317 | 0.286 |  |
| PER45_09_2019 | T319 | 0.069 |  |
| PER38_07_2019 | T219 | 0.966 | POSITIVE |
| PER38_07_2019 | T220 | 0.938 | POSITIVE |
| PER38_07_2019 | T227 | 0.968 | POSITIVE |
| PER38_07_2019 | T228 | 0.949 | POSITIVE |
| PER38_07_2019 | T231 | 0.968 | POSITIVE |
| PER38_07_2019 | T235 | 0.847 | POSITIVE |
| PER38_07_2019 | T238 | 0.766 | POSITIVE |
| PER38_07_2019 | T239 | 0.612 | POSITIVE |
| PER38_07_2019 | T241 | 0.447 |  |
| PER38_07_2019 | T245 | 0.689 | POSITIVE |
| PER38_07_2019 | T246 | 0.577 | POSITIVE |
| PER38_07_2019 | T249 | 0.709 | POSITIVE |
| PER38_07_2019 | S250 | 0.519 | POSITIVE |
| PER38_07_2019 | T252 | 0.617 | POSITIVE |
| PER38_07_2019 | T259 | 0.713 | POSITIVE |
| PER38_07_2019 | S260 | 0.716 | POSITIVE |
| PER38_07_2019 | T264 | 0.779 | POSITIVE |
| PER38_07_2019 | S267 | 0.786 | POSITIVE |
| PER38_07_2019 | T268 | 0.722 | POSITIVE |
| PER38_07_2019 | T269 | 0.807 | POSITIVE |
| PER38_07_2019 | S270 | 0.878 | POSITIVE |
| PER38_07_2019 | S275 | 0.804 | POSITIVE |
| PER38_07_2019 | S277 | 0.836 | POSITIVE |
| PER38_07_2019 | T281 | 0.721 | POSITIVE |
| PER38_07_2019 | T282 | 0.564 | POSITIVE |
| PER38_07_2019 | S283 | 0.705 | POSITIVE |
| PER38_07_2019 | T288 | 0.579 | POSITIVE |
| PER38_07_2019 | S291 | 0.813 | POSITIVE |
| PER38_07_2019 | T292 | 0.752 | POSITIVE |
| PER38_07_2019 | T293 | 0.565 | POSITIVE |
| PER38_07_2019 | S294 | 0.748 | POSITIVE |
| PER38_07_2019 | S299 | 0.639 | POSITIVE |
| PER38_07_2019 | T305 | 0.269 |  |
| PER38_07_2019 | T306 | 0.383 |  |
| PER38_07_2019 | S315 | 0.270 |  |
| PER38_07_2019 | S316 | 0.278 |  |
| PER18_06_2018 | T219 | 0.984 | POSITIVE |
| PER18_06_2018 | T220 | 0.961 | POSITIVE |
| PER18_06_2018 | T227 | 0.969 | POSITIVE |
| PER18_06_2018 | T228 | 0.950 | POSITIVE |
| PER18_06_2018 | T231 | 0.971 | POSITIVE |
| PER18_06_2018 | T235 | 0.889 | POSITIVE |
| PER18_06_2018 | T238 | 0.774 | POSITIVE |
| PER18_06_2018 | T239 | 0.653 | POSITIVE |
| PER18_06_2018 | T241 | 0.612 | POSITIVE |
| PER18_06_2018 | S243 | 0.746 | POSITIVE |
| PER18_06_2018 | T245 | 0.742 | POSITIVE |
| PER18_06_2018 | T246 | 0.616 | POSITIVE |
| PER18_06_2018 | T249 | 0.799 | POSITIVE |
| PER18_06_2018 | S250 | 0.529 | POSITIVE |
| PER18_06_2018 | T252 | 0.651 | POSITIVE |
| PER18_06_2018 | T259 | 0.783 | POSITIVE |
| PER18_06_2018 | S260 | 0.731 | POSITIVE |
| PER18_06_2018 | T264 | 0.818 | POSITIVE |
| PER18_06_2018 | S267 | 0.806 | POSITIVE |
| PER18_06_2018 | T268 | 0.782 | POSITIVE |
| PER18_06_2018 | T269 | 0.789 | POSITIVE |
| PER18_06_2018 | S270 | 0.829 | POSITIVE |
| PER18_06_2018 | S275 | 0.713 | POSITIVE |
| PER18_06_2018 | S277 | 0.909 | POSITIVE |
| PER18_06_2018 | T281 | 0.827 | POSITIVE |
| PER18_06_2018 | T282 | 0.757 | POSITIVE |
| PER18_06_2018 | S283 | 0.850 | POSITIVE |
| PER18_06_2018 | T288 | 0.832 | POSITIVE |
| PER18_06_2018 | S291 | 0.927 | POSITIVE |
| PER18_06_2018 | T292 | 0.844 | POSITIVE |
| PER18_06_2018 | T293 | 0.776 | POSITIVE |
| PER18_06_2018 | S294 | 0.893 | POSITIVE |
| PER18_06_2018 | S299 | 0.917 | POSITIVE |
| PER18_06_2018 | S301 | 0.768 | POSITIVE |
| PER18_06_2018 | T305 | 0.640 | POSITIVE |
| PER18_06_2018 | T306 | 0.536 | POSITIVE |
| PER18_06_2018 | S307 | 0.588 | POSITIVE |
| PER18_06_2018 | S311 | 0.657 | POSITIVE |
| PER18_06_2018 | S315 | 0.590 | POSITIVE |
| PER18_06_2018 | S316 | 0.584 | POSITIVE |
| PER18_06_2018 | S317 | 0.489 |  |
| PER18_06_2018 | T319 | 0.154 |  |
| PER18_06_2018 | T320 | 0.114 |  |
| PER16_06_2018 | T219 | 0.966 | POSITIVE |
| PER16_06_2018 | T220 | 0.938 | POSITIVE |
| PER16_06_2018 | T227 | 0.968 | POSITIVE |
| PER16_06_2018 | T228 | 0.949 | POSITIVE |
| PER16_06_2018 | T231 | 0.968 | POSITIVE |
| PER16_06_2018 | T235 | 0.848 | POSITIVE |
| PER16_06_2018 | T238 | 0.768 | POSITIVE |
| PER16_06_2018 | T239 | 0.615 | POSITIVE |
| PER16_06_2018 | T241 | 0.450 |  |
| PER16_06_2018 | T245 | 0.691 | POSITIVE |
| PER16_06_2018 | T246 | 0.580 | POSITIVE |
| PER16_06_2018 | T249 | 0.711 | POSITIVE |
| PER16_06_2018 | S250 | 0.522 | POSITIVE |
| PER16_06_2018 | T252 | 0.620 | POSITIVE |
| PER16_06_2018 | T259 | 0.715 | POSITIVE |
| PER16_06_2018 | S260 | 0.718 | POSITIVE |
| PER16_06_2018 | T264 | 0.781 | POSITIVE |
| PER16_06_2018 | S267 | 0.787 | POSITIVE |
| PER16_06_2018 | T268 | 0.724 | POSITIVE |
| PER16_06_2018 | T269 | 0.808 | POSITIVE |
| PER16_06_2018 | S270 | 0.879 | POSITIVE |
| PER16_06_2018 | S275 | 0.806 | POSITIVE |
| PER16_06_2018 | S277 | 0.837 | POSITIVE |
| PER16_06_2018 | T281 | 0.723 | POSITIVE |
| PER16_06_2018 | T282 | 0.571 | POSITIVE |
| PER16_06_2018 | S283 | 0.712 | POSITIVE |
| PER16_06_2018 | T288 | 0.629 | POSITIVE |
| PER16_06_2018 | S291 | 0.835 | POSITIVE |
| PER16_06_2018 | T292 | 0.657 | POSITIVE |
| PER16_06_2018 | T293 | 0.577 | POSITIVE |
| PER16_06_2018 | S294 | 0.758 | POSITIVE |
| PER16_06_2018 | S299 | 0.804 | POSITIVE |
| PER16_06_2018 | S301 | 0.468 |  |
| PER16_06_2018 | T305 | 0.389 |  |
| PER16_06_2018 | T306 | 0.299 |  |
| PER16_06_2018 | S307 | 0.539 | POSITIVE |
| PER16_06_2018 | S311 | 0.446 |  |
| PER16_06_2018 | S313 | 0.467 |  |
| PER16_06_2018 | S315 | 0.363 |  |
| PER16_06_2018 | S316 | 0.252 |  |
| PER16_06_2018 | S317 | 0.274 |  |
| PER16_06_2018 | T319 | 0.065 |  |
| PER29_04_2019 | T219 | 0.965 | POSITIVE |
| PER29_04_2019 | T220 | 0.935 | POSITIVE |
| PER29_04_2019 | T227 | 0.959 | POSITIVE |
| PER29_04_2019 | T228 | 0.939 | POSITIVE |
| PER29_04_2019 | T231 | 0.904 | POSITIVE |
| PER29_04_2019 | T235 | 0.648 | POSITIVE |
| PER29_04_2019 | T238 | 0.746 | POSITIVE |
| PER29_04_2019 | T239 | 0.599 | POSITIVE |
| PER29_04_2019 | T241 | 0.441 |  |
| PER29_04_2019 | T245 | 0.698 | POSITIVE |
| PER29_04_2019 | T246 | 0.579 | POSITIVE |
| PER29_04_2019 | T249 | 0.711 | POSITIVE |
| PER29_04_2019 | S250 | 0.527 | POSITIVE |
| PER29_04_2019 | T252 | 0.631 | POSITIVE |
| PER29_04_2019 | T259 | 0.723 | POSITIVE |
| PER29_04_2019 | S260 | 0.726 | POSITIVE |
| PER29_04_2019 | T264 | 0.787 | POSITIVE |
| PER29_04_2019 | S267 | 0.794 | POSITIVE |
| PER29_04_2019 | T268 | 0.732 | POSITIVE |
| PER29_04_2019 | T269 | 0.815 | POSITIVE |
| PER29_04_2019 | S270 | 0.883 | POSITIVE |
| PER29_04_2019 | S275 | 0.812 | POSITIVE |
| PER29_04_2019 | S277 | 0.842 | POSITIVE |
| PER29_04_2019 | T281 | 0.732 | POSITIVE |
| PER29_04_2019 | T282 | 0.581 | POSITIVE |
| PER29_04_2019 | S283 | 0.720 | POSITIVE |
| PER29_04_2019 | T288 | 0.639 | POSITIVE |
| PER29_04_2019 | S291 | 0.842 | POSITIVE |
| PER29_04_2019 | T292 | 0.667 | POSITIVE |
| PER29_04_2019 | T293 | 0.590 | POSITIVE |
| PER29_04_2019 | S294 | 0.768 | POSITIVE |
| PER29_04_2019 | S299 | 0.814 | POSITIVE |
| PER29_04_2019 | S301 | 0.483 |  |
| PER29_04_2019 | T305 | 0.403 |  |
| PER29_04_2019 | T306 | 0.310 |  |
| PER29_04_2019 | S307 | 0.552 | POSITIVE |
| PER29_04_2019 | S311 | 0.457 |  |
| PER29_04_2019 | S313 | 0.476 |  |
| PER29_04_2019 | S315 | 0.372 |  |
| PER29_04_2019 | S316 | 0.257 |  |
| PER29_04_2019 | S317 | 0.279 |  |
| PER29_04_2019 | T319 | 0.066 |  |
| PER42_09_2019 | T219 | 0.968 | POSITIVE |
| PER42_09_2019 | T220 | 0.941 | POSITIVE |
| PER42_09_2019 | T227 | 0.969 | POSITIVE |
| PER42_09_2019 | T228 | 0.947 | POSITIVE |
| PER42_09_2019 | T231 | 0.967 | POSITIVE |
| PER42_09_2019 | T235 | 0.815 | POSITIVE |
| PER42_09_2019 | T238 | 0.710 | POSITIVE |
| PER42_09_2019 | T239 | 0.549 | POSITIVE |
| PER42_09_2019 | T241 | 0.363 |  |
| PER42_09_2019 | T245 | 0.603 | POSITIVE |
| PER42_09_2019 | T246 | 0.444 |  |
| PER42_09_2019 | T249 | 0.527 | POSITIVE |
| PER42_09_2019 | T252 | 0.598 | POSITIVE |
| PER42_09_2019 | T259 | 0.548 | POSITIVE |
| PER42_09_2019 | S260 | 0.550 | POSITIVE |
| PER42_09_2019 | T264 | 0.629 | POSITIVE |
| PER42_09_2019 | T268 | 0.494 |  |
| PER42_09_2019 | T269 | 0.755 | POSITIVE |
| PER42_09_2019 | S270 | 0.864 | POSITIVE |
| PER42_09_2019 | S275 | 0.805 | POSITIVE |
| PER42_09_2019 | S277 | 0.785 | POSITIVE |
| PER42_09_2019 | T281 | 0.637 | POSITIVE |
| PER42_09_2019 | T282 | 0.500 | POSITIVE |
| PER42_09_2019 | S283 | 0.553 | POSITIVE |
| PER42_09_2019 | T288 | 0.481 |  |
| PER42_09_2019 | S291 | 0.776 | POSITIVE |
| PER42_09_2019 | T292 | 0.407 |  |
| PER42_09_2019 | S294 | 0.628 | POSITIVE |
| PER42_09_2019 | S299 | 0.678 | POSITIVE |
| PER42_09_2019 | S301 | 0.448 |  |
| PER42_09_2019 | T305 | 0.401 |  |
| PER42_09_2019 | T306 | 0.290 |  |
| PER42_09_2019 | S307 | 0.545 | POSITIVE |
| PER42_09_2019 | S311 | 0.490 |  |
| PER42_09_2019 | S313 | 0.511 | POSITIVE |
| PER42_09_2019 | S315 | 0.369 |  |
| PER42_09_2019 | S316 | 0.263 |  |
| PER42_09_2019 | S317 | 0.286 |  |
| PER42_09_2019 | T319 | 0.069 |  |
| PER22_06_2018 | T219 | 0.984 | POSITIVE |
| PER22_06_2018 | T220 | 0.961 | POSITIVE |
| PER22_06_2018 | T227 | 0.969 | POSITIVE |
| PER22_06_2018 | T228 | 0.950 | POSITIVE |
| PER22_06_2018 | T231 | 0.971 | POSITIVE |
| PER22_06_2018 | T235 | 0.889 | POSITIVE |
| PER22_06_2018 | T238 | 0.774 | POSITIVE |
| PER22_06_2018 | T239 | 0.653 | POSITIVE |
| PER22_06_2018 | T241 | 0.612 | POSITIVE |
| PER22_06_2018 | S243 | 0.746 | POSITIVE |
| PER22_06_2018 | T245 | 0.742 | POSITIVE |
| PER22_06_2018 | T246 | 0.616 | POSITIVE |
| PER22_06_2018 | T249 | 0.799 | POSITIVE |
| PER22_06_2018 | S250 | 0.529 | POSITIVE |
| PER22_06_2018 | T252 | 0.651 | POSITIVE |
| PER22_06_2018 | T259 | 0.783 | POSITIVE |
| PER22_06_2018 | S260 | 0.731 | POSITIVE |
| PER22_06_2018 | T264 | 0.818 | POSITIVE |
| PER22_06_2018 | S267 | 0.806 | POSITIVE |
| PER22_06_2018 | T268 | 0.782 | POSITIVE |
| PER22_06_2018 | T269 | 0.789 | POSITIVE |
| PER22_06_2018 | S270 | 0.829 | POSITIVE |
| PER22_06_2018 | S275 | 0.713 | POSITIVE |
| PER22_06_2018 | S277 | 0.909 | POSITIVE |
| PER22_06_2018 | T281 | 0.827 | POSITIVE |
| PER22_06_2018 | T282 | 0.757 | POSITIVE |
| PER22_06_2018 | S283 | 0.850 | POSITIVE |
| PER22_06_2018 | T288 | 0.832 | POSITIVE |
| PER22_06_2018 | S291 | 0.927 | POSITIVE |
| PER22_06_2018 | T292 | 0.844 | POSITIVE |
| PER22_06_2018 | T293 | 0.776 | POSITIVE |
| PER22_06_2018 | S294 | 0.893 | POSITIVE |
| PER22_06_2018 | S299 | 0.917 | POSITIVE |
| PER22_06_2018 | S301 | 0.768 | POSITIVE |
| PER22_06_2018 | T305 | 0.640 | POSITIVE |
| PER22_06_2018 | T306 | 0.536 | POSITIVE |
| PER22_06_2018 | S307 | 0.588 | POSITIVE |
| PER22_06_2018 | S311 | 0.657 | POSITIVE |
| PER22_06_2018 | S315 | 0.590 | POSITIVE |
| PER22_06_2018 | S316 | 0.584 | POSITIVE |
| PER22_06_2018 | S317 | 0.489 |  |
| PER22_06_2018 | T319 | 0.154 |  |
| PER22_06_2018 | T320 | 0.114 |  |
| PER4_11_2009 | T219 | 0.962 | POSITIVE |
| PER4_11_2009 | T220 | 0.933 | POSITIVE |
| PER4_11_2009 | T227 | 0.966 | POSITIVE |
| PER4_11_2009 | T228 | 0.947 | POSITIVE |
| PER4_11_2009 | T231 | 0.956 | POSITIVE |
| PER4_11_2009 | T235 | 0.827 | POSITIVE |
| PER4_11_2009 | T238 | 0.743 | POSITIVE |
| PER4_11_2009 | T239 | 0.587 | POSITIVE |
| PER4_11_2009 | T241 | 0.425 |  |
| PER4_11_2009 | T245 | 0.690 | POSITIVE |
| PER4_11_2009 | T246 | 0.573 | POSITIVE |
| PER4_11_2009 | T249 | 0.706 | POSITIVE |
| PER4_11_2009 | S250 | 0.523 | POSITIVE |
| PER4_11_2009 | T252 | 0.624 | POSITIVE |
| PER4_11_2009 | T259 | 0.709 | POSITIVE |
| PER4_11_2009 | S260 | 0.709 | POSITIVE |
| PER4_11_2009 | T264 | 0.821 | POSITIVE |
| PER4_11_2009 | S267 | 0.878 | POSITIVE |
| PER4_11_2009 | T268 | 0.728 | POSITIVE |
| PER4_11_2009 | T269 | 0.856 | POSITIVE |
| PER4_11_2009 | S275 | 0.822 | POSITIVE |
| PER4_11_2009 | S277 | 0.827 | POSITIVE |
| PER4_11_2009 | T281 | 0.712 | POSITIVE |
| PER4_11_2009 | T282 | 0.548 | POSITIVE |
| PER4_11_2009 | S283 | 0.682 | POSITIVE |
| PER4_11_2009 | T288 | 0.606 | POSITIVE |
| PER4_11_2009 | S291 | 0.797 | POSITIVE |
| PER4_11_2009 | T292 | 0.553 | POSITIVE |
| PER4_11_2009 | T293 | 0.489 |  |
| PER4_11_2009 | S294 | 0.670 | POSITIVE |
| PER4_11_2009 | S298 | 0.801 | POSITIVE |
| PER4_11_2009 | S299 | 0.732 | POSITIVE |
| PER4_11_2009 | S301 | 0.437 |  |
| PER4_11_2009 | T305 | 0.381 |  |
| PER4_11_2009 | T306 | 0.272 |  |
| PER4_11_2009 | S307 | 0.500 | POSITIVE |
| PER4_11_2009 | S311 | 0.444 |  |
| PER4_11_2009 | S313 | 0.463 |  |
| PER4_11_2009 | S315 | 0.358 |  |
| PER4_11_2009 | S316 | 0.254 |  |
| PER4_11_2009 | S317 | 0.260 |  |
| PER4_11_2009 | T319 | 0.062 |  |
| PER17_06_2018 | T219 | 0.966 | POSITIVE |
| PER17_06_2018 | T220 | 0.938 | POSITIVE |
| PER17_06_2018 | T227 | 0.968 | POSITIVE |
| PER17_06_2018 | T228 | 0.949 | POSITIVE |
| PER17_06_2018 | T231 | 0.968 | POSITIVE |
| PER17_06_2018 | T235 | 0.848 | POSITIVE |
| PER17_06_2018 | T238 | 0.768 | POSITIVE |
| PER17_06_2018 | T239 | 0.615 | POSITIVE |
| PER17_06_2018 | T241 | 0.450 |  |
| PER17_06_2018 | T245 | 0.691 | POSITIVE |
| PER17_06_2018 | T246 | 0.580 | POSITIVE |
| PER17_06_2018 | T249 | 0.711 | POSITIVE |
| PER17_06_2018 | S250 | 0.522 | POSITIVE |
| PER17_06_2018 | T252 | 0.620 | POSITIVE |
| PER17_06_2018 | T259 | 0.715 | POSITIVE |
| PER17_06_2018 | S260 | 0.718 | POSITIVE |
| PER17_06_2018 | T264 | 0.781 | POSITIVE |
| PER17_06_2018 | S267 | 0.787 | POSITIVE |
| PER17_06_2018 | T268 | 0.724 | POSITIVE |
| PER17_06_2018 | T269 | 0.808 | POSITIVE |
| PER17_06_2018 | S270 | 0.879 | POSITIVE |
| PER17_06_2018 | S275 | 0.806 | POSITIVE |
| PER17_06_2018 | S277 | 0.837 | POSITIVE |
| PER17_06_2018 | T281 | 0.723 | POSITIVE |
| PER17_06_2018 | T282 | 0.571 | POSITIVE |
| PER17_06_2018 | S283 | 0.712 | POSITIVE |
| PER17_06_2018 | T288 | 0.629 | POSITIVE |
| PER17_06_2018 | S291 | 0.835 | POSITIVE |
| PER17_06_2018 | T292 | 0.657 | POSITIVE |
| PER17_06_2018 | T293 | 0.577 | POSITIVE |
| PER17_06_2018 | S294 | 0.758 | POSITIVE |
| PER17_06_2018 | S299 | 0.804 | POSITIVE |
| PER17_06_2018 | S301 | 0.468 |  |
| PER17_06_2018 | T305 | 0.389 |  |
| PER17_06_2018 | T306 | 0.299 |  |
| PER17_06_2018 | S307 | 0.539 | POSITIVE |
| PER17_06_2018 | S311 | 0.446 |  |
| PER17_06_2018 | S313 | 0.467 |  |
| PER17_06_2018 | S315 | 0.363 |  |
| PER17_06_2018 | S316 | 0.252 |  |
| PER17_06_2018 | S317 | 0.274 |  |
| PER17_06_2018 | T319 | 0.065 |  |
| PER37_07_2019 | T219 | 0.966 | POSITIVE |
| PER37_07_2019 | T220 | 0.939 | POSITIVE |
| PER37_07_2019 | T227 | 0.969 | POSITIVE |
| PER37_07_2019 | T228 | 0.950 | POSITIVE |
| PER37_07_2019 | T231 | 0.968 | POSITIVE |
| PER37_07_2019 | T235 | 0.851 | POSITIVE |
| PER37_07_2019 | T238 | 0.771 | POSITIVE |
| PER37_07_2019 | T239 | 0.619 | POSITIVE |
| PER37_07_2019 | T241 | 0.455 |  |
| PER37_07_2019 | T245 | 0.696 | POSITIVE |
| PER37_07_2019 | T246 | 0.584 | POSITIVE |
| PER37_07_2019 | T249 | 0.715 | POSITIVE |
| PER37_07_2019 | S250 | 0.527 | POSITIVE |
| PER37_07_2019 | T252 | 0.624 | POSITIVE |
| PER37_07_2019 | T259 | 0.717 | POSITIVE |
| PER37_07_2019 | S260 | 0.721 | POSITIVE |
| PER37_07_2019 | T264 | 0.785 | POSITIVE |
| PER37_07_2019 | S267 | 0.794 | POSITIVE |
| PER37_07_2019 | T268 | 0.723 | POSITIVE |
| PER37_07_2019 | T269 | 0.809 | POSITIVE |
| PER37_07_2019 | S270 | 0.880 | POSITIVE |
| PER37_07_2019 | S275 | 0.769 | POSITIVE |
| PER37_07_2019 | S277 | 0.823 | POSITIVE |
| PER37_07_2019 | T281 | 0.711 | POSITIVE |
| PER37_07_2019 | T282 | 0.602 | POSITIVE |
| PER37_07_2019 | S283 | 0.726 | POSITIVE |
| PER37_07_2019 | S284 | 0.717 | POSITIVE |
| PER37_07_2019 | T288 | 0.705 | POSITIVE |
| PER37_07_2019 | S291 | 0.846 | POSITIVE |
| PER37_07_2019 | T292 | 0.663 | POSITIVE |
| PER37_07_2019 | T293 | 0.639 | POSITIVE |
| PER37_07_2019 | S294 | 0.792 | POSITIVE |
| PER37_07_2019 | S299 | 0.809 | POSITIVE |
| PER37_07_2019 | S301 | 0.526 | POSITIVE |
| PER37_07_2019 | T305 | 0.395 |  |
| PER37_07_2019 | T306 | 0.298 |  |
| PER37_07_2019 | S307 | 0.555 | POSITIVE |
| PER37_07_2019 | S311 | 0.458 |  |
| PER37_07_2019 | S313 | 0.472 |  |
| PER37_07_2019 | S315 | 0.368 |  |
| PER37_07_2019 | S316 | 0.252 |  |
| PER37_07_2019 | S317 | 0.276 |  |
| PER37_07_2019 | T319 | 0.065 |  |

**Table S6. O-glycosylation sites of the A.3.1/A.3.1.1 lineages sequences of this study**

| **Seqname** | **Position** | **Score** | **Result** |
| --- | --- | --- | --- |
| PER39_07_2019 | T219 | 0.856 | POSITIVE |
| PER39_07_2019 | T220 | 0.725 | POSITIVE |
| PER39_07_2019 | T227 | 0.780 | POSITIVE |
| PER39_07_2019 | T228 | 0.569 | POSITIVE |
| PER39_07_2019 | T230 | 0.733 | POSITIVE |
| PER39_07_2019 | T235 | 0.345 |  |
| PER39_07_2019 | T238 | 0.142 |  |
| PER39_07_2019 | T239 | 0.147 |  |
| PER39_07_2019 | T241 | 0.147 |  |
| PER39_07_2019 | T245 | 0.236 |  |
| PER39_07_2019 | T246 | 0.182 |  |
| PER39_07_2019 | T249 | 0.171 |  |
| PER39_07_2019 | S250 | 0.136 |  |
| PER39_07_2019 | T252 | 0.189 |  |
| PER39_07_2019 | T253 | 0.269 |  |
| PER39_07_2019 | T259 | 0.267 |  |
| PER39_07_2019 | S260 | 0.265 |  |
| PER39_07_2019 | T264 | 0.221 |  |
| PER39_07_2019 | S267 | 0.214 |  |
| PER39_07_2019 | T268 | 0.159 |  |
| PER39_07_2019 | T269 | 0.267 |  |
| PER39_07_2019 | S270 | 0.271 |  |
| PER39_07_2019 | S275 | 0.173 |  |
| PER39_07_2019 | S277 | 0.170 |  |
| PER39_07_2019 | T281 | 0.204 |  |
| PER39_07_2019 | T282 | 0.135 |  |
| PER39_07_2019 | S283 | 0.169 |  |
| PER39_07_2019 | S287 | 0.389 |  |
| PER39_07_2019 | S289 | 0.244 |  |
| PER39_07_2019 | S291 | 0.276 |  |
| PER39_07_2019 | S292 | 0.146 |  |
| PER39_07_2019 | S293 | 0.119 |  |
| PER39_07_2019 | T295 | 0.025 |  |
| PER39_07_2019 | T296 | 0.016 |  |
| PER5_03_2010 | T219 | 0.857 | POSITIVE |
| PER5_03_2010 | T220 | 0.727 | POSITIVE |
| PER5_03_2010 | T227 | 0.781 | POSITIVE |
| PER5_03_2010 | T228 | 0.571 | POSITIVE |
| PER5_03_2010 | T230 | 0.734 | POSITIVE |
| PER5_03_2010 | T235 | 0.346 |  |
| PER5_03_2010 | T238 | 0.143 |  |
| PER5_03_2010 | T239 | 0.147 |  |
| PER5_03_2010 | T241 | 0.128 |  |
| PER5_03_2010 | T245 | 0.242 |  |
| PER5_03_2010 | T246 | 0.164 |  |
| PER5_03_2010 | T249 | 0.233 |  |
| PER5_03_2010 | S250 | 0.155 |  |
| PER5_03_2010 | T252 | 0.270 |  |
| PER5_03_2010 | T253 | 0.288 |  |
| PER5_03_2010 | T259 | 0.235 |  |
| PER5_03_2010 | S260 | 0.257 |  |
| PER5_03_2010 | T264 | 0.223 |  |
| PER5_03_2010 | S267 | 0.215 |  |
| PER5_03_2010 | T268 | 0.161 |  |
| PER5_03_2010 | T269 | 0.269 |  |
| PER5_03_2010 | S270 | 0.271 |  |
| PER5_03_2010 | S275 | 0.174 |  |
| PER5_03_2010 | S277 | 0.172 |  |
| PER5_03_2010 | T281 | 0.206 |  |
| PER5_03_2010 | T282 | 0.137 |  |
| PER5_03_2010 | S283 | 0.171 |  |
| PER5_03_2010 | S287 | 0.392 |  |
| PER5_03_2010 | S289 | 0.246 |  |
| PER5_03_2010 | S291 | 0.278 |  |
| PER5_03_2010 | S292 | 0.147 |  |
| PER5_03_2010 | S293 | 0.120 |  |
| PER5_03_2010 | T295 | 0.025 |  |
| PER5_03_2010 | T296 | 0.016 |  |
| MH181905 | T219 | 0.842 | POSITIVE |
| MH181905 | T220 | 0.712 | POSITIVE |
| MH181905 | T227 | 0.712 | POSITIVE |
| MH181905 | T228 | 0.609 | POSITIVE |
| MH181905 | T230 | 0.721 | POSITIVE |
| MH181905 | T235 | 0.325 |  |
| MH181905 | T238 | 0.144 |  |
| MH181905 | T239 | 0.163 |  |
| MH181905 | T241 | 0.074 |  |
| MH181905 | T245 | 0.159 |  |
| MH181905 | T246 | 0.104 |  |
| MH181905 | T249 | 0.214 |  |
| MH181905 | S250 | 0.138 |  |
| MH181905 | T252 | 0.260 |  |
| MH181905 | T253 | 0.272 |  |
| MH181905 | T259 | 0.239 |  |
| MH181905 | S260 | 0.261 |  |
| MH181905 | T264 | 0.229 |  |
| MH181905 | S267 | 0.221 |  |
| MH181905 | T268 | 0.166 |  |
| MH181905 | T269 | 0.273 |  |
| MH181905 | S270 | 0.279 |  |
| MH181905 | S275 | 0.180 |  |
| MH181905 | S277 | 0.177 |  |
| MH181905 | T281 | 0.202 |  |
| MH181905 | T282 | 0.129 |  |
| MH181905 | S283 | 0.164 |  |
| MH181905 | S287 | 0.297 |  |
| MH181905 | S289 | 0.147 |  |
| MH181905 | S291 | 0.202 |  |
| MH181905 | S292 | 0.073 |  |
| MH181905 | S293 | 0.053 |  |
| MH181905 | T295 | 0.011 |  |
| PER9_05_2010 | T219 | 0.855 | POSITIVE |
| PER9_05_2010 | T220 | 0.721 | POSITIVE |
| PER9_05_2010 | T227 | 0.802 | POSITIVE |
| PER9_05_2010 | T228 | 0.606 | POSITIVE |
| PER9_05_2010 | T230 | 0.703 | POSITIVE |
| PER9_05_2010 | T235 | 0.398 |  |
| PER9_05_2010 | T238 | 0.202 |  |
| PER9_05_2010 | T239 | 0.223 |  |
| PER9_05_2010 | T245 | 0.221 |  |
| PER9_05_2010 | T246 | 0.147 |  |
| PER9_05_2010 | T249 | 0.208 |  |
| PER9_05_2010 | S250 | 0.159 |  |
| PER9_05_2010 | T252 | 0.284 |  |
| PER9_05_2010 | T253 | 0.294 |  |
| PER9_05_2010 | T259 | 0.226 |  |
| PER9_05_2010 | S260 | 0.249 |  |
| PER9_05_2010 | T264 | 0.215 |  |
| PER9_05_2010 | S267 | 0.208 |  |
| PER9_05_2010 | T268 | 0.154 |  |
| PER9_05_2010 | T269 | 0.260 |  |
| PER9_05_2010 | S270 | 0.264 |  |
| PER9_05_2010 | S275 | 0.169 |  |
| PER9_05_2010 | S277 | 0.166 |  |
| PER9_05_2010 | T281 | 0.200 |  |
| PER9_05_2010 | T282 | 0.132 |  |
| PER9_05_2010 | S283 | 0.165 |  |
| PER9_05_2010 | S287 | 0.385 |  |
| PER9_05_2010 | S289 | 0.241 |  |
| PER9_05_2010 | S291 | 0.273 |  |
| PER9_05_2010 | S292 | 0.145 |  |
| PER9_05_2010 | S293 | 0.118 |  |
| PER9_05_2010 | T295 | 0.025 |  |
| PER9_05_2010 | T296 | 0.016 |  |
| MF001050 | T219 | 0.855 | POSITIVE |
| MF001050 | T220 | 0.721 | POSITIVE |
| MF001050 | T227 | 0.786 | POSITIVE |
| MF001050 | T228 | 0.554 | POSITIVE |
| MF001050 | T230 | 0.799 | POSITIVE |
| MF001050 | T235 | 0.463 |  |
| MF001050 | T238 | 0.224 |  |
| MF001050 | T239 | 0.232 |  |
| MF001050 | T241 | 0.101 |  |
| MF001050 | T245 | 0.201 |  |
| MF001050 | T246 | 0.146 |  |
| MF001050 | T249 | 0.215 |  |
| MF001050 | S250 | 0.105 |  |
| MF001050 | T252 | 0.268 |  |
| MF001050 | T253 | 0.278 |  |
| MF001050 | T259 | 0.236 |  |
| MF001050 | S260 | 0.259 |  |
| MF001050 | T264 | 0.225 |  |
| MF001050 | S267 | 0.218 |  |
| MF001050 | T268 | 0.162 |  |
| MF001050 | T269 | 0.271 |  |
| MF001050 | S270 | 0.275 |  |
| MF001050 | S275 | 0.177 |  |
| MF001050 | S277 | 0.173 |  |
| MF001050 | T281 | 0.207 |  |
| MF001050 | T282 | 0.138 |  |
| MF001050 | S283 | 0.171 |  |
| MF001050 | S287 | 0.394 |  |
| MF001050 | S289 | 0.246 |  |
| MF001050 | S291 | 0.279 |  |
| MF001050 | S292 | 0.148 |  |
| MF001050 | S293 | 0.120 |  |
| MF001050 | T295 | 0.025 |  |
| MF001050 | T296 | 0.016 |  |
| PER10_05_2010 | T219 | 0.857 | POSITIVE |
| PER10_05_2010 | T220 | 0.727 | POSITIVE |
| PER10_05_2010 | T227 | 0.781 | POSITIVE |
| PER10_05_2010 | T228 | 0.571 | POSITIVE |
| PER10_05_2010 | T230 | 0.734 | POSITIVE |
| PER10_05_2010 | T235 | 0.346 |  |
| PER10_05_2010 | T238 | 0.143 |  |
| PER10_05_2010 | T239 | 0.147 |  |
| PER10_05_2010 | T241 | 0.128 |  |
| PER10_05_2010 | T245 | 0.242 |  |
| PER10_05_2010 | T246 | 0.164 |  |
| PER10_05_2010 | T249 | 0.233 |  |
| PER10_05_2010 | S250 | 0.155 |  |
| PER10_05_2010 | T252 | 0.270 |  |
| PER10_05_2010 | T253 | 0.288 |  |
| PER10_05_2010 | T259 | 0.235 |  |
| PER10_05_2010 | S260 | 0.257 |  |
| PER10_05_2010 | T264 | 0.223 |  |
| PER10_05_2010 | S267 | 0.215 |  |
| PER10_05_2010 | T268 | 0.161 |  |
| PER10_05_2010 | T269 | 0.269 |  |
| PER10_05_2010 | S270 | 0.271 |  |
| PER10_05_2010 | S275 | 0.174 |  |
| PER10_05_2010 | S277 | 0.172 |  |
| PER10_05_2010 | T281 | 0.206 |  |
| PER10_05_2010 | T282 | 0.137 |  |
| PER10_05_2010 | S283 | 0.171 |  |
| PER10_05_2010 | S287 | 0.392 |  |
| PER10_05_2010 | S289 | 0.246 |  |
| PER10_05_2010 | S291 | 0.278 |  |
| PER10_05_2010 | S292 | 0.147 |  |
| PER10_05_2010 | S293 | 0.120 |  |
| PER10_05_2010 | T295 | 0.025 |  |
| PER10_05_2010 | T296 | 0.016 |  |
| AB470478_A.3.1 | T219 | 0.854 | POSITIVE |
| AB470478_A.3.1 | T220 | 0.722 | POSITIVE |
| AB470478_A.3.1 | T227 | 0.777 | POSITIVE |
| AB470478_A.3.1 | T228 | 0.565 | POSITIVE |
| AB470478_A.3.1 | T230 | 0.728 | POSITIVE |
| AB470478_A.3.1 | T235 | 0.339 |  |
| AB470478_A.3.1 | T238 | 0.138 |  |
| AB470478_A.3.1 | T239 | 0.141 |  |
| AB470478_A.3.1 | T241 | 0.123 |  |
| AB470478_A.3.1 | T245 | 0.243 |  |
| AB470478_A.3.1 | T246 | 0.162 |  |
| AB470478_A.3.1 | T249 | 0.230 |  |
| AB470478_A.3.1 | S250 | 0.141 |  |
| AB470478_A.3.1 | T252 | 0.202 |  |
| AB470478_A.3.1 | T253 | 0.262 |  |
| AB470478_A.3.1 | T259 | 0.234 |  |
| AB470478_A.3.1 | T264 | 0.169 |  |
| AB470478_A.3.1 | S267 | 0.186 |  |
| AB470478_A.3.1 | T268 | 0.145 |  |
| AB470478_A.3.1 | T269 | 0.247 |  |
| AB470478_A.3.1 | S270 | 0.243 |  |
| AB470478_A.3.1 | S275 | 0.186 |  |
| AB470478_A.3.1 | S277 | 0.168 |  |
| AB470478_A.3.1 | T281 | 0.205 |  |
| AB470478_A.3.1 | T282 | 0.132 |  |
| AB470478_A.3.1 | S283 | 0.161 |  |
| AB470478_A.3.1 | S287 | 0.386 |  |
| AB470478_A.3.1 | S289 | 0.241 |  |
| AB470478_A.3.1 | S291 | 0.274 |  |
| AB470478_A.3.1 | S292 | 0.145 |  |
| AB470478_A.3.1 | S293 | 0.118 |  |
| AB470478_A.3.1 | T295 | 0.025 |  |
| AB470478_A.3.1 | T296 | 0.016 |  |
| PER40_08_2019 | T219 | 0.856 | POSITIVE |
| PER40_08_2019 | T220 | 0.725 | POSITIVE |
| PER40_08_2019 | T227 | 0.780 | POSITIVE |
| PER40_08_2019 | T228 | 0.569 | POSITIVE |
| PER40_08_2019 | T230 | 0.733 | POSITIVE |
| PER40_08_2019 | T235 | 0.345 |  |
| PER40_08_2019 | T238 | 0.142 |  |
| PER40_08_2019 | T239 | 0.147 |  |
| PER40_08_2019 | T241 | 0.147 |  |
| PER40_08_2019 | T245 | 0.236 |  |
| PER40_08_2019 | T246 | 0.182 |  |
| PER40_08_2019 | T249 | 0.171 |  |
| PER40_08_2019 | S250 | 0.136 |  |
| PER40_08_2019 | T252 | 0.189 |  |
| PER40_08_2019 | T253 | 0.269 |  |
| PER40_08_2019 | T259 | 0.267 |  |
| PER40_08_2019 | S260 | 0.265 |  |
| PER40_08_2019 | T264 | 0.221 |  |
| PER40_08_2019 | S267 | 0.214 |  |
| PER40_08_2019 | T268 | 0.159 |  |
| PER40_08_2019 | T269 | 0.267 |  |
| PER40_08_2019 | S270 | 0.271 |  |
| PER40_08_2019 | S275 | 0.173 |  |
| PER40_08_2019 | S277 | 0.170 |  |
| PER40_08_2019 | T281 | 0.204 |  |
| PER40_08_2019 | T282 | 0.135 |  |
| PER40_08_2019 | S283 | 0.169 |  |
| PER40_08_2019 | S287 | 0.389 |  |
| PER40_08_2019 | S289 | 0.244 |  |
| PER40_08_2019 | S291 | 0.276 |  |
| PER40_08_2019 | S292 | 0.146 |  |
| PER40_08_2019 | S293 | 0.119 |  |
| PER40_08_2019 | T295 | 0.025 |  |
| PER40_08_2019 | T296 | 0.016 |  |
| PER41_08_2019 | T219 | 0.856 | POSITIVE |
| PER41_08_2019 | T220 | 0.725 | POSITIVE |
| PER41_08_2019 | T227 | 0.780 | POSITIVE |
| PER41_08_2019 | T228 | 0.569 | POSITIVE |
| PER41_08_2019 | T230 | 0.733 | POSITIVE |
| PER41_08_2019 | T235 | 0.345 |  |
| PER41_08_2019 | T238 | 0.142 |  |
| PER41_08_2019 | T239 | 0.147 |  |
| PER41_08_2019 | T241 | 0.147 |  |
| PER41_08_2019 | T245 | 0.236 |  |
| PER41_08_2019 | T246 | 0.182 |  |
| PER41_08_2019 | T249 | 0.171 |  |
| PER41_08_2019 | S250 | 0.136 |  |
| PER41_08_2019 | T252 | 0.189 |  |
| PER41_08_2019 | T253 | 0.269 |  |
| PER41_08_2019 | T259 | 0.267 |  |
| PER41_08_2019 | S260 | 0.265 |  |
| PER41_08_2019 | T264 | 0.221 |  |
| PER41_08_2019 | S267 | 0.214 |  |
| PER41_08_2019 | T268 | 0.159 |  |
| PER41_08_2019 | T269 | 0.267 |  |
| PER41_08_2019 | S270 | 0.271 |  |
| PER41_08_2019 | S275 | 0.173 |  |
| PER41_08_2019 | S277 | 0.170 |  |
| PER41_08_2019 | T281 | 0.204 |  |
| PER41_08_2019 | T282 | 0.135 |  |
| PER41_08_2019 | S283 | 0.169 |  |
| PER41_08_2019 | S287 | 0.389 |  |
| PER41_08_2019 | S289 | 0.244 |  |
| PER41_08_2019 | S291 | 0.276 |  |
| PER41_08_2019 | S292 | 0.146 |  |
| PER41_08_2019 | S293 | 0.119 |  |
| PER41_08_2019 | T295 | 0.025 |  |
| PER41_08_2019 | T296 | 0.016 |  |
| KC297389 | T219 | 0.856 | POSITIVE |
| KC297389 | T220 | 0.725 | POSITIVE |
| KC297389 | T227 | 0.780 | POSITIVE |
| KC297389 | T228 | 0.570 | POSITIVE |
| KC297389 | T230 | 0.733 | POSITIVE |
| KC297389 | T235 | 0.345 |  |
| KC297389 | T238 | 0.142 |  |
| KC297389 | T239 | 0.146 |  |
| KC297389 | T241 | 0.127 |  |
| KC297389 | T245 | 0.241 |  |
| KC297389 | T246 | 0.163 |  |
| KC297389 | T249 | 0.232 |  |
| KC297389 | S250 | 0.153 |  |
| KC297389 | T252 | 0.268 |  |
| KC297389 | T253 | 0.286 |  |
| KC297389 | T259 | 0.233 |  |
| KC297389 | S260 | 0.255 |  |
| KC297389 | T264 | 0.222 |  |
| KC297389 | S267 | 0.212 |  |
| KC297389 | T268 | 0.153 |  |
| KC297389 | T269 | 0.252 |  |
| KC297389 | S270 | 0.262 |  |
| KC297389 | S275 | 0.185 |  |
| KC297389 | S277 | 0.193 |  |
| KC297389 | T281 | 0.160 |  |
| KC297389 | T282 | 0.235 |  |
| KC297389 | S283 | 0.162 |  |
| KC297389 | S287 | 0.413 |  |
| KC297389 | S289 | 0.251 |  |
| KC297389 | S291 | 0.324 |  |
| KC297389 | S292 | 0.162 |  |
| KC297389 | S293 | 0.145 |  |
| KC297389 | T295 | 0.024 |  |
| KC297389 | T296 | 0.014 |  |
| PER11_05_2010 | T219 | 0.853 | POSITIVE |
| PER11_05_2010 | T220 | 0.713 | POSITIVE |
| PER11_05_2010 | T227 | 0.803 | POSITIVE |
| PER11_05_2010 | T228 | 0.601 | POSITIVE |
| PER11_05_2010 | T230 | 0.708 | POSITIVE |
| PER11_05_2010 | T235 | 0.412 |  |
| PER11_05_2010 | T238 | 0.206 |  |
| PER11_05_2010 | T239 | 0.227 |  |
| PER11_05_2010 | T245 | 0.217 |  |
| PER11_05_2010 | T246 | 0.136 |  |
| PER11_05_2010 | T249 | 0.151 |  |
| PER11_05_2010 | S250 | 0.103 |  |
| PER11_05_2010 | T252 | 0.148 |  |
| PER11_05_2010 | T253 | 0.183 |  |
| PER11_05_2010 | S260 | 0.131 |  |
| PER11_05_2010 | T264 | 0.155 |  |
| PER11_05_2010 | S267 | 0.143 |  |
| PER11_05_2010 | T268 | 0.123 |  |
| PER11_05_2010 | T269 | 0.171 |  |
| PER11_05_2010 | S270 | 0.244 |  |
| PER11_05_2010 | S275 | 0.172 |  |
| PER11_05_2010 | S277 | 0.177 |  |
| PER11_05_2010 | T281 | 0.211 |  |
| PER11_05_2010 | T282 | 0.137 |  |
| PER11_05_2010 | S283 | 0.167 |  |
| PER11_05_2010 | S287 | 0.396 |  |
| PER11_05_2010 | S289 | 0.249 |  |
| PER11_05_2010 | S291 | 0.282 |  |
| PER11_05_2010 | S292 | 0.147 |  |
| PER11_05_2010 | S293 | 0.122 |  |
| PER11_05_2010 | T295 | 0.026 |  |
| PER11_05_2010 | T296 | 0.016 |  |
